# Distance‐Dependence of Photo‐CIDNP in Biomimetic Tryptophan–Flavin Diads

**DOI:** 10.1002/anie.202510116

**Published:** 2025-08-05

**Authors:** Tobias Theiss, Guzel Musabirova, Luca Gerhards, Irina S. Anisimova, Ben G. E. Zoller, Thi Ngoc Ha Nguyen, Dmitry Denisov, Anton Schmidt, Sabrina Panter, Stefan Weber, Christoph Tegenkamp, Ilia A. Solov'yov, Jörg Matysik, Tanja Gulder

**Affiliations:** ^1^ Department of Organic Chemistry Leipzig University Johannisallee 29 04103 Leipzig Germany; ^2^ Department of Analytical Chemistry Leipzig University Linnéstr. 3 04103 Leipzig Germany; ^3^ Institute of Physics Carl‐von‐Ossietzky‐University Carl‐von‐Ossietzky‐Str. 9–11 26129 Oldenburg Germany; ^4^ Organic Chemistry Saarland University 66123 Saarbrücken Germany; ^5^ Department of Physics Chemnitz University of Technology Reichenhainer Str. 70 09126 Chemnitz Germany; ^6^ Institute of Physical Chemistry Albert‐Ludwigs‐Universität Freiburg Albertstr. 21 79104 Freiburg Germany; ^7^ Research Center Neurosensory Science Carl‐von‐Ossietzky‐University Carl‐von‐Ossietzky‐Str. 9–11 26129 Oldenburg Germany; ^8^ Center for Nanoscale Dynamics (CENAD) Carl‐von‐Ossietzky‐University Carl‐von‐Ossietzky‐Str. 9–11 26129 Oldenburg Germany; ^9^ Synthesis of Natural‐Product Derived Drugs Helmholtz Institute for Pharmaceutical Research Saarland (HIPS) Helmholtz Centre for Infection Research (HZI) 66123 Saarbrücken Germany

**Keywords:** Hyperpolarization, NMR, Oligoprolines, Photo‐CIDNP, Radicals, Spectroscopy

## Abstract

Nuclear magnetic resonance (NMR) and magnetic resonance imaging (MRI) are essential tools in natural and life sciences, but their low sensitivity often hampers their applicability. Photochemically induced dynamic nuclear polarization (photo‐CIDNP) offers a versatile and mild method to overcome this limitation. Here, we report on structure‐photo‐CIDNP relationship studies in liquid‐state NMR utilizing a holistic approach. We synthesized biomimetic tryptophan–flavin diads with varying linker lengths composed of conformationally rigid oligoproline units. The predominant polyproline II (PPII) helical structure ensures consistent donor–acceptor distances. Photo‐CIDNP NMR experiments revealed significant hyperpolarization effects, particularly in diads with six proline units, mimicking the spatial arrangement found in natural photoactive proteins. Our findings highlight the potential of these biomimetic diads to enhance nuclear hyperpolarization in NMR spectroscopy. This work provides valuable insights into the design of diads for efficient photo‐CIDNP generation, paving the way for advanced studies in modern NMR and bio‐MRI.

## Graphical abstract

NMR and MRI are crucial in sciences, but their low sensitivity limits their use. Photochemically induced dynamic nuclear polarization (photo‐CIDNP) offers a solution. This study explores structure‐photo‐CIDNP relationships in liquid‐state NMR using biomimetic tryptophan–flavin diads with varying oligoproline linker lengths. Findings show significant hyperpolarization effects, especially in diads with six proline units. Insights from this work pave the way for advanced NMR and bio‐MRI studies.

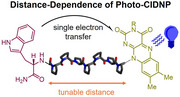

## Introduction

Nuclear magnetic resonance (NMR) spectroscopy and magnetic resonance imaging (MRI) are essential tools because of their exceptional capacity to detect changes in the local environment of a specific probe, even at the atomic level and within the interior of samples.^[^ ^1^ ^]^ These techniques find applications in many disciplines, from physics and chemistry to material sciences, structural biology, and medicine.^[^ ^2^, ^3^, ^4^, ^5^, ^6^ ^]^ However, a severe limitation of magnetic resonance is its low sensitivity compared to optical spectroscopic methods.^[^ ^7^, ^8^ ^]^ This leads to extended times of data acquisition, the need for excessive sample amounts, and costly isotopic labeling of the investigated probes, thus hampering its broad application, e.g., in analyzing biological samples. The limited sensitivity arises from the small energy gap that separates the nuclear Zeeman spin states, leading to only a slight population imbalance (polarization) of spin states given by the Boltzmann distribution, even at low temperatures. Overcoming this handicap has been one of the main challenges in magnetic resonance research in the last decades.^[^ ^7^, ^9^, ^10^, ^11^, ^12^, ^13^, ^14^, ^15^ ^]^

**Figure 1:**
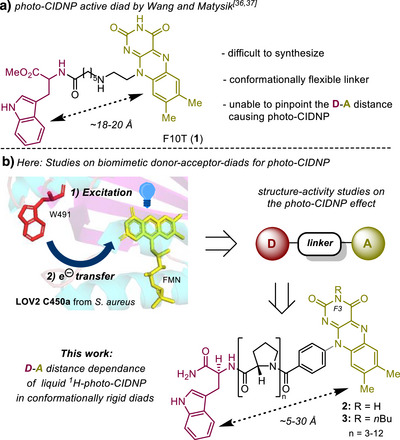
a) Small molecular F10T (**1**) ^1^H‐photo‐CIDNP diad investigated by Wang and Matysik.^[^ ^36^, ^37^ ^]^ b) Development of photo‐CIDNP active artificial diads that emulate the LOV2 C450A domain in *S. aureus*,^[^ ^38^ ^]^ and offer different but distinct spatial arrangements of D and A to investigate the optimal distance for the photo‐CIDNP effect.

Several methods have been developed to increase the nuclear spin polarization beyond its thermal equilibrium distribution, leading to nuclear hyperpolarization.^[^ ^14^ ^]^ The NMR signals are enhanced by orders of magnitudes, offering the techniques for analyzing complex biological probes, such as metabolites,^[^ ^8^, ^16^ ^]^ natural products,^[^ ^17^ ^]^ amino acids,^[^ ^18^ ^]^ and lipids.^[^ ^19^, ^20^ ^]^ Among the most efficient hyperpolarization strategies are dynamic nuclear polarization (DNP),^[^ ^7^, ^8^, ^14^ ^]^ *para‐*hydrogen induced polarization (PHIP),^[^ ^6^, ^21^ ^]^ and signal amplification by reversible exchange (SABRE).^[^ ^12^, ^13^, ^14^, ^15^, ^16^, ^17^, ^18^, ^19^, ^20^, ^21^, ^22^ ^]^ An alternative to these methods is *photoc* hemically *i* nduced *d* ynamic *n* uclear *p* olarization (photo‐CIDNP),^[^ ^23^, ^24^, ^25^ ^]^ as it generates hyperpolarization in a mild and biocompatible way, simply by illumination with visible light. This phenomenon has also been observed in proteins, such as photosynthetic reaction centers (RCs)^[^ ^23^, ^26^, ^27^ ^]^ and light‐oxygen‐voltage (LOV) photoreceptor domains (Figure 1b).^[^ ^28^, ^29^, ^30^ ^]^ Inspired by the natural systems, flavin compounds, such as flavin adenine dinucleotide (FAD) and flavin mononucleotide (FMN) have been investigated as dyes and electron acceptors (A) in liquid‐state photo‐CIDNP NMR.^[^ ^31^ ^]^ In combination with redox‐active amino acids, such as tyrosine (Tyr) or tryptophan (Trp), which act as electron donors (D), they create, upon irradiation with blue light, a transient spin‐correlated radical pair (SCRP).^[^ ^32^, ^33^ ^]^ Magnetic interactions occurring in the SCRP lead to the build‐up of nuclear hyperpolarization.

The first artificial diads showing a photo‐CIDNP effect were presented by Paddon‐Row et al.^[^ ^34^, ^35^ ^]^ More recently, Matysik et al. studied the F10T diad (**1**) that mimics the cryptochrome A‐D distance (center‐to‐center ∼18–20 Å).^[^ ^36^, ^37^ ^]^ The F10T (**1**) includes the flavin (A) connected to a tryptophan (D) by a C_7_ alkyl chain (Figure 1a). However, presumably because of its conformational flexibility, the distinct conformation of **1** and thus the optimal donor–acceptor distance responsible for photo‐CIDNP could not be determined. To fill this knowledge gap, rigid linker systems capable of dialing in a specific A‐D distance are needed to reveal the structure‐activity relationship for photo‐CIDNP in artificial diads. From a long‐term perspective, this will facilitate a rationally tailored and quasi‐biological diad design, fostering the application of both liquid‐state and solid‐state photo‐CIDNP as a tool in modern NMR and bio‐MRI.

In the presented work, we utilize a holistic approach combining synthesis, spectroscopy, and theoretical investigations to shed light on the structure‐photo‐CIDNP relationship in the liquid state. The benefits of small molecules, in contrast to proteins and biomolecules, include an easier tunability of the redox‐active components and better solvent compatibility, leading to systems that are easier to study and that offer broader applicability of the photo‐CIDNP effect in the future. Our study focuses on the ^1^H‐liquid‐state photo‐CIDNP effect depending on the distance of A and D. Therefore, the photo‐CIDNP effect in minimalistic diads **2** and **3** with different linker lengths and substitution patterns at the *F3*‐nitrogen in the flavin moiety has been investigated.

## Results and Discussion

Inspired by the active site in LOV domains,^[^ ^28^, ^29^, ^38^, ^39^ ^]^ we synthesized biomimetic diads **2** and **3** (see Figure 1b) employing a flavin moiety as an electron acceptor and tryptophan as an electron donor. Both entities were separated by an oligoproline linker of varying lengths composed of different numbers of proline (Pro) units. Besides the straightforward synthesis of polyprolines by solid‐support peptide synthesis (SSPS, see Supporting Information, chapter 3), they are known for their conformational rigidity (“molecular rulers”), which is why substituents at the *N*‐ and *C*‐termini can be allocated at precise distances by varying the peptide length.^[^ ^40^, ^41^, ^42^ ^]^ In protic solvents like water and methanol, oligoprolines adopt predominantly polyproline II (PPII) helical structures,^[^ ^40^, ^43^, ^44^, ^45^, ^46^, ^47^ ^]^ with three prolines building one turn, accounting for a rise of approximately 9 Å per turn.

**Scheme 1:**
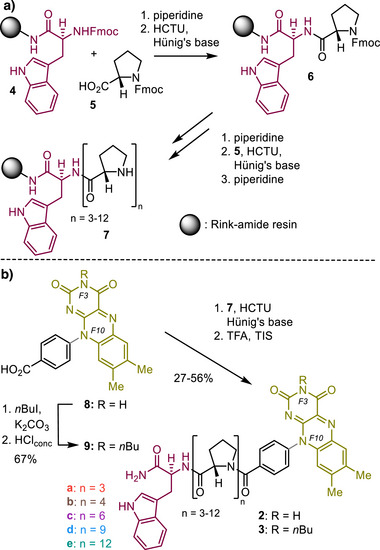
a) SPPS of the Trp‐Pro_n_ donor‐linker moiety **7**; i) piperidine; ii) HCTU, Hünig's base; b) Assembly of the diads **2** and **3** with different linker lengths (*n* = 3–12). i) **7**, HCTU, Hünig's base; ii) TFA, TIS, 27%–56% yield over two steps; iii) *n*BuI, K_2_CO_3_; iv) concentrated HCl, 25% yield over two steps; HCTU = *O*‐(1*H*‐6‐Chlorobenzotriazole‐1‐yl)‐1,1,3,3‐tetramethyluronium hexafluorophosphate; TFA = Trifluoroacetic acid; TIS = Triisopropylsilane.

The synthetic pathway was designed based on a modular principle to quickly and straightforwardly access structurally diverse diads **2** and **3** (Scheme 1). We started with the automated SPPS on Rink‐amide resin to generate Trp‐Pro peptides **7** with linker sizes varying from 3 to 12 Pro units (Scheme 1A and Supporting Information). In parallel, the flavin‐derivative **8** exhibiting an *F10*‐*p*‐benzoic acid moiety necessary to attach the flavin to the Pro‐linker **7** was synthesized by adapting a protocol by Inoue et al. (see Supporting Information).^[^ ^48^ ^]^ Flavin **8** was connected to the Trp‐Pro analogs **7** utilizing the peptide coupling reagent *O*‐(1*H*‐6‐chlorobenzotriazole‐1‐yl)‐1,1,3,3‐tetramethyluronium hexafluorophosphate (HCTU) and Hünig's base. Removing the products from the resin by acidic cleavage provided the diads **2** with linker lengths of Pro‐3 **2a**, Pro‐4 **2b**, Pro‐6 **2c**, Pro‐9 **2d**, and Pro‐12 **2e**. To impede intermolecular electron transfer due to the self‐assembly of **2** in solution by intermolecular hydrogen bonding, another set of diads **3** was synthesized, bearing an *n*‐butyl group at *F3* nitrogen. Therefore, flavin **8** was treated with *n*Bu under basic conditions followed by saponification of the likewise formed *n*Bu ester moiety. The obtained *N*‐*n*Bu flavin **9** was attached to the corresponding Trp‐Pro_n_ linker **7** using the above protocol.

The predominant secondary structure of the oligoproline bridge of **2** and **3** was determined by circular‐dichroism (CD) and NMR spectroscopy in deuterated methanol (MeOH‐*d*_4_, see Supporting Information, chapter 4 and 5.2). While cotton effects for both Pro‐3 diads **2a** and **3a** were not observed, the CD spectra became more and more characteristic for a PPII helical structure with a negative CD signal at ca. 210 nm and a positive cotton effect at approximately 230 nm upon adding further proline units to the linker.^[^ ^49^ ^]^ Superimposed 2D‐^1^H‐NOESY and TOCSY NMR spectra further corroborated that diads **2** and **3** mainly form a PPII helical motif with the amide bonds adopting the PPII typical all‐*trans* conformation (see Supporting Information, chapter 5.2). Nevertheless, other amide conformers were still visible but became less populated with increasing linker length (Pro‐4 52% PPII to Pro‐12 70% PPII helix). The helicity observed for the different diads is independent of the *F3* substituent.

**Figure 2:**
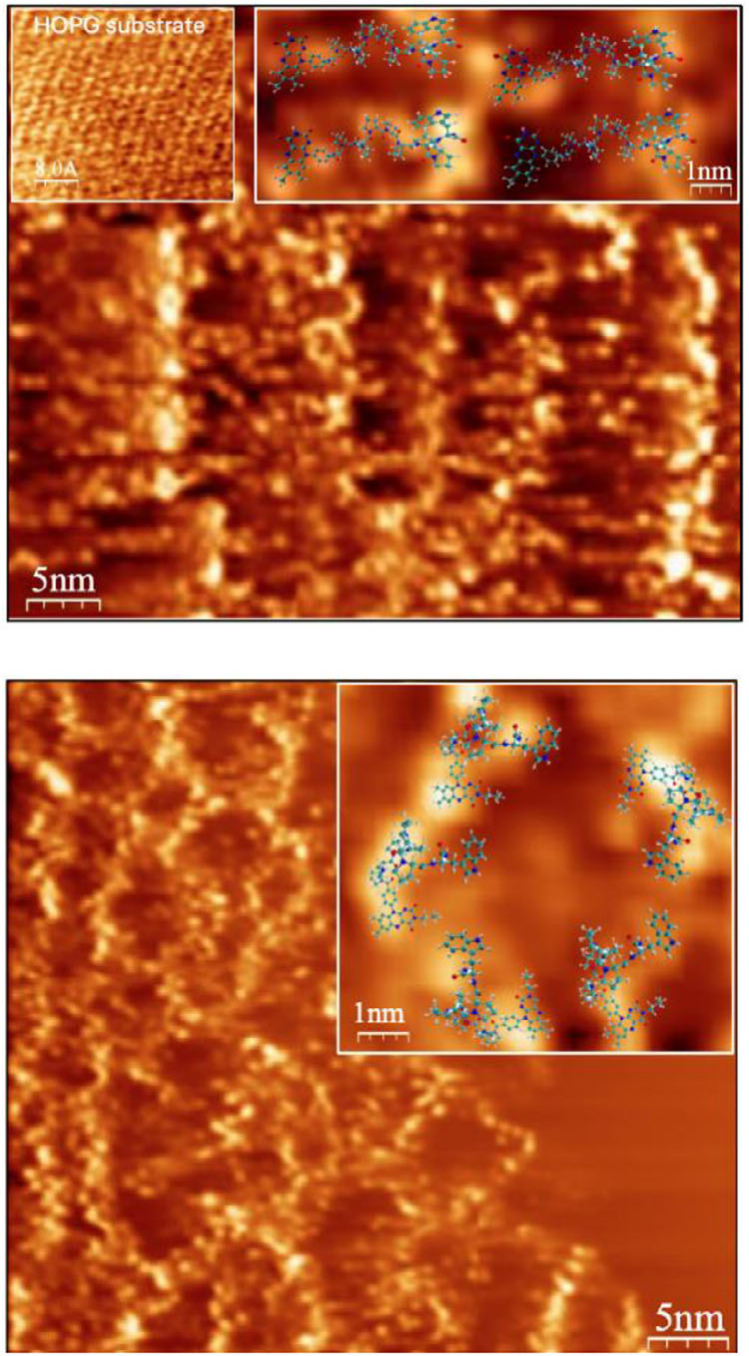
STM image of a) *N‐H* diad **2d** and b) *N*‐alkylated **3c** diad. A droplet of 1 µL of each diad in ethanol (1 mM) was deposited on the HOPG (0001) surface (shown in inset in a). All STM experiments were performed under ambient conditions at room temperature by using mechanically made Pt/Ir tips. The tunneling parameters were *U*_t_ = 1.5 V and *I*_t_ = 1.0 nA. The molecular rendering in the insets illustrates the configuration of some typical conformations of the diads, as obtained from MD simulations (see below).

Further structural characterization was done using scanning tunneling microscopy (STM) of diads deposited on a highly ordered pyrolytic graphite (HOPG) surface. This analysis was explicitly helpful for analyzing possible inter‐diad interactions. Figure 2a illustrates an STM image of the adsorbed *N‐H* diad **2d**, revealing predominantly linear molecular structures and showing a well‐ordered lamellar film with a size of about 6 ± 0.5 nm between individual diads and an overall periodicity of about 12 ± 0.5 nm corresponding to two diads. Here, the flavin and the Trp moieties of adjacent diads interact via hydrogen bonding. The *N*‐alkylated variants **3** form a ring‐like structure (see Figure 2b), with the characteristic size of each ring varying from 2.5 nm to 5.0 nm ± 0.2 nm. This observation hints at the disruption of the Trp‐flavin H‐bonding network compared to the compound **2** cases. The center‐to‐center distances between the Trp and flavin moieties as estimated from the STM images are approximately 3.0 ± 0.5 nm for **2d** and 2.5 ± 0.5 nm for **3c**, which are in qualitative agreement with the distances obtained from MD simulations (2.5 and 1.5 nm, respectively).

Molecular dynamics (MD) simulations (see Supporting Information for more information) were employed for diads **2** and **3** to identify which diad best resembles the biomimetic character regarding radical–radical distance and stability. Exemplary animations of the diad dynamics are found in the Supporting Information. To mimic the flavin^•‒^ and Trp^•+^‐containing SCRPs occurring in, e.g., LOV domain proteins,^[^ ^50^, ^51^ ^]^ where the 16 Å center‐to‐center separation between the radicals (∼11 Å edge‐to‐edge) was particularly successful in receiving nuclear hyperpolarization at high magnetic fields, a rigid SCRP‐distance in a similar regime is required. Shorter distances might not allow for sufficient spin evolution, while too long distances prevent electron‐electron interactions.

**Figure 3:**
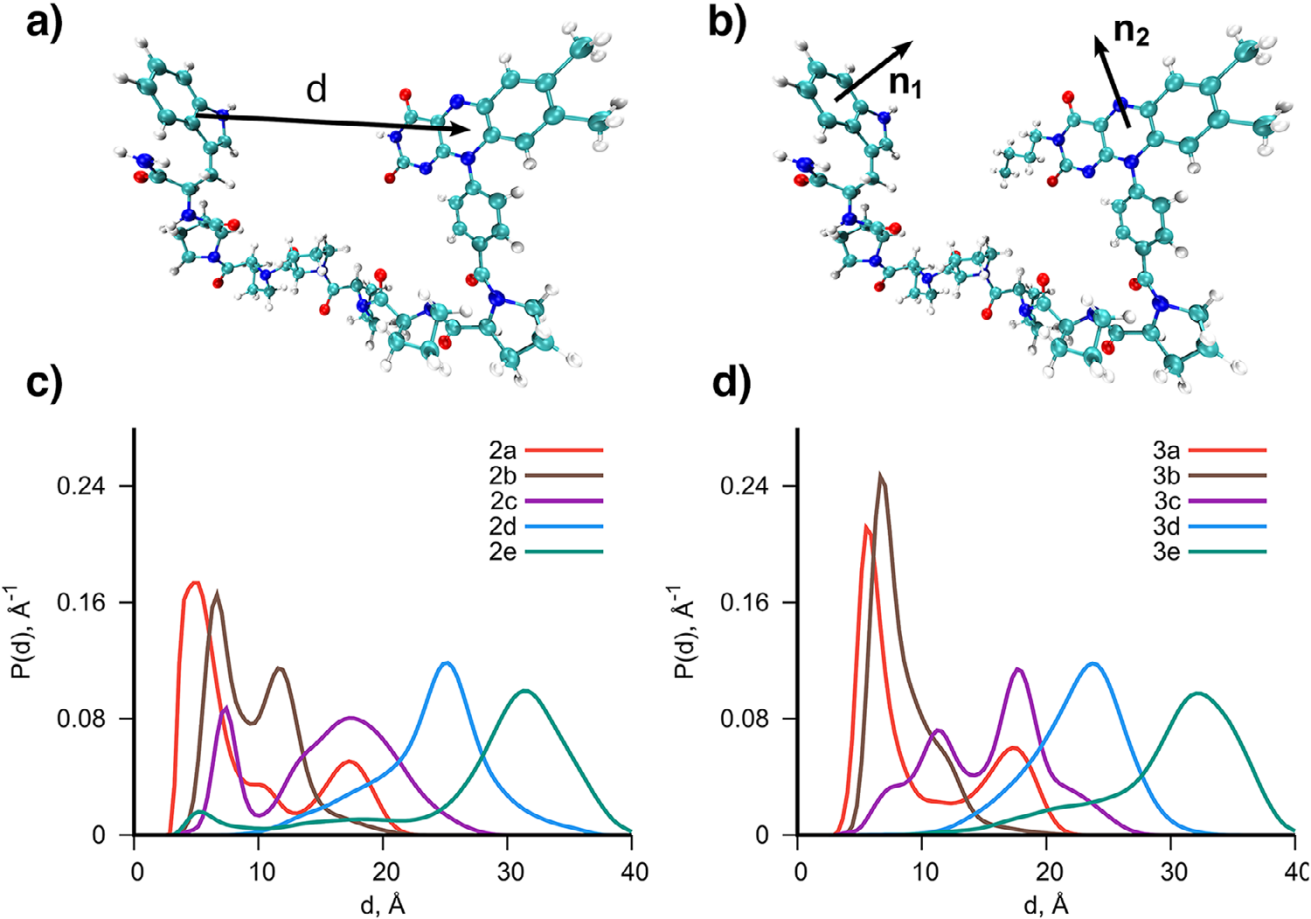
a) Visualization of the Pro‐6 *N‐H* diad **2c** and the vector d that defines the distance between the centers of the Trp and flavin moieties. b) Visualization of the Pro‐6‐*n*Bu diad **3b** with the normal vectors n_1_ and n_2_ defined perpendicular to the planes of the Trp and flavin moieties, used to obtain relative angles between the radicals (see, Figures S38–S43). c) Probability density describing the distributions of possible distances d obtained from MD simulations, see Figure S40, arising in the Pro‐n‐*N‐H* diads **2** with the different linker lengths. d) Similar to c), but computed for the Pro‐n‐*N*‐*n*Bu diads **3** with different linker lengths.

Figure 3a defines the distance vector d between the two radical centers of the SCRP. Another property to study the rigidity of the diads is the relative angle orientation between the two radical centers (see Figure 3b). A detailed discussion on the relative angles between the two radicals for each simulated diad is provided in the Supporting Information. Figure 3c shows the probability density distribution of the obtained distances d for the different *N‐H* diad **2** linker lengths. The shorter diads with three (**2a**, red) and four (**2b**, brown) proline units per linker feature sharp maxima peaking at <15 Å. This indicates an increased conformational stability. Diads, however, with longer linkers (Pro‐9 **2d** (blue) and Pro‐12 **2e** (green)) are characterized by broader distributions with peaks at 25 Å (**2d**) and 32 Å (**2e**), respectively, indicating less stability. The Pro‐6 diad **2c** reveals two maxima at 8 and 17 Å, respectively. The expected flavin‐Trp distances do not change significantly upon changing the *F3*‐nitrogen in **2** with a *n‐*butyl group in **3**. For the shorter linker systems **3a** and **3b**, the maxima become sharper compared to the *N‐H* systems, while the more extended linker systems with Pro‐9 **2d** and **3d** and with Pro‐12 **2e** and **3e** remain similar in both cases. The Pro‐6 *N*‐*n*Bu diad **3c** has a more pronounced peak at around 17 Å compared to its *N‐H* congener **2c**, where the intensity of the 8 and 17 Å peaks are similar (see Table S4). We, therefore, conclude that both Pro‐6 diads **2c** and **3c** are, in principle, perfectly suited as biomimetic diads because of the close alignment of the SCRP center‐to‐center distance to the LOV domain proteins.^[^ ^50^, ^51^ ^]^

Quantum chemical calculations using density functional theory (see Supporting Information, chapter 8) further revealed that the electronic structure of the diads are not significantly altered by introducing a *N*‐*n*Bu‐substituent at the *N3*‐position. In all simulated cases (see, Table S6), the HOMO–LUMO energy gap remained similar; thus, the redox potential of the diad differs only marginally. This conclusion is supported by experimental UV/Vis spectra (Figures S48 and S49), which show only minor differences in absorption intensity and a slight hypsochromic shift (∼3 nm, corresponding to ∼0.018 eV) in the second absorption maximum for *N‐H* versus *N‐n*Bu substituted diads. Together, these findings confirm that the *n*‐butyl substitution has minimal effect on the intrinsic electronic properties of the flavin chromophore, indicating that only the inter‐diad interactions will be altered due to the missing hydrogen‐bonding.

To study the suitability of the Pro‐6 diads further, quantum chemical calculations were performed and compared to the *Cr*LOV1 domain protein (see Supporting Information, chapter 8). A comparison of orbital overlaps between the Trp and flavin moiety reveals that no significant overlap between Trp and flavin orbitals, like the *Cr*LOV1, can be found, indicating that either the flexibility of the diad promotes the charge‐transfer (like other diadic systems)^[^ ^52^ ^]^ or electron‐hopping through the proline chain is happening.

**Figure 4:**
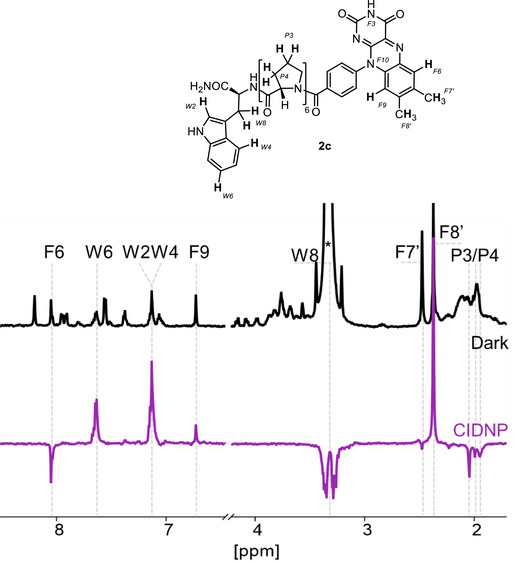
^1^H‐dark (black) and ^1^H‐photo‐CIDNP (purple) liquid‐state NMR spectra of the Pro‐6‐*N‐H* diad **2c** in MeOD‐*d*_4_ (0.2 mM). The resonance of the residual solvent is labeled with an asterisk. The sample was irradiated at 445 nm (0.6 W) for 0.5 s. Assignments of specific proton resonances (*F6*, *W6*, *W2*, *W4*, *F9*, *F7'*, *F8'*, *W8*, *P3*, and *P4*, shown in bold in **2c**) are indicated, highlighting hyperpolarization effects in the photo‐CIDNP spectrum.

Photo‐CIDNP liquid‐state NMR experiments with continuous sample irradiation successfully detected hyperpolarized ^1^H‐NMR signals for all synthesized diads **2** and **3** at a magnetic‐field strength of 14.1 T (corresponding to a ^1^H‐resonance frequency of 600 MHz) and a sample concentration of 0.2 and 0.1 mM, thus enabling the exploration of their spin dynamics (see Figures 4 and S32).^[^ ^53^ ^]^ MeOH*‐d*_4_ was selected as the solvent due to the good solubility of the diads and its ability to stabilize the PPII helix conformation in proline‐containing sequences.^[^ ^54^ ^]^ At the low concentration used in our experiments, the methanol‐diad solutions were transparent,^[^ ^55^ ^]^ allowing uniform irradiation of the sample (see Figure S31), resulting in a more pronounced and reproducible photo‐CIDNP‐NMR signal enhancement. Notably, the *F3*‐substituted diads **3** turned out to be more photostable under illumination with blue light than their *N‐H* analogs **2** (see Figure S34).

Photo‐CIDNP NMR experiments revealed that the enhanced signal pattern remains the same across all diads **2** and **3**, regardless of the number of proline units (see Figure S32). The degree of signal enhancement, however, varies depending on the linker length, as also illustrated by the relative enhancement factors summarized in Table S3 and Figure S33. The observed polarization signs^[^ ^56^ ^]^ are similar to the riboflavin–tryptophan system added separately to the solution.^[^ ^57^, ^58^ ^]^ Notably, the most substantial photo‐CIDNP enhancement is observed for the *F8’* methyl group. In contrast, the *F7’* protons are weakly polarized. Furthermore, two additional signals from the flavin moiety arising from the *F6* and *F9* protons with negative and positive enhancement, respectively, could be detected in the aromatic region of the spectrum (Figure 4). The recorded spectra also show signals from the polarized *W6*, *W2*, and *W4* protons from the aromatic fragment of the tryptophan and the aliphatic *W8* protons (details of signal assignments for these resonances are provided in the Table S1). Interestingly, ^1^H‐photo‐CIDNP hyperpolarization has been detected for the protons *P3* and *P4* of the oligoproline moiety, implying a potential involvement of the linker in the spin evolution processes.

**Figure 5:**
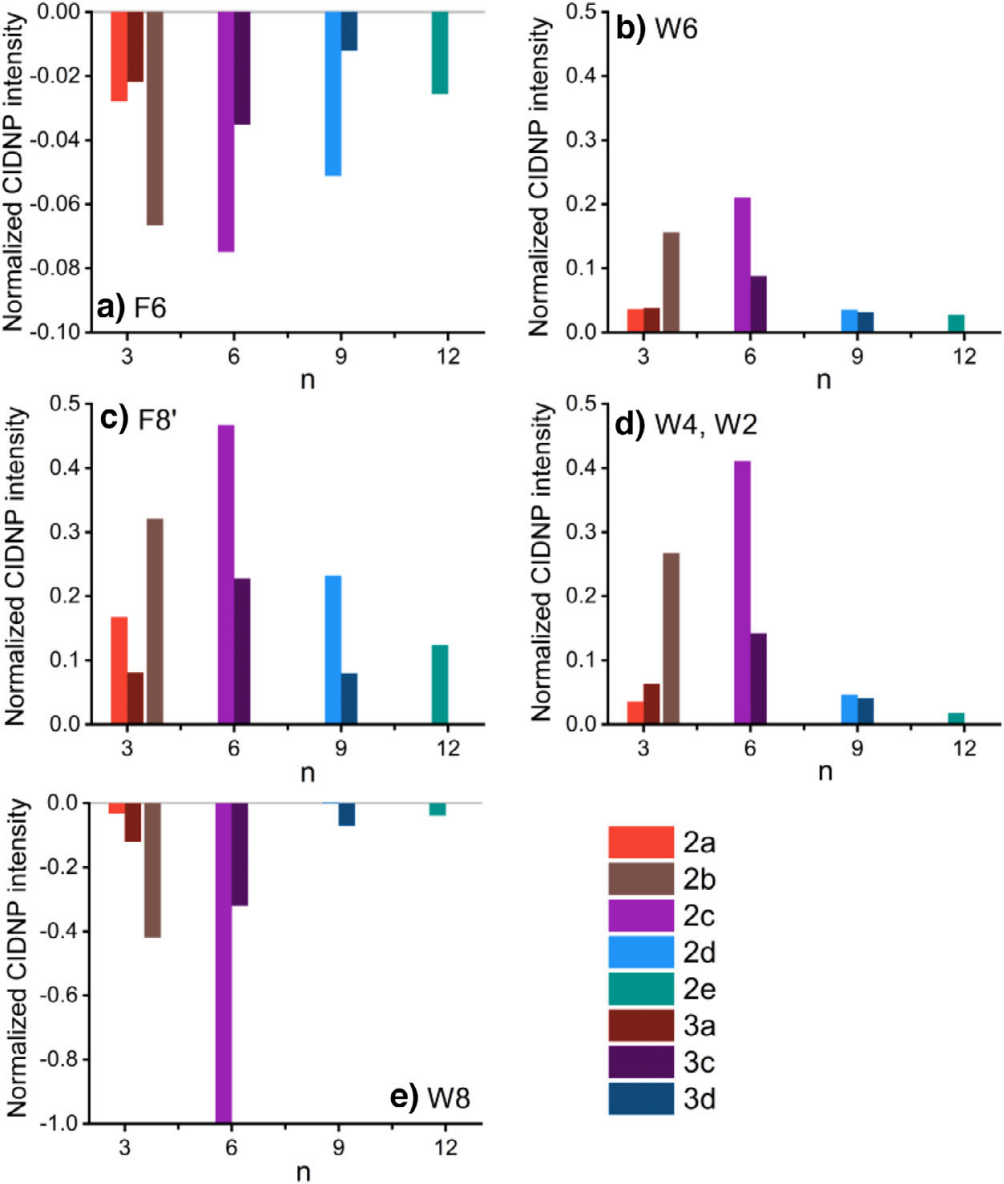
Normalized ^1^H‐photo‐CIDNP intensities for diads **2** and **3** with varying proline linker lengths *n* (*N‐H*: solid bars; *N*‐*n*Bu: solid bars with squares). Panels a)–e) correspond to different proton resonances displaying hyperpolarized photo‐CIDNP signals. The figure demonstrates the influence of the linker length and the *F3*‐substituent at the flavin in diads **2** and **3** on photo‐CIDNP intensities. *n* is the number of prolines in the linker. Signals of the corresponding peaks were integrated and normalized with the most intensive one set as 1.

Under our experimental conditions, the most significant hyperpolarization was observed for the diads **2c** and **3c**, containing six proline units in their linker (Figure 5). It is important to note that the *N‐n‐*butyl substituted diad **3c** shows lower signal intensities compared to its corresponding *N‐H* analogs **2c**. This trend is consistent within all diads. Still, our calculations indicate that the donor–acceptor distances do not differ significantly between the *N‐H* **2** and the respective *N‐n*Bu **3** diads (see Figure 3). Therefore, this phenomenon may arise from the build‐up of an intermolecular SCRP between flavin and Trp of two adjacent molecules in addition to the hyperpolarization caused by the intramolecular SCRP. This observation is in line with our STM investigations. Diads **2** displayed a dense hydrogen‐bonding network caused by intermolecular H‐bonding contacts of the flavin and Trp, which manifested in a laminar structure, as further supported by temperature‐dependent photo‐CIDNP measurements (see Figures S36 and S37), indicating enhanced signal intensity at elevated temperatures. In diads **3**, there are no noncovalent interactions between the *F3*‐nitrogen and the tryptophan of an adjacent diad possible, leading to a significantly reduced photo‐CIDNP effect for diads **3**. Nonetheless, a weaker but still notable temperature dependence of the photo‐CIDNP intensity was observed for **3d** (see Figure S37). This suppression of intermolecular radical pair formation in *n*‐butyl diads **3** makes them particularly well‐suited as models for LOV2 domains, where no intermolecular (i.e., interprotein) SCRP form under physiological conditions.

## Conclusion

The presented study exhibits a significant correlation between linker length, conformational rigidity, and the ^1^H‐photo‐CIDNP effect. CD and NMR spectroscopy and MD simulations show that the Pro‐3 and Pro‐4 linkers, such as **2a** and **3a**, are too short to stabilize a defined helical structure. Therefore, they exist as a mixture of different, highly flexible conformers showing *cis*‐ and *trans*‐amide configurations (e.g., for Pro‐4, ca. 50% all‐*trans* PPII helix is observed, cf. SI NOE TOCSY). The high content of peptides showing *cis*‐amide conformations leads to overall reduced D‐A distances in **2a/3a** and **2b/3b**. When the flavin and Trp are too closely positioned, the resulting radical pair recombines too rapidly for spin‐state evolution to occur. Consequently, no detectable CIDNP signal is generated. A sophisticated study on the SCRP lifetime for each diad will be presented in future work.

The Pro‐6 to Pro‐12 diads predominantly adopt a PPII helical conformation. This defined helical structure ensures a consistent and defined arrangement of flavin and Trp, creating favorable conditions for long‐lived SCRPs, essential for efficient photo‐CIDNP generation. With a distance peak at 17 Å, the Pro‐6 diads **2c** and **3c** closely mimic the spatial arrangement of photoactive proteins, such as the LOV domains (16 Å). This D‐A length optimum is likewise reflected in the photo‐CIDNP intensity most pronounced in **2c** and **3c** (Figure 5). Further increasing the D‐A distance, the electron exchange interaction decreases exponentially, facilitating more efficient *S‐T_0_* mixing within the SCRP. In diads with 9 and 12 proline residues, the D‐A distance exceeds 20 Å, larger than that in natural phototropin LOV domains.^[^ ^50^ ^]^ In general, the direct electron transfer efficiency decreases significantly beyond 10–15 Å (edge‐to‐edge),^[^ ^34^, ^59^ ^]^ making the process inefficient. However, recent studies have shown that in polyproline helical systems, the electron transfer rate does not decrease exponentially but declines much more gradually (β = 0.57 Å^−1^ instead of the expected 1.2–1.5 Å^−1^).^[^ ^60^ ^]^ This suggests that the PPII helix may moderate the adverse effect of increasing distance on charge transfer efficiency. Besides that, the flexibility of the diadic systems was demonstrated to promote electron transfer rates even if the average lengths exceed the expected lengths for efficient charge transfers.^[^ ^52^ ^]^ Interestingly, in our system, photo‐CIDNP polarization is observed not only on flavin and Trp but also on the *P3‐* and *P4‐*protons of the proline linker. Alternatively, this phenomenon could be explained by nuclear overhauser effect (NOE) enhancements because of the spatial proximity of the *P3‐* and *P4‐*protons to hyperpolarized protons in the flavin or Trp. This raises the question of whether proline actively participates in charge transfer or interacts with the SCRP, influencing spin dynamics. Addressing this question requires in‐depth experimental investigations, deciphering the detailed mechanism of the photo‐CIDNP effect in these systems, which is currently underway in our consortium.

Hence, we successfully synthesized and characterized a series of biomimetic flavin–tryptophan diads with varying linker lengths. The conformationally restricted oligoproline chain allowed for the first time to reveal significant insights into the structure‐photo‐CIDNP relationship. The optimal donor‐acceptor distance and conformational rigidity provided by the Pro‐6 linker in **2** and **3** mimic the natural photo‐CIDNP effect observed in LOV domain proteins. This work highlights the potential of these diads to enhance nuclear hyperpolarization in NMR spectroscopy, offering promising applications in bio‐NMR and MRI in the future.

## Supporting Information

The authors have cited additional references within the Supporting Information.^[^ ^51^, ^52^, ^54^, ^55^, ^61^, ^62^, ^63^, ^64^, ^65^, ^66^, ^67^, ^68^, ^69^, ^70^, ^71^, ^72^, ^73^, ^74^, ^75^, ^76^, ^77^, ^78^, ^79^, ^80^, ^81^, ^82^, ^83^, ^84^ ^]^

## Conflict of Interests

The authors declare no conflict of interest.

## Supporting information



Supporting Information

Supporting Information

Supporting Information

Supporting Information

Supporting Information

Supporting Information

Supporting Information

## Data Availability

The data that support the findings of this study are available in the supplementary material of this article.
