# Supplementary material for: Distance‐Dependence of Photo‐CIDNP in Biomimetic Tryptophan–Flavin Diads
Source: Angew Chem Int Ed Engl. 2025 Aug 5;64(38):e202510116. doi: 10.1002/anie.202510116 (PMC12435415; doi:10.1002/anie.202510116)
Supplement: Supplementary file 1 — Supporting Information [file ANIE-64-e202510116-s005.pdf]

# - SUPPORTING INFORMATION -

Distance-Dependence of photo-CIDNP in Biomimetic Tryptophan-Flavin Diads

Tobias Theiss,[a]+ Guzel Musabirova,[b]+ Luca Gerhards,[c] Irina S. Anisimova,[c] Ben G. E. Zoller,[d] Thi Ngoc Ha Nguyen,[e] Dmitry Denisov,[b] Anton Schmidt,[f] Sabrina Panter,[f] Stefan Weber,[f] Christoph Tegenkamp,[e] Ilia A. Solov'yov,[c,g,h]* Jörg Matysik,[b]* Tanja Gulder[a,d,i]*

[a] Department of Organic Chemistry, Leipzig University, Johannisallee 29, 04103 Leipzig, Germany

[b] Department of Analytical Chemistry, Leipzig University, Linnéstr. 3, 04103 Leipzig, Germany

[c] Institute of Physics, Carl-von-Ossietzky-University, Carl-von-Ossietzky-Str. 9-11, 26129 Oldenburg, Germany

[d] Organic Chemistry, Saarland University, 66123 Saarbrücken, Germany

[e] Department of Physics, Chemnitz University of Technology, Reichenhainer Str. 70, 09126 Chemnitz, Germany

[f] Institute of Physical Chemistry, Albert-Ludwigs-Universität Freiburg, Albertstr. 21, 79104 Freiburg, Germany

[g] Research Center Neurosensory Science, Carl-von-Ossietzky-Universität Oldenburg, 26111 Oldenburg, Germany

[h] Center for Nanoscale Dynamics (CENAD), Carl-von-Ossietzky-Universität Oldenburg, Ammerländer Heerstr. 114–118, 26129 Oldenburg, Germany

[i] Synthesis of Natural-Product Derived Drugs, Helmholtz Institute for Pharmaceutical Research Saarland (HIPS) Helmholtz Centre for Infection Research (HZI), 66123 Saarbrücken, Germany

+Both authors contributed equally

E-mail: tanja.gulder@uni-saarland.de

Content

1. General Information 3

2. Synthesis of F10-Flavin-Carboxylic Acid Derivatives **8** and **9** 4

 2.1 Preparation of Flavins **8** and **9** 4

 2.2 NMR Spectra of compounds **S2** – **S5**, **8** and **9** 8

3. Synthesis of CIDNP-diads **2** and **3** 13

 3.1 General protocol for automatic SPPS of the Pro_n-$TrpCONH_2$ building blocks 13

 3.1.1 Resin swelling 13

 3.1.2 Deprotection 14

 3.1.3 Peptide coupling 14

 3.1.4 Control of the Amide Coupling by LC-MS 14

 3.2 *N*-Terminal Capping of **7** with Flavin-*F*10-Carboxylic Acids **8** and **9** and Cleavage of the Diads **2** and **3** from the Resin 14

 3.3 Determination of Peptide Purity by LC-MS 15

 3.4 Analytical Data of Diads **2** and **3** 15

4. Circular Dichroism (CD) Spectra of Diads **2** and **3** 24

5. NMR-Analysis of Diads **2** and **3** 26

 5.1 NMR-Assignment of Diad **2e** 26

 5.2 Conformational Analysis of **2** and **3** by 2D-TOCSY and 2D-NOESY/ROESY-NMR Spectroscopy 28

 5.3 ^{1}H-NMR Spectra of Diads **2** and **3** 44

6. Experimental methods for ^{1}H-photo-CIDNP measurements 52

 6.1 Materials and Methods 52

 6.1.1 Sample Preparation 52

 6.1.2 NMR Spectroscopy 52

 6.1.3 ^{1}H-Photo-CIDNP Measurements 52

 6.2 ^{1}H-Photo-CIDNP Measurement Setup and Spectra of Diads **2** and **3** 53

 6.3 Photochemical Stability of Diad **2c** 56

 6.4 Temperature Dependent Experiment of Diads **2d** and **3d** 58

7. Molecular dynamics simulations 60

8. Quantum Chemical Calculations 70

9. Scanning Tunneling Microscopy (STM) Imaging 74

10. UV-vis spectroscopy 76

11. References 78

1. General Information

Solvents used in reactions were p.A. grade. Solvents for chromatography were technical grade and distilled before use. Anhydrous dichloromethane, *N*,*N*-dimethylformamide, and toluene were obtained from an MBraun MB-SPS 800 solvent purification system. Other dry solvents were purchased from Fluka and Acros in the highest purity and used without further purification. Reagents were purchased at the highest commercial quality and used without further purification. Yields refer to chromatographically and spectroscopically (^{1}H NMR) homogeneous materials unless otherwise stated. Reactions were monitored by thin layer chromatography (TLC) carried out on Merck silica gel aluminum plates with F-254 indicator using UV light as the visualizing agent (UV), basic potassium permanganate solution ($KMnO_4$), ceric ammonium molybdate (CAM), and heat as developing agents. Silica gel Merck 60 (particle size 40 – 60 µm) was used for flash column chromatography. Solvent mixtures are understood as volume/volume (v/v). NMR spectra were recorded on a Bruker-Spectrospin 600 MHz UltraShield NMR spectrometer, a Varian MERCURYplus 400 MHz spectrometer, and a Bruker Avance Neo 500 (500 MHz). The spectra were calibrated using residual undeuterated solvent as an internal reference ($CDCl_3$ @ 7.26 ppm, $CDCl_3$ @ 77.16 ppm ^{13}C NMR). The following abbreviations (or combinations thereof) were used to explain the multiplicities: s = singlet, d = doublet, dd = doublet of doublets, t = triplet, dt = doublet of triplets, q = quartet, m = multiplet, br = broad. In addition, the following abbreviations were used: DMF = *N*,*N*-dimethylformamide, EtOAc = ethyl acetate, MeCN = acetonitrile, DCM = dichloromethane, THF = tetrahydrofuran, TLC = thin layer chromatography, rt = room temperature, sat = saturated. Melting points were measured on a Büchi 510 and were not corrected. IR spectra were recorded on a JASCO FT-IR-4100 (ATR, film, or KBr) and are reported in terms of absorption frequencies. Mass spectrometry (MS) was conducted on a Thermo Scientific LTQ-FT ultra and ThermoFisher Scientific LTQ Orbitrap XL spectrometer (ESI HRMS). Unless otherwise stated, automatic solid phase peptide synthesis (SPPS) has been carried out on a Liberty Blue peptide synthesizer from CEM. Manual peptide synthesis was performed in 20 mL syringe reactors from Roth GmbH. Analytical high-performance liquid chromatography (LC)/MS data were obtained by an Agilent 1260 Infinity II device with a coupled InfinityLabLC/MSD Series mass spectrometer using an Agilent Poroshell 120 EC-C18 (2.1 x 50 mm, 2.7 µm) column. MeCN+0.1% formic acid (Solvent A) and water+0.1% formic acid (Solvent B) served as the mobile phase. For semi-preparative high-performance LC purification, an Agilent 1260 Infinity II device and a ReproSil Gold 120 C18 (250 x 10 mm) column (Dr. Maisch GmbH) was used. The mobile phase consisted of MeCN+0.1% TFA (Solvent A) and water+0.1% TFA (Solvent B).

2. Synthesis of F10-Flavin-Carboxylic Acid Derivatives **8** and **9**

2.1 Preparation of Flavins **8** and **9**

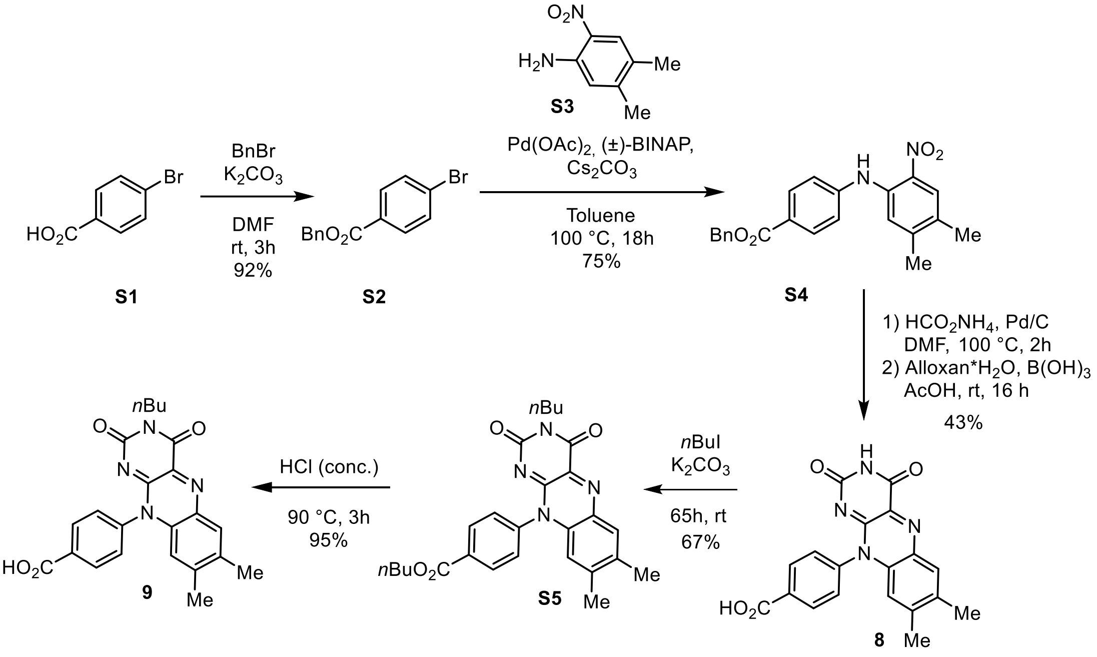

Scheme S1: Synthesis of the flavin-carboxylic acid derivatives **8** and **9**.[1]

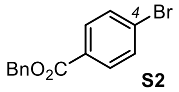

S2

4-Bromobenzoic acid benzyl ester (**S2**)**:** In a flame-dried 100 mL Schlenk flask, 4-bromo benzoic acid (**S1**, 6.00 g, 23.5 mmol, 1.00 eq.) and K_2CO_3 (3.57 g, 25.8 mmol, 1.10 eq.) were dissolved in 46 mL dry DMF under an Ar atmosphere. Benzyl bromide (4.02 g, 2.79 mL, 23.5 mmol 1.00 eq.) was added to the reaction mixture over 5 min at rt, and the reaction mixture was stirred for 4.5 h at ambient temperature. The solvent was removed under reduced pressure, the residue dissolved in 200 mL water and extracted with Et_2O (1x 300 mL, 1x 100 mL) and 300 mL EtOAc. The combined organic layers were washed with 100 mL water and 100 mL saturated NaCl solution, dried over $MgSO_4$, and the solvent removed under reduced pressure. Product **S2** was obtained as a crystalline white solid (7.23 g, 21.4 mmol, 92 %); **R$_f$** = 0.84 (silica gel, *n*-hexane/EtOAc 85:15); **m.p.** = 60 °C (CH_2Cl_2); lit.[2]: m.p. = +57 °C (CH_2Cl_2); **^{1}H NMR** (400 MHz, $CDCl_3$) δ 7.78 (m, 4H), 7.40 (m, 5H), 5.35 (s, 2H) ppm.

The physical and spectroscopic data are in accordance with those reported in the literature.[2]

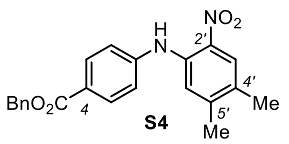

4-((4',5'-Dimethyl-2'-nitrophenyl)amino)benzoic acid benzyl ester (**S4**)**:** In a flame-dried 100 mL three-neck flask, 2-nitro-4,5-xylidine (**S3**, 1.29 g, 7.74 mmol, 1.00 eq.) and **S2** (3.97 g, 7.74 mmol, 1.00 eq.) was solved in 30 mL dry and degassed toluene under Ar-atmosphere. Subsequently, $Pd_2(dba)_3$ (354 mg, 0.387 mmol, 0.05 eq.), racemic 2,2'-bis(diphenylphosphino)-1,1'-binaphthyl (386 mg, 0.619 mmol, 0.08 eq.) and Cs_2CO_3 (3.53 g, 10.8 mmol, 1.40 eq.) was added and the mixture was stirred for 24 h at 110 °C. The reaction mixture was filtered over Celite®, mixed with 50 mL of EtOAc, and washed with 70 mL water and 70 mL saturated NaCl solution. The aqueous phase was extracted with ethyl acetate (2x 50 mL), the organic phase was dried over Na_2SO_4, and the solvent was removed under reduced pressure. The crude product was purified by column chromatography (SiO_2, *n*-hexane/EtOAc 90:10) giving **S4** as an orange solid (2.20 g, 5.84 mmol, 75%); $\mathbf{R_f}$ = 0.54 (SiO_2, *n*-hexane/EtOAc 90:10); **m.p.** = 79 °C (EtOAc); **IR** (ATR) $\tilde{\nu}_{max}$ 3326, 1710, 1512, 1497, 1450, 822 cm^{-1}; ^{1}**H NMR** (400 MHz, $CDCl_3$) δ 9.39 (br s, 1H NH), 8.08 (d, *J* = 13.7 Hz, 2H, H_{Ar}), 7.98 (s, 1H, H_{Ar}), 7.47 (m, 5H, H_{Ar}),7.28 (d, 2H, *J* could not be determined due to overlap with $CDCl_3$, H_{Ar}) 7.25 (s, 1H, H_{Ar}), 5.37 (s, 2H, CH_2), 2.24 (s, 6H, CH_3) ppm; ^{13}**C NMR** (101 MHz, $CDCl_3$) δ 166.3, 166.0, 146.7, 144.6, 144.4, 138.6, 136.2, 136.1, 133.1, 131.6, 130.5, 129.8, 128.9, 128.8, 128.8, 128.5, 128.4, 128.3, 127.4, 126.7, 125.2, 120.8, 117.7, 66.97, 66.77, 20.66, 18.99; two rotamers could be observed; **HRMS** (ESI^+) calcd. for $C_{22}H_{21}N_2O_4$ $[M+H]^+$ 377.1496, found 377.1509.

The physical and spectroscopic data are in accordance with those reported in the literature.[1]

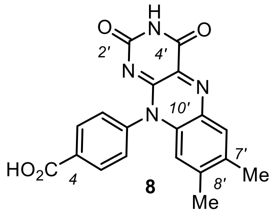

4-(7',8'-Dimethyl-2',4'-dioxo-3',4'-dihydrobenzo[g]pteridin-10'(2*H*)-yl)benzoic acid (**8**)**:** In a flame-dried 250 mL Schlenk-flask, pre-dried ammonium formate (3.40 g, 53.9 mmol, 5.20 eq.), **S3** (3.90 g, 10.4 mmol, 1.00 eq.) and Pd/C (10%)(1.10 g, 0.10 eq.) were suspended in 20 mL dry DMF. The mixture was heated to 100 °C until bubbling occurred (Attention: High pressure!) and stirred for 3h. The almost transparent suspension was filtered over Celite, washed with DMF and the solvent was removed under reduced pressure (50°C, 15 mbar, 1h). Then water was added, and the crude oil was extracted with EtOAc (3x 100mL), washed with brine, and dried over $MgSO_4$ to obtain 1.39 g of a brown/purple solid after evaporating the solvent.

Then, the crude amine was dissolved in 50 mL glacial acetic acid and degassed by bubbling argon through the solution. Under a constant argon stream, alloxan monohydrate (3.46 g, 21.6 mmol, 4.00 eq.) and boric acid (6.68 g, 108 mmol, 8.00 eq.) were added and the mixture was stirred for 7 h at rt. The deep orange suspension was filtered affording an orange solid, which was washed with 100 mL

water. An aqueous 3 wt-% NaOH solution was added until the solid was entirely resolved. Immediately, a 3M HCl solution was added dropwise until no more precipitation of an orange solid was detectable. The orange solid was filtered, washed thoroughly with water, and dried by lyophilization to yield compound **8** (1.61 g, 4.45 mmol, 43% over two steps); **m.p.** = 221 °C (H_2O, decomp.); **IR (film)** $\tilde{\nu}_{max}$ 3442, 1713, 1620, 1577, 1540, 1279, 778, 764 cm^{-1}; **^{1}H NMR** (400 MHz, DMSO-*d6*) δ 11.33 (s, 1H, NH), 8.25 (d, *J* = 8.5 Hz, 2H, H_{Ar}), 7.97 (s, 1H, H_{Ar}), 7.56 (d, *J* = 8.4 Hz, 2H, H_{Ar}), 6.57 (s, 1H, H_{Ar}), 2.38 (s, 3H, CH_3), 2.28 (s, 3H, CH_3) ppm; **^{13}C NMR** (101 MHz, DMSO-*d6*) δ 166.6, 159.7, 155.4, 151.4, 146.3, 139.8, 138.0, 135.9, 133.6, 132.1, 131.9, 131.3, 130.8, 128.3, 116.4, 20.45, 18.81 ppm; **HRMS** (ESI+) calcd. for $C_{19}H_{15}N_4O_4$ $[M+H]^+$ 363.1088, found 363.1088.

The physical and spectroscopic data are in accordance with those reported in the literature.[1]

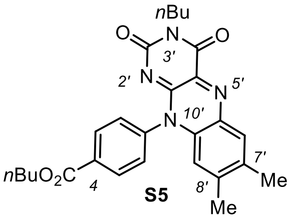

4-(3'-*N*-*n*-Butyl-7',8'-dimethyl-2',4'-dioxo-3',4'-dihydrobenzo[g]pteridin-10'(2*H*)-yl)benzoic acid *n*-butyl ester (S5): In a 50 mL round bottom flask, **8** (500 mg, 1.38 mmol, 1.00 eq.) was suspended in 30 mL DMF (0.05 M), and K_2CO_3 (1.53 g, 11.0 mmol, 8.00 eq.) was added. The mixture was degassed by bubbling argon through the solution. After 15 min stirring at rt, 1-iodo-*n*-butane (1.25 mL, 11.0 mmol, 8.00 eq.) was added over 10 min. The reaction mixture was stirred at room temperature until LC-MS confirmed the full conversion of the starting material (65 h). Sat. NH_4Cl solution was added to the reaction mixture. The precipitate was extracted with DCM (3x 50mL), the combined organic phases were dried over Na_2SO_4, and the solvent was removed under reduced pressure. The crude product was purified by column chromatography (SiO_2, DCM to DCM/MeOH 97:3) to yield **S5** as an orange solid (435 mg, 0.92 mmol, 67%); **R_f** = 0.50 (SiO_2, DCM/MeOH 95:5); **m.p.** = 230 °C (DCM, decomp.);); **IR** (ATR) $\tilde{\nu}_{max}$ 2956, 2933, 1721, 1706, 1651, 1538, 1271, 1255, 1097, 769, 757 cm^{-1}; **^{1}H NMR** (500 MHz, $CDCl_3$) δ 8.34 (m, 2H, H_{Ar}), 8.07 (s, 1H, H_{Ar}), 7.39 (m, 2H, H_{Ar}), 6.58 (s, 1H, H_{Ar}), 4.41 (t, *J* = 6.6 Hz, 2H, CH_2), 4.06 (t, *J* = 7.5 Hz, 2H, CH_2), 2.41 (s, 3H, CH_3), 2.32 (s, 3H, CH_3), 1.80 (m, 2H, CH_2), 1.68 (m, 2H, CH_2), 1.51 (m, 2H, CH_2), 1.41 (m, 2H, CH_2), 1.01 (t, *J* = 7.4 Hz, 3H, CH_3), 0.94 (t, *J* = 7.3 Hz, 3H, CH_3) ppm; **^{13}C NMR** (126 MHz, $CDCl_3$) δ 165.5, 159.6, 155.3, 149.7, 147.6, 139.0, 136.9, 136.7, 134.6, 132.6, 132.3, 132.2, 132.0, 128.0, 116.7, 65.61, 41.95, 30.87, 29.95, 21.36, 20.32, 19.64, 19.38, 13.97, 13.89 ppm; **HRMS** (ESI^+) calcd. for $C_{27}H_{31}N_4O_4$ $[M+H]^+$ 475.2340, found 475.2347.

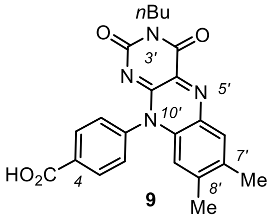

9

4-(3'-*N*-*n*-Butyl-7',8'-dimethyl-2',4'-dioxo-3',4'-dihydrobenzo-[g]pteridin-10(2*H*)-yl) benzoic acid (9): In a 100 ml round bottom flask, **S5** (132 mg, 0.28 mmol) was charged with 7 mL concentrated HCl. The reaction was stirred for 3 h at 90 °C. The mixture was cooled to rt and water was added until no precipitation of an orange solid was visible. The resulting suspension was transferred in a 50 mL falcon tube and centrifuged at 9000 rpm at 4 °C. The solid was washed with water (2x 25 mL) and subsequently centrifuged again. The orange solid was dried by lyophilization, after which 95.0 mg of product **9** (0.23 mmol, 95%) was isolated. **m.p.** = 315 °C (H_2O, decomp); **IR** (ATR) $\tilde{\nu}_{max}$ = 2960, 2933, 1715, 1699, 1578, 1539, 1250, 1020, 764, 757 cm^{-1}; **^{1}H NMR** (300 MHz, DMSO-*d*$_6$) δ 8.26 (d, *J* = 8.4 Hz, 2H, H_{Ar}), 8.02 (s, 1H, H_{Ar}), 7.56 (d, *J* = 8.3 Hz, 2H, H_{Ar}), 6.90 (s, 1H, H_{Ar}), 3.85 (t, 1H, CH_2), 2.39 (s, 3H, CH_3), 2.29 (s, 3H, CH_3), 1.52 (q, *J* = 7.6 Hz, 2H, CH_2), 1.31 (q, *J* = 7.4 Hz, 2H, CH_2), 0.90 (t, *J* = 7.3 Hz, 3H, CH_3) ppm; **^{13}C NMR** (126 MHz, DMSO-*d*$_6$) δ 166.6, 159.2, 154.6, 150.0, 146.5, 139.6, 137.2, 136.0, 133.9, 132.2, 131.9, 131.3, 130.8, 128.4 (2C), 116.3, 29.48, 20.47, 19.69, 18.84, 13.75. ppm; **HRMS** (ESI$^+$) calcd. for $C_{23}H_{23}N_4O_4$ $[M+H]^+$ 419.1714, found 419.1724.

2.2 NMR Spectra of compounds **S2** – **S5**, **8** and **9**

1H-NMR spectrum recorded at 400 MHz

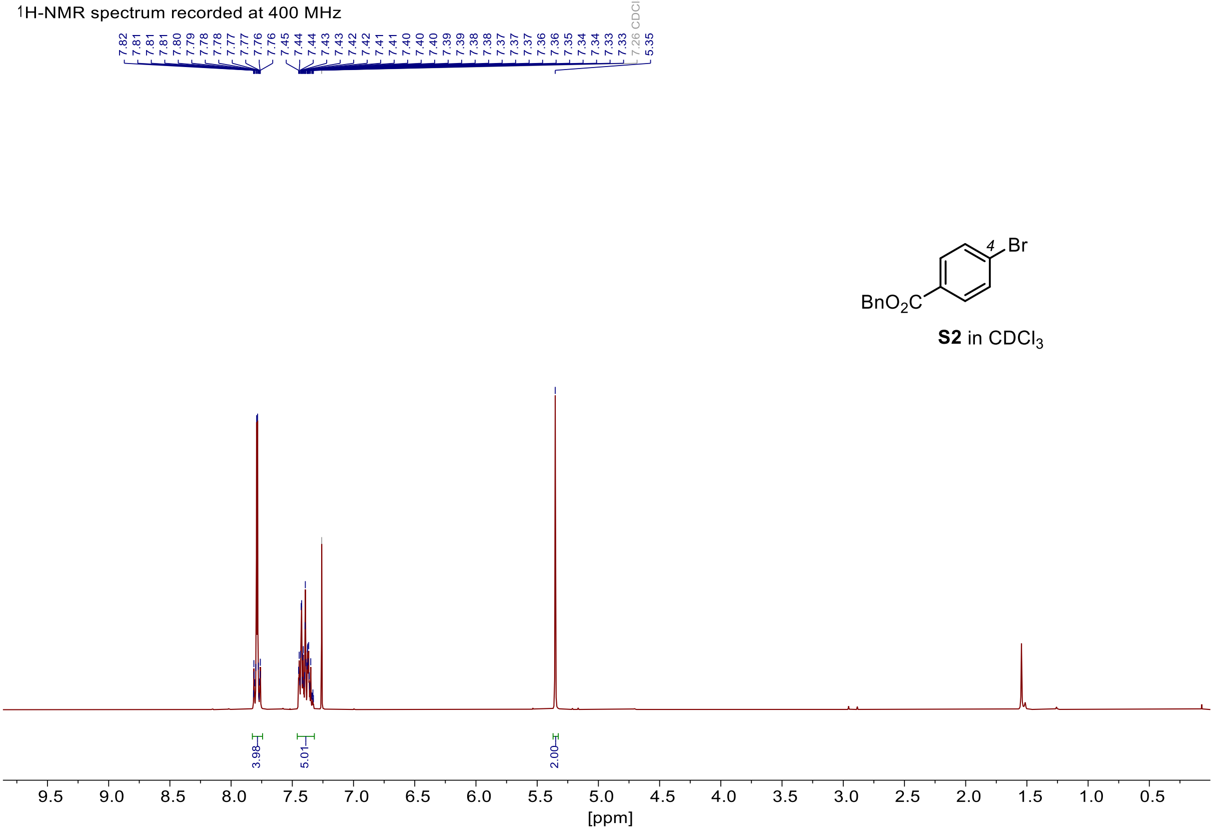

13C-NMR spectrum recorded at 101 MHz

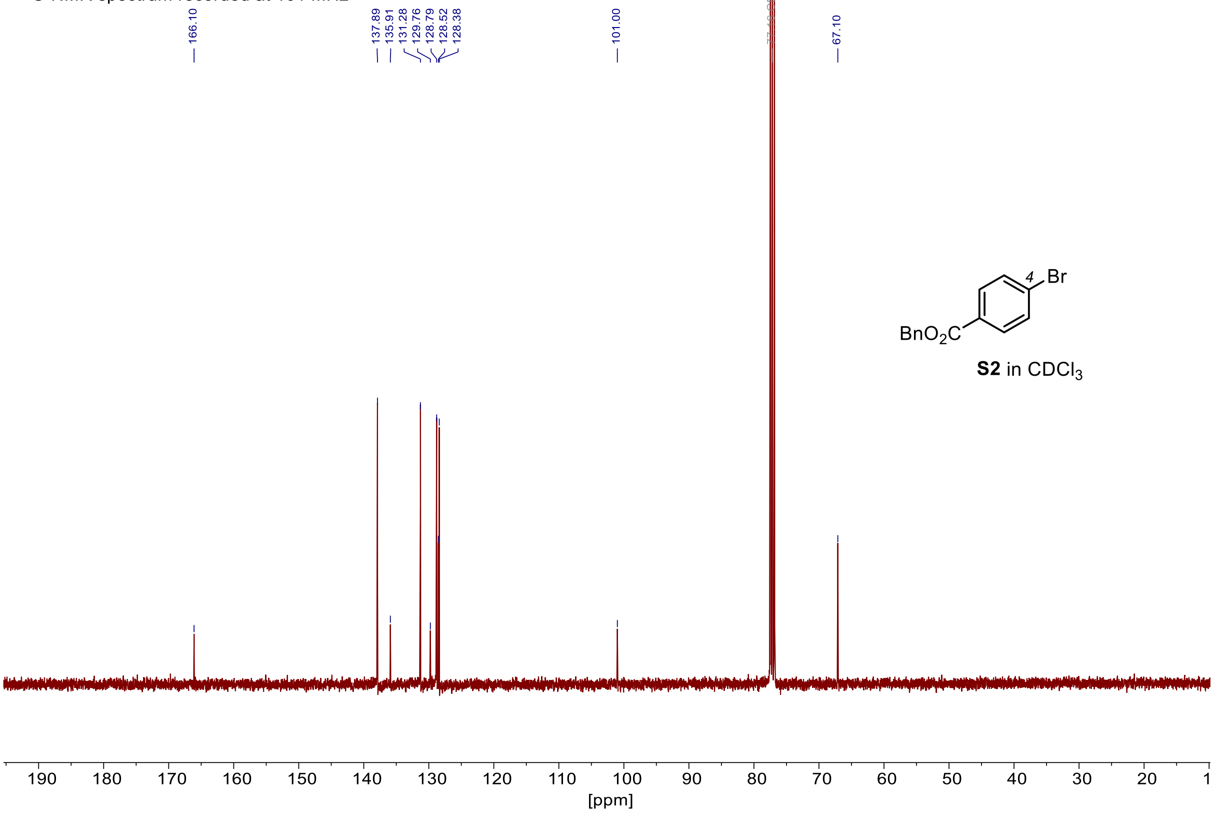

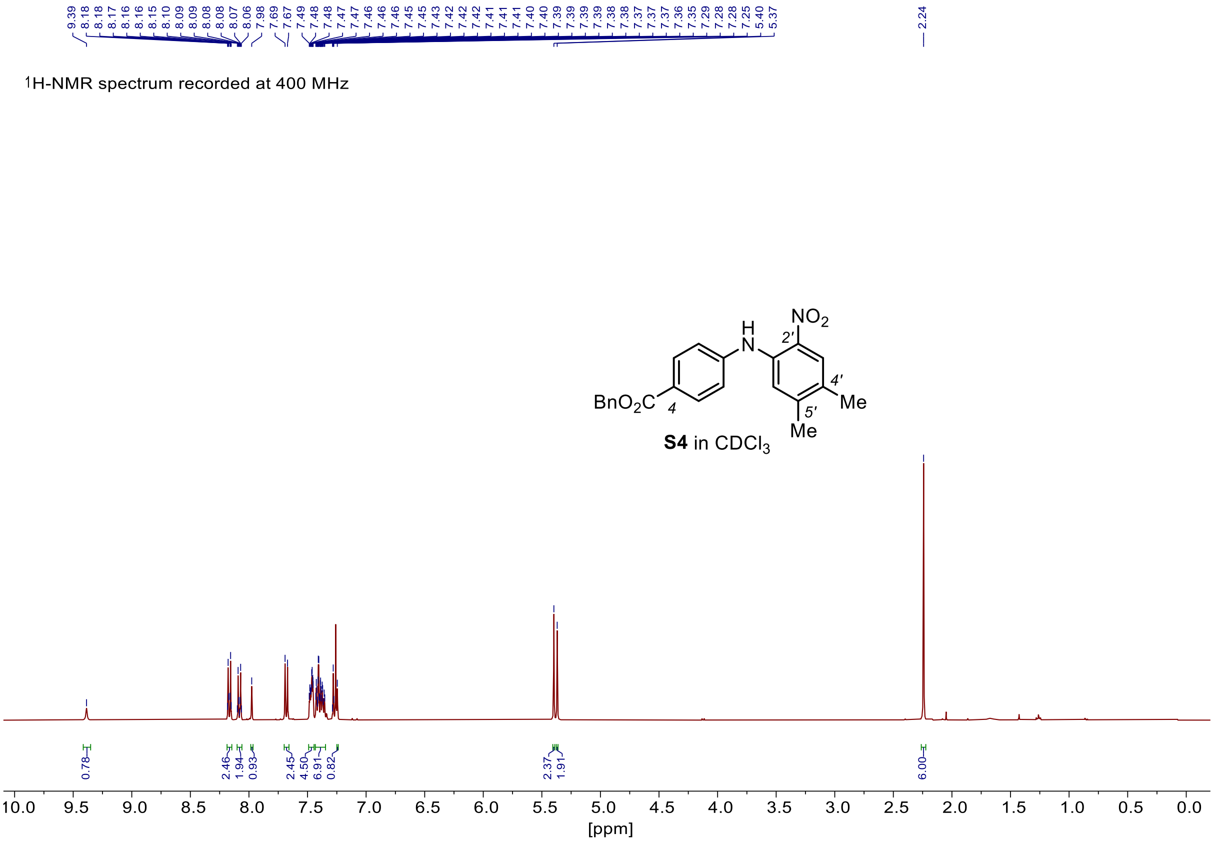
9.39 8.18 8.18 8.17 8.16 8.16 8.15 8.10 8.09 8.09 8.08 8.08 8.07 8.06 7.98 7.69 7.67 7.49 7.48 7.48 7.47 7.47 7.46 7.46 7.45 7.45 7.43 7.42 7.42 7.42 7.41 7.41 7.41 7.40 7.40 7.39 7.39 7.39 7.38 7.38 7.37 7.37 7.37 7.36 7.35 7.29 7.28 7.28 7.25 5.40 5.37
2.24
1H-NMR spectrum recorded at 400 MHz
H
N
NO2
2′
4′
Me
BnO2C
4
5′
Me
S4 in CDCl3
0.78 2.46 1.94 0.93 2.45 4.50 6.91 0.82 2.37 1.91 6.00
10.0 9.5 9.0 8.5 8.0 7.5 7.0 6.5 6.0 5.5 5.0 4.5 4.0 3.5 3.0 2.5 2.0 1.5 1.0 0.5 0.0
[ppm]

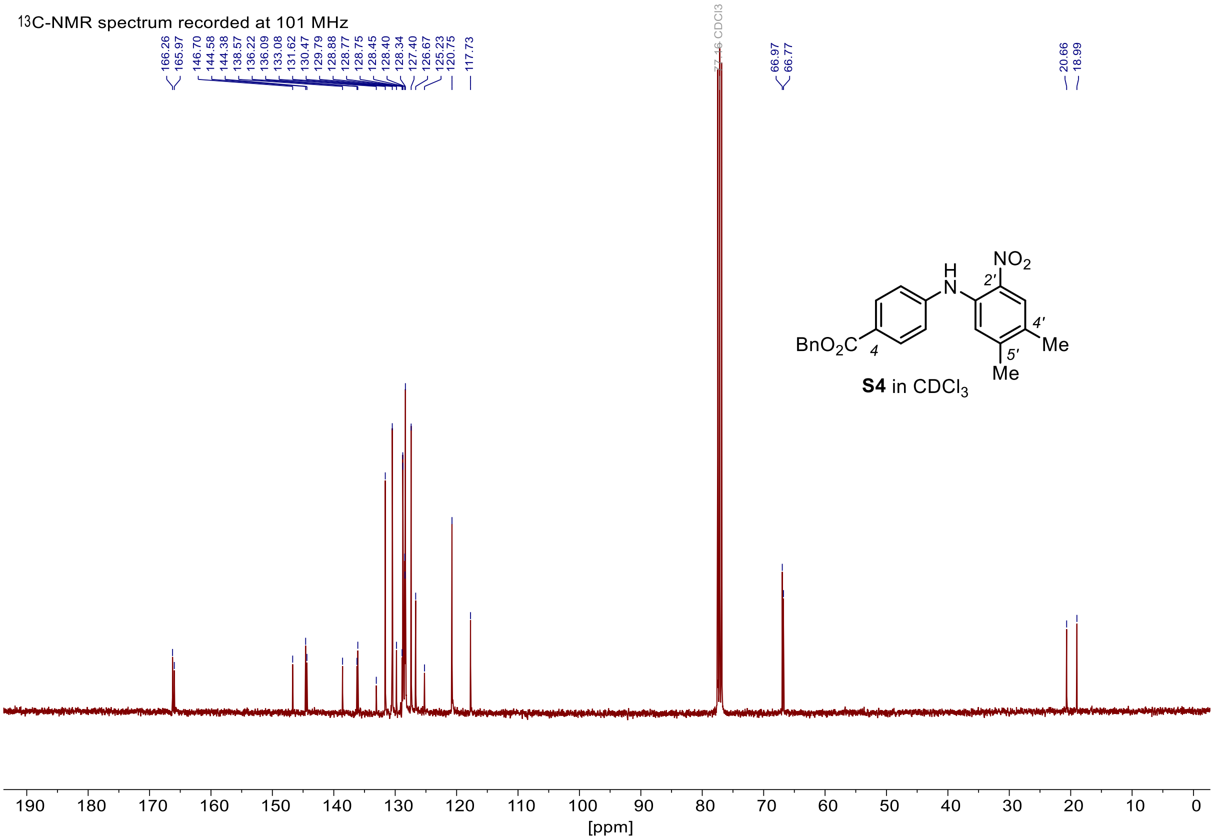
13C-NMR spectrum recorded at 101 MHz
166.26 165.97 146.70 144.58 144.38 138.57 136.22 136.09 133.08 131.62 130.47 129.79 128.88 128.77 128.75 128.45 128.40 128.34 127.40 126.67 125.23 120.75 117.73
CDCl3
66.97 66.77
20.66 18.99
H
N
NO2
2′
4′
Me
BnO2C
4
5′
Me
S4 in CDCl3
190 180 170 160 150 140 130 120 110 100 90 80 70 60 50 40 30 20 10 0
[ppm]

¹H-NMR spectrum recorded at 400 MHz

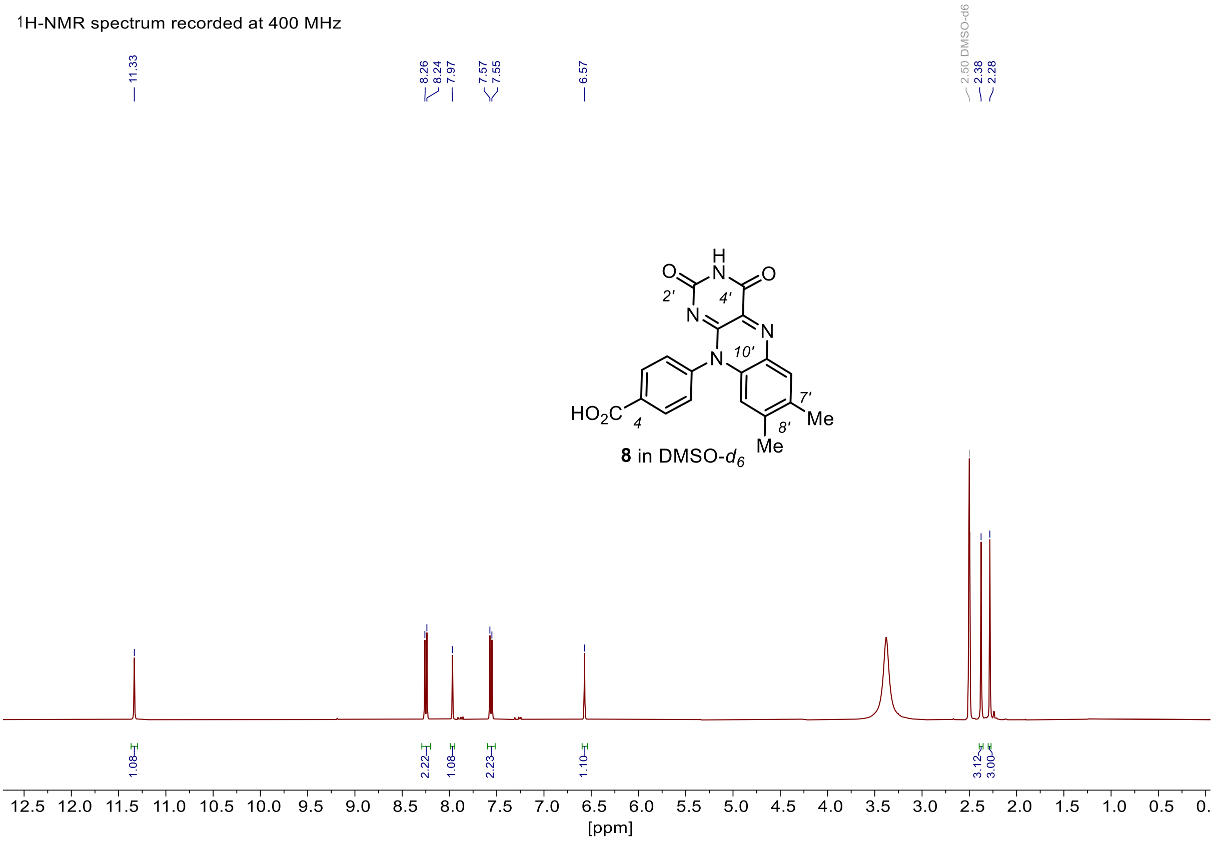

¹³C-NMR spectrum recorded at 101 MHz

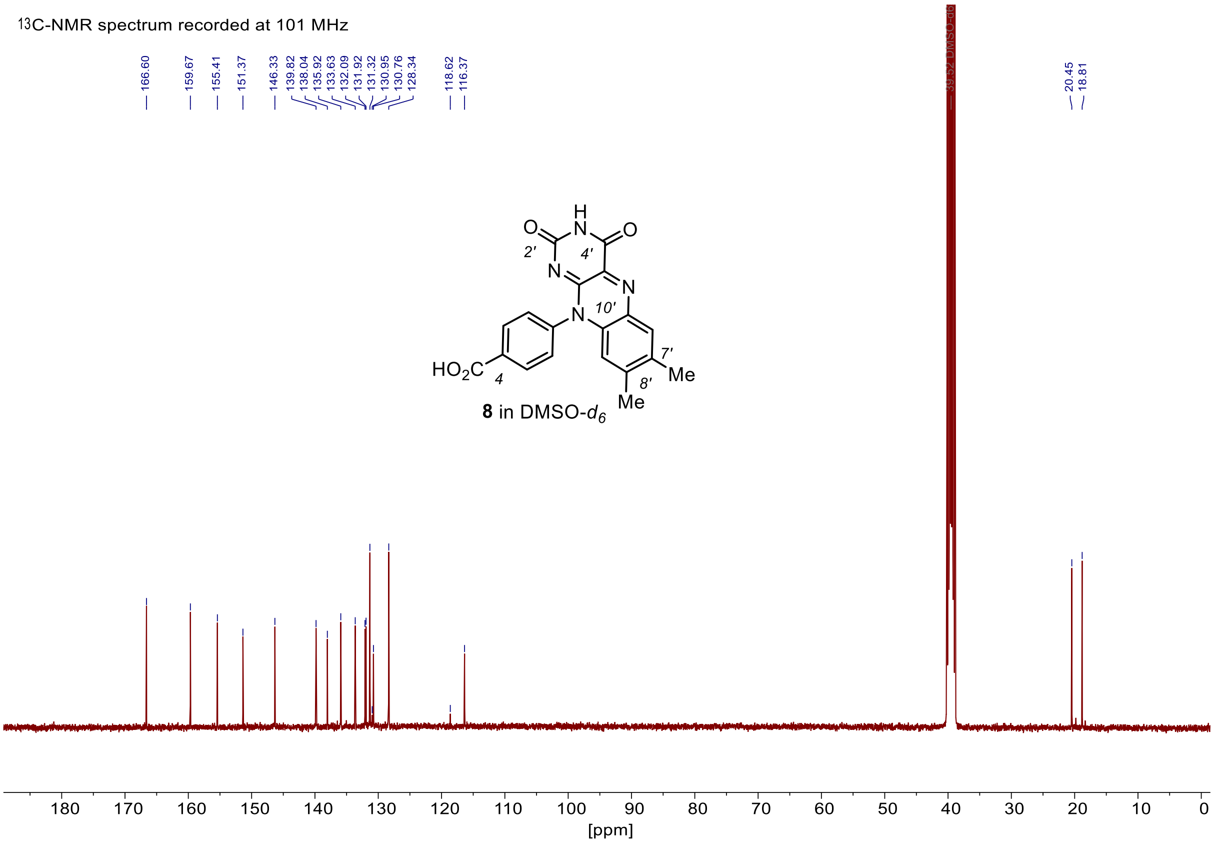

1H-NMR spectrum recorded at 500 MHz

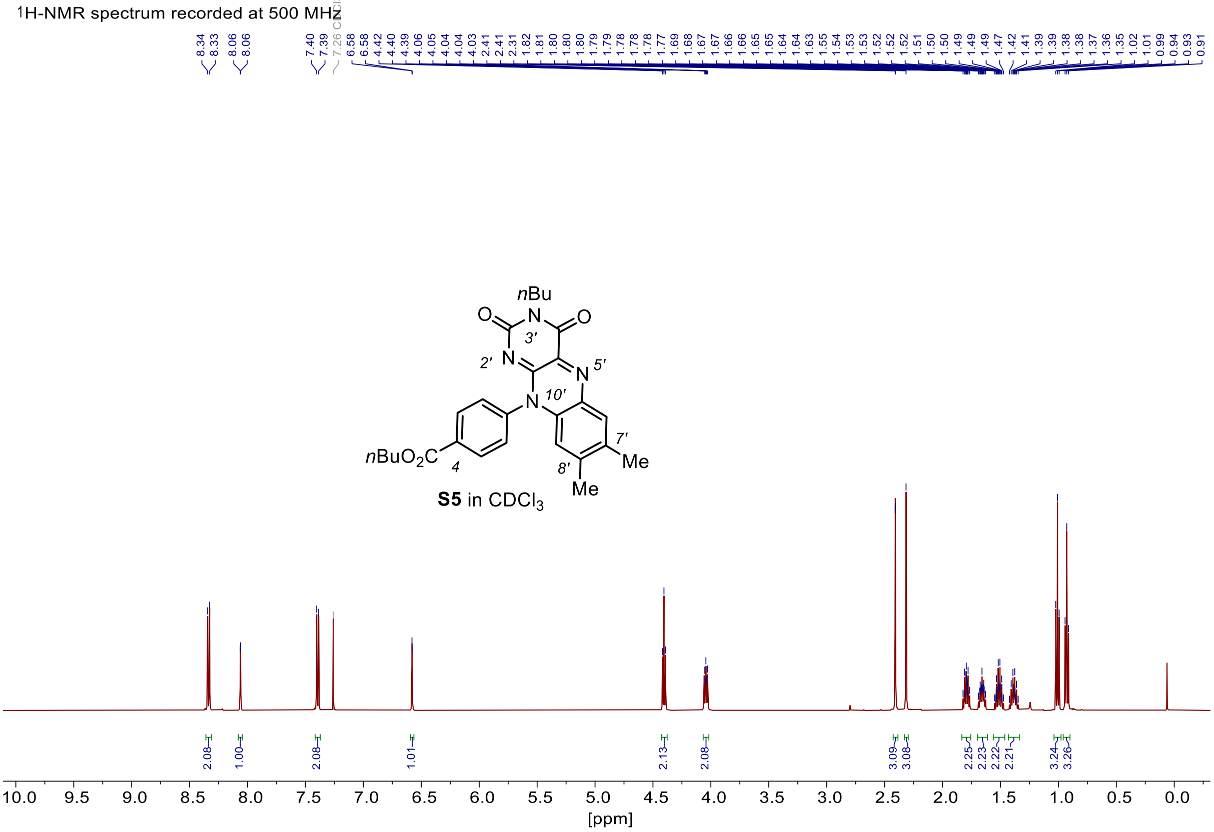

13C-NMR spectrum recorded at 126 MHz

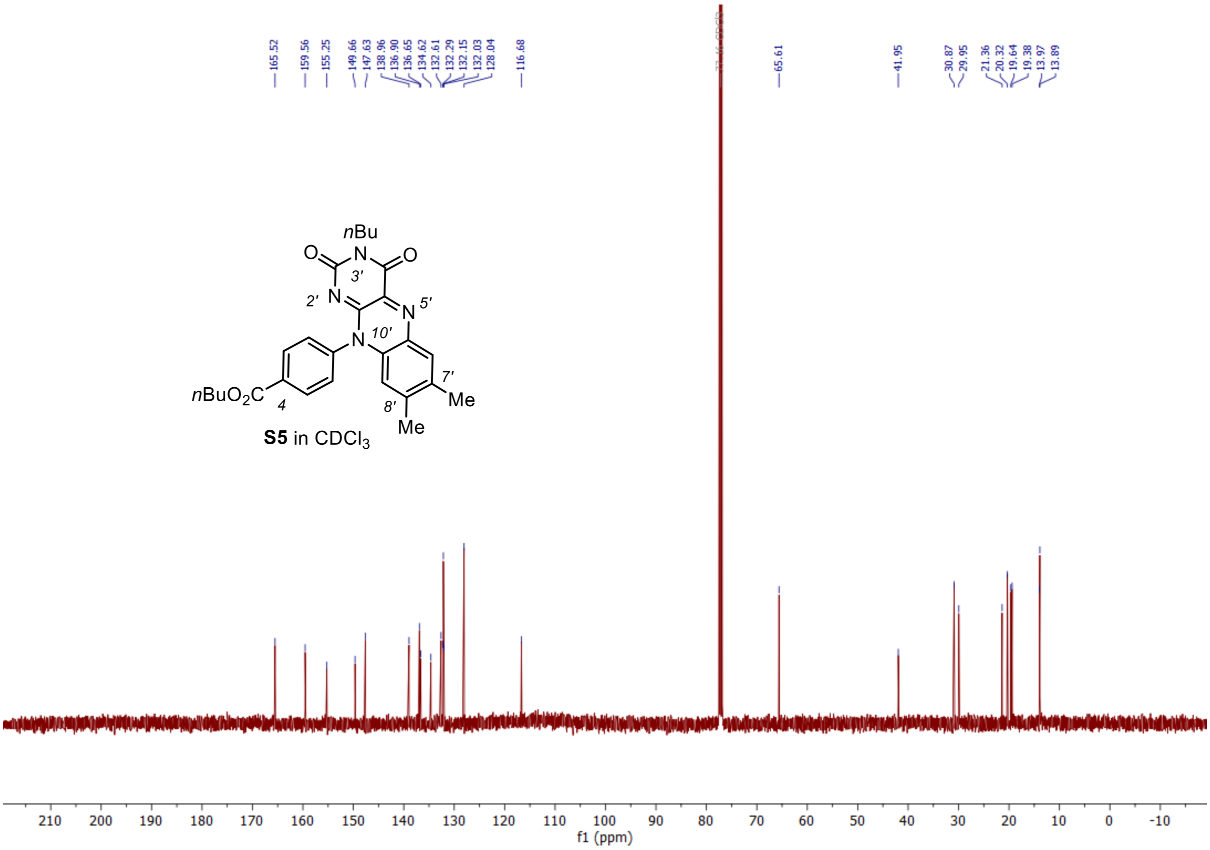

1H-NMR spectrum recorded at 300 MHz

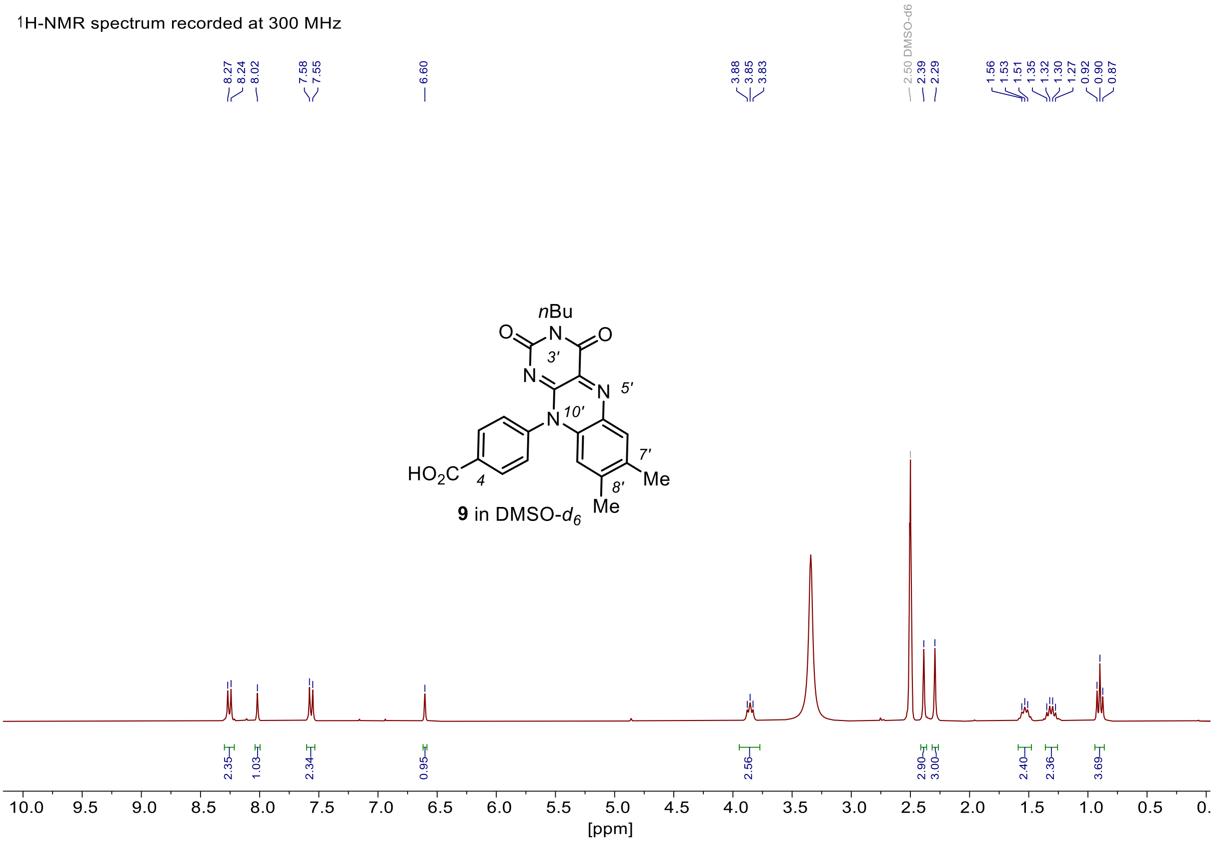

13C-NMR spectrum recorded at 126 MHz

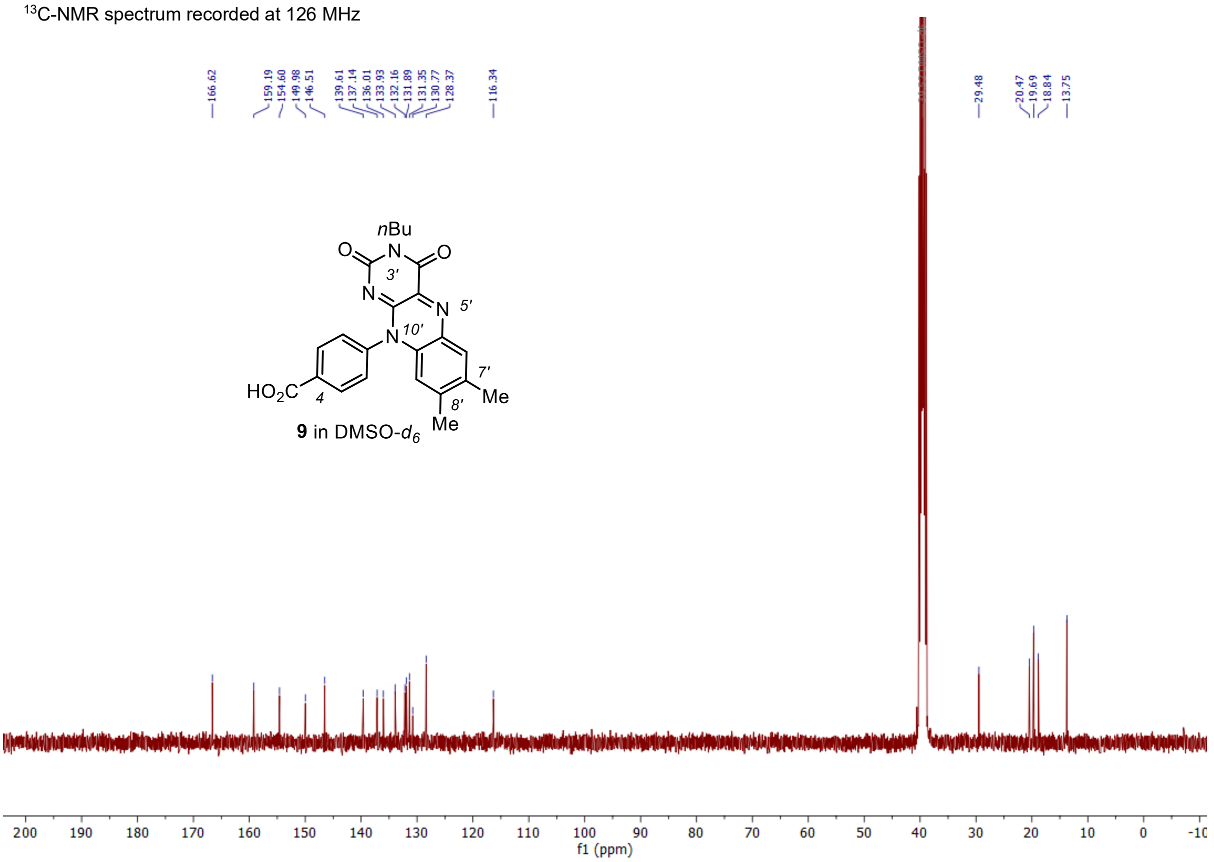

3. Synthesis of CIDNP-diads **2** and **3**

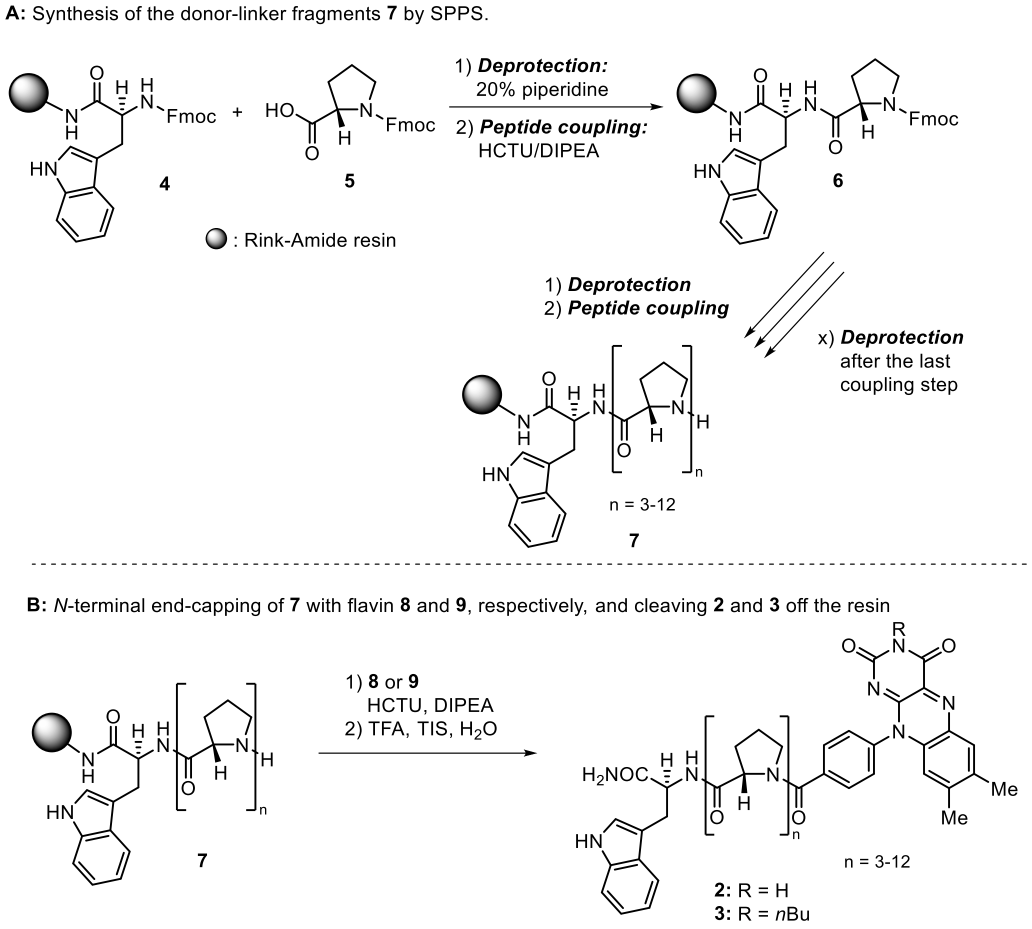

Scheme S2: A: Synthesis of the donor-linker peptides **7** employing fully automated SPPS conditions. **B:** *N*-terminal end-capping of peptide fragment **7** with flavins **8** and **9** respectively and cleaving the Pro-n *NH* diads **2** or Pro-n *Nn*Bu diads **3** off the resin.

3.1 General protocol for automatic SPPS of the Pro$_n$-TrpCONH$_2$ building blocks

The synthesis of all Pro-n-Trp building blocks **7** was performed on a fully automated Liberty Blue peptide synthesizer (CEM Corporation, Matthews, USA) with Rink-Amide resin (100-200 mesh, 0.54 mmol/g) from Novabiochem and referring to a 0.1 mmol reaction scale unless otherwise stated.

3.1.1 Resin swelling

The resin was pre-swollen for 30 min in 5 mL DMF.

3.1.2 Deprotection

5 mL of a 20% piperidine solution in DMF was added to the Fmoc-protected resin. The mixture was agitated for 5 min at 50 °C under microwave irradiation. The resin was washed with DMF (4x 5 mL).

3.1.3 Peptide coupling

5.00 eq. Fmoc-protected amino acid solution (0.2M in DMF), 5.00 eq. HCTU (0.5M in DMF) and 5.00 eq. DIPEA (1.0M in DMF) were added to the deprotected resin and under microwave irradiation at 50 °C agitated for 30 min. The coupling process was repeated once for every amino acid.

3.1.4 Control of the Amide Coupling by LC-MS

After every peptide coupling step, the success of the reaction was monitored by treating an aliquot of resin in ~1 mL cleavage solution (75% TFA, 21% DCM 3.3% H_2O, 1.7% TIPS) for 1h at rt followed by precipitating the peptide in ice-cold Et_2O. The peptide purity was determined by LC-MS on an Agilent Poroshell 120 EC-C18 (2.1 x 50 mm, 2.7 µm) column using a MeCN (A)/ water (B) solvent mixture complemented with 0.1% formic acid: 0 min 98% B, 14 min 50% B; flow rate flow rate: 0.5 mL/min.

3.2 *N*-Terminal Capping of 7 with Flavin-*F*10-Carboxylic Acids 8 and 9 and Cleavage of the Diads 2 and 3 from the Resin

In a 20 mL syringe reactor, the preloaded resin with the Pro-n-Trp peptides **7** were pre-swollen in 5 mL DMF for 30 min. 1.30 eq. Flavin-*F*10-carboxylic acid **8** or **9** were suspended in DMF to generate a theoretical 0.2 M solution. 1.0 M solution of 1.60 eq. DIPEA in DMF (1.80 mL) and 0.5 M solution of 1.40 eq. HCTU in DMF (900 µL) were premixed and added to the resin. The reacting mixture was thoroughly shaken on a vortex mixer for 6h. The reaction mixture was drained, and the resin was washed with 3x 20 mL DMF and 20 mL DCM. Cleavage of the peptide was done by adding 2 mL of cleavage solution (75% TFA, 21% CH_2Cl_2, 3.3% H_2O, 1.7% TIPS) to the resin and the mixture was agitated for 3h at rt. All volatiles were removed under reduced pressure. Ice-cold Et_2O was added until precipitation of an orange solid and was centrifuged at 4 °C (9500 rpm/4 min). The supernatant was decanted, and the peptide was washed twice with ice-cold Et_2O. The crude peptides **2** and **3** were dried under reduced pressure and purified by preparative HPLC employing a ReproSil Gold 120 C18 (250 x 10 mm) column using a MeCN (A)/ water (B) solvent mixture complemented with 0.1% TFA: 0

min 80% B, 30 min 40% B; flow rate 15 mL/min; **2a** t_R = 9.28 min; **2b** t_R = 9.35 min, **2c** t_R = 9.37 min; **2d** t_R = 9.24 min; **2e** t_R = 9.37 min; **3a** t_R = 11.91 min; **3c** t_R = 11.49 min; **3d** t_R = 5.57 min

3.3 Determination of Peptide Purity by LC-MS

The purity of each diad **2** and **3** was determined by LC-MS using an Agilent Poroshell 120 EC-C18 (2.1 x 50 mm, 2.7 µm) column and a MeCN (A)/ water (B) solvent mixture complemented with 0.1% formic acid at a flow rate of 0.5 mL/min at 40 °C. The specific method is stated for every diad **2** and **3**.

3.4 Analytical Data of Diads **2** and **3**

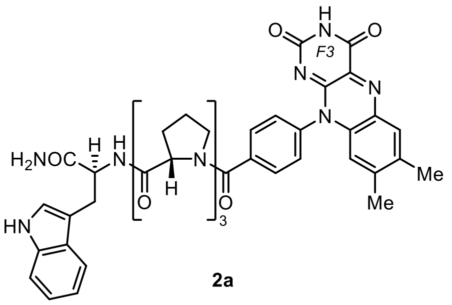

2a

F3-NH Pro-3 diad 2a: orange solid; **m.p.** = 230 °C (H_2O, decomp.); $[\alpha]^{21}$ = –41.0 (c = 0.1, DMSO); **^{1}H NMR** (600 MHz, MeOH-*d₄* δ 8.07 (s, 0.41 H), 8.03 (s, 0.88 H), 7.91 (d, *J* = 8.5 Hz, 0.95 H), 7.87 (d, J = 8.2 Hz, 0.58 H), 7.83 (m, 0.21 H), 7.78 (d, *J*= 8.1, 0.35 H), 7.73 (d, *J* = 7.6 Hz, 0.29 H), 7.64 (d, *J* = 8.0 Hz, 0.74 H), 7.60 (d, *J* = 7.7 Hz, 0.51 H), 7.53 (m, 2.2 H), 7.37 (d, *J* = 8.2 Hz, 0.53 H), 7.32 (t, *J* = 7.0 Hz, 0.74 H), 7.12 (m, 1.3 H), 7.07 (m, 1.4 H), 7.00 (m, 0.81 H), 6.70 (s, 0.73 H) 6.67 (s, 0.23 H), 4.65 (m, 0.90 H, overlapping with the MeOH signal), 4.56 (m, 1.5 H), 4.43 (d, *J* = 8.4 Hz, 0.45 H), 4.37 (d, *J* = 5.1 Hz, 0.46 H), 4.31 (m, 0.49 H), 4.15 (dd, *J* = 11.4, 4.1 Hz, 0.43 H), 4.06 (dd, *J* = 11.3, 6.24 Hz, 0.46 H), 3.99 (m, 0.38 H), 3.87 (m, 0.75 H), 3.76 (m, 3.2 H), 3.62 (m, 0.78 H), 3.55 (m, 1.0 H), 3.47 (m, 0.51 H), 2.46 (s, 2.8 H), 2.37 (s, 0.56 H), 2.35 (s, 2.5 H), 2.31 (s, 0.67 H), 2.03 (m, 11.2 H), 1.50 (m, 0.61 H) ppm; mixture of conformers; **^{13}C NMR** (151 MHz, MeOH-*d₄*) δ 173.6, 173.0, 172.3, 170.3, 162.0, 158.6, 153.0, 149.2, 139.4, 138.7, 136.0, 133.9, 132.2, 130.6, 130.5, 129.4, 129.2, 129.1, 124.8, 124.6, 122.7, 120.1, 119.3, 118.2, 112.5, 112.3, 62.29, 61.96, 60.61, 60.07, 59.66, 54.92, 51.75, 30.06, 29.69, 29.24, 27.90, 26.38, 26.01, 25.89, 22.36, 21.14, 19.40 ppm; **HRMS** (ESI^+) calcd. for $C_{45}H_{47}N_{10}O_7$ $[M+H]^+$ 839.3624, found 839.3611;

LC-ESI(+)-MS: column: Agilent Poroshell 120 EC-C18 (2.1 x 50 mm, 2.7 µm); mobile phase: MeCN (A)/ water (B) solvent mixture complemented with 0.1% formic acid; method: 98 → 40% B in 14 min; 0.5 mL/min, 40 °C; t_R = 9.28 min; m/z = $[M+H]^+$ = 839.4; Calcd. for $C_{45}H_{47}N_{10}O_7$ $[M+H]^+$ = 839.4.

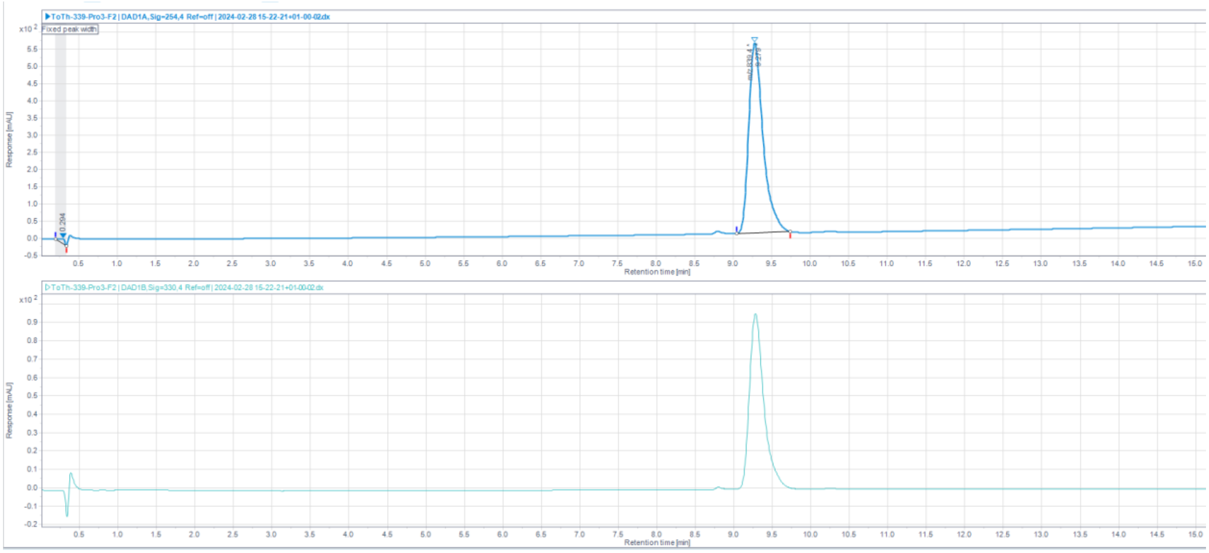

Figure S1: LC- UV Chromatograms of **2a** at 254 nm and 330 nm.

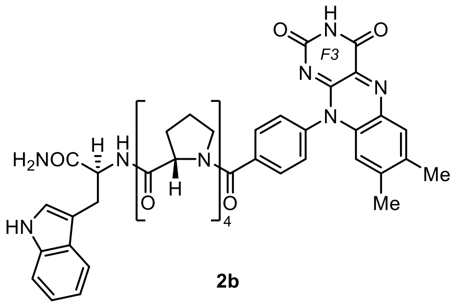

2b

F3-NH Pro-4 diad 2b: orange solid; **m.p.** = 233 °C (H_2O, decomp.); $[\alpha]^{21}$ = –86.6 (c = 0.5, DMSO); **^{1}H NMR** (500 MHz, MeOH-d_4) δ 7.95 (s, 1.0 H), 7.93 (m, 1.2 H), 7.90 (m, 0.75 H), 7.82 (d, *J* = 1.9 Hz, 0.04 H), 7.80 (d, *J* = 2.0 Hz, 0.05 H), 7.79 (d, *J* = 2.0 Hz, 0.14), 7.77 (d, *J* = 2.0 Hz, 0.14 H), 7.68 (d, *J* = 2.2 Hz, 0.07 H), 7.66 (m, 0.14 H), 7.65 (m, 0.10 H), 7.64 (d, *J* = 2.2 Hz, 0.14 H), 7.62 (m, 0.39 H), 7.58 (m, 3.41 H), 7.35 (m, 1.3 H), 7.08 (m, 4.0 H), 6.69 (s, 0.29 H), 6.69 (s, 0.72 H), 4.72 (dd, *J* = 8.5, 4.5 Hz, 0.62 H, overlapping MeOH-d_4 peak), 4.69 (dd, *J* = 6.6, 5.3 Hz, 0.34 H), 4.64 (t, *J* = 6.0 Hz, 0.66 H), 4.59 (m, 0.46 H), 4.56 (d, *J* = 4.4 Hz, 0.17 H), 4.48 (m, 1.5 H), 4.37 (m, 0.34 H), 4.34 (dd, *J* = 8.4, 4.2 Hz, 0.57 H), 4.29 (m, 0.27 H), 3.77 (m, 9.4 H), 2.43 (s, 3.5 H), 3.41 (s, 1.1 H), 2.35 (s, 3.3 H), 2.33 (s, 0.69 H), 2.03 (m, 20.4 H) ppm, mixture of conformers; **^{13}C NMR** (126 MHz, MeOH-d_4) δ 177.1, 176.5, 176.5, 174.9, 174.5, 174.0, 173.8, 173.7, 173.5, 173.4, 173.2, 173.1, 172.9, 172.8, 172.5, 172.3, 172.1, 171.4, 170.2, 161.7, 158.4, 157.9, 152.9, 152.8, 149.3, 149.1, 140.2, 139.4, 138.8, 138.7, 138.6, 138.5, 138.1, 138.0, 137.9, 135.9, 135.7,133.9, 133.8, 132.2, 130.7, 130.6, 130.5, 130.2, 129.9, 129.5, 129.3, 129.2, 129.1, 129.0, 128.9, 124.8, 124.6, 124.4, 124.3, 122.7, 122.6, 122.4, 120.2, 120.1, 119.9, 119.8, 119.3, 119.2, 118.3, 118.2, 115.9, 115.8, 112.5, 112.4, 112.3, 111.8, 111.4, 110.6, 110.3, 110.2, 64.07, 62.41, 62.30, 61.98, 61.77, 61.38, 60.47, 60.30, 60.16, 60.09, 59.90, 59.79, 59.75, 59.71, 59.56, 57.03, 54.89, 54.85, 54.57, 51.85, 51.74, 33.06, 32.07, 31.41, 30.76, 30.74, 30.70, 30.59, 30.46, 30.40, 30.20, 30.00, 29.88, 29.83, 29.72, 29.17, 29.12, 19.01, 28.93, 27.85, 27.73, 26.48,

26.35, 26.13, 26.02, 25.99, 25.94, 25.91, 25.82, 25.78, 25.64, 23.86, 23.73, 21.18, 21.16, 19.43, 19.41, 18.36, 18.19 ppm; **HRMS** (ESI$^+$) calcd. for $C_{50}H_{54}N_{11}O_8$ $[M+H]^+$ 936.4151, found 936.4133;

LC-ESI(+)-MS: column: Agilent Poroshell 120 EC-C18 (2.1 x 50 mm, 2.7 µm); mobile phase: MeCN (A)/ water (B) solvent mixture complemented with 0.1% formic acid; method: 98 → 40% B in 14 min; 0.5 mL/min, 40 °C; t_R = 9.35 min; m/z = $[M+H]^+$ = 936.4; Calcd. for $C_{50}H_{54}N_{11}O_8$ $[M+H]^+$ = 936.4.

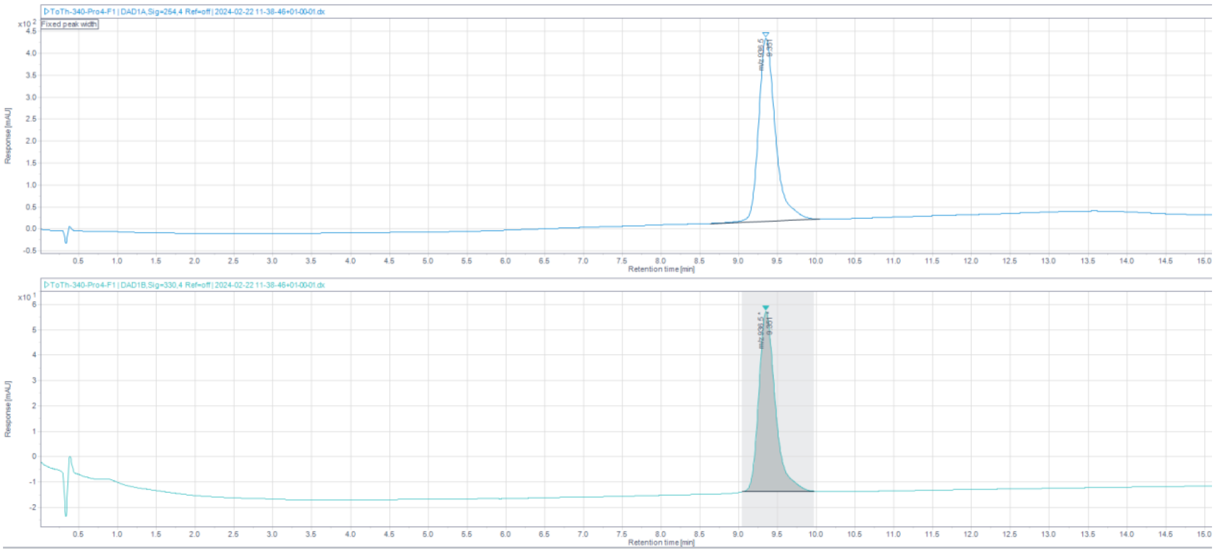

Figure S2: LC-UV chromatogram of **2b** at 254 nm and 330 nm.

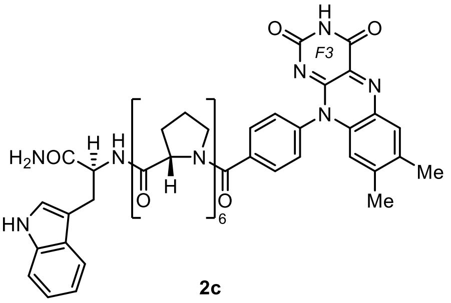

2c

F3-NH Pro-6 diad 2c: orange solid; **m.p.** = 235 °C (H_2O, decomp.); $[\alpha]^{21}$ = –109.0 (c = 1, DMSO); **^{1}H NMR** (600 MHz, MeOH-*d$_4$*) δ 8.03 (s, 0.70 H), 8.01 (s, 0.30 H), 7.93 (d, *J* = 8.1 Hz, 0.75 H), 7.89 (d, *J* = 8.1 Hz, 0.72 H), 7.78 (d, *J* = 7.4 Hz, 0.28 H), 7.62 (m, 1.3 H), 7.54 (d, *J* = 8.1 Hz, 1.7 H), 7.50 (m, 0.33 H), 7.36 (t, *J* = 8.5 Hz, 1.0 H), 7.12 (q, *J* = 6.5, 5.7 Hz, 2.0 H), 7.03 (dt, *J* = 15.9, 7.5 Hz, 1.0 H), 6.71 (s, 1.0 H), 4.76 (m, 0.76 H, overlapping MeOH-*d$_4$* peak), 4.71 (m, 0.66 H), 4.64 (m, overlapping signals, 1.5 H), 4.55 (m, 0.49 H), 4.49 (m, 0.61 H), 4.43 (m, 0.20 H), 4.36 (d, *J* = 8.0 Hz, 0.23 H), 4.33 (m, 0.63 H), 4.15 (d, *J* = 7.2 Hz, 0.05 H), 4.06 (m, 0.07 H), 3.96 (m, 0.74 H), 3.77 (m, overlapping signals, 10.2 H), 2.81 (m, 0.27 H), 2.45 (s, 3.43 H), 2.35 (s, 3.58 H), 2.00 (m, 21.4 H), 1.20 (m, 0.84 H) ppm; mixture of conformers; **^{13}C NMR** (151 MHz, MeOH-*d$_4$*) δ 176.5, 173.8, 173.5, 173.1, 172.3, 172.2, 171.4, 170.3, 162.0, 158.6, 153.0, 149.2, 139.4, 138.8, 138.7, 138.6, 138.5, 138.1, 136.0, 133.9, 132.2, 130.6, 130.5, 129.4, 129.2, 129.0, 124.8, 124.4, 122.7, 122.6, 120.1, 119.2, 118.2, 112.5, 112.4, 110.2, 62.27, 62.08, 61.71, 60.45, 60.11, 59.77, 59.64, 59.59, 59.52, 54.89, 51.76, 36.52, 33.06, 32.11, 30.76, 30.30, 29.99, 29.74, 29.17, 29.08, 28.98, 28.12, 27.83, 26.37, 26.09, 25.97, 25.93,

25.84, 25.78, 23.80, 21.15, 19.40, 14.44 ppm; **HRMS** (ESI$^+$) calcd. for $C_{60}H_{68}N_{13}O_{10}$ $[M+H]^+$ 1130.5207, found 1130.5192;

LC-ESI(+)-MS: column: Agilent Poroshell 120 EC-C18 (2.1 x 50 mm, 2.7 µm); mobile phase: MeCN (A)/ water (B) solvent mixture complemented with 0.1% formic acid; method: 98 → 40% B in 14 min; 0.5 mL/min, 40 °C; t_R = 9.37 min; m/z = $[M+H]^+$ = 1030.5; Calcd. for $C_{60}H_{68}N_{13}O_{10}$ $[M+H]^+$ = 1030.5.

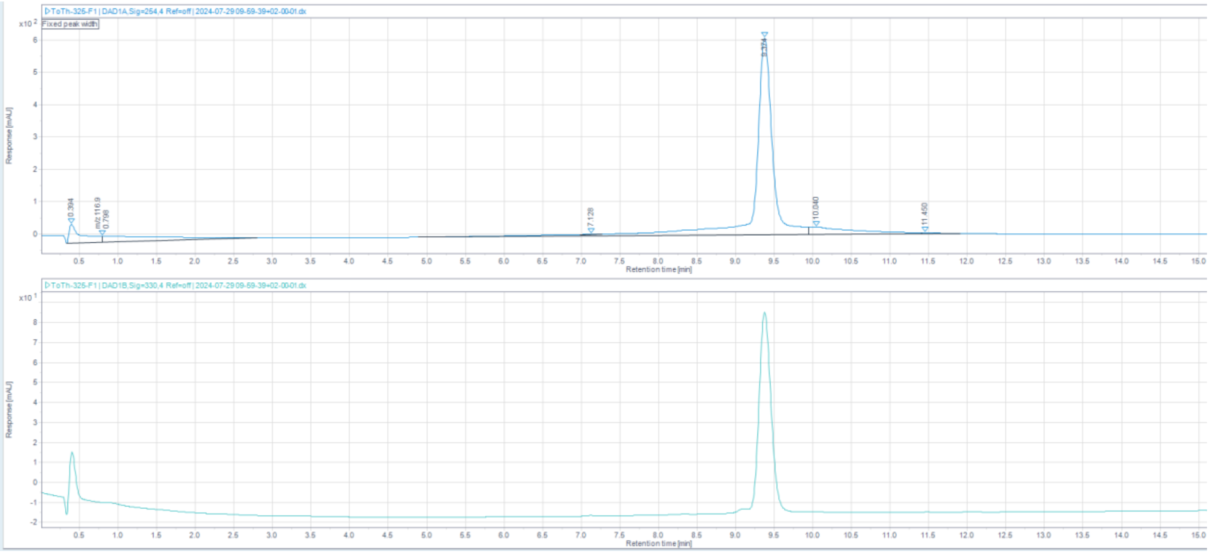

Figure S3: LC-UV chromatogram of **2c** at 254 nm and 330 nm.

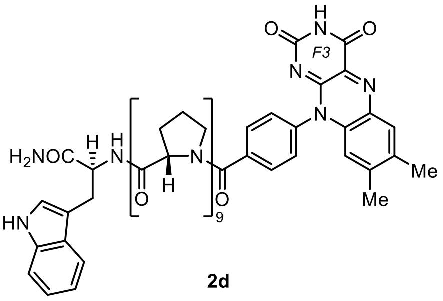

2d

F3-NH Pro-9 diad 2d: orange solid; **m.p.** = 234 °C (H_2O, decomp.); $[\alpha]^{21}$ = –123.2 (c = 0.5, DMSO); **^{1}H NMR** (600 MHz, MeOH-d_4) δ 7.99 (s, 0.76 H), 7.97 (s, 0.31 H), 7.94 (m, 0.72 H), 7.91 (m, 0.79 H), 7.79 (d, J = 8.3 Hz, 0.43 H), 7.64 (d, J = 8.4 Hz, 0.68 H), 7.61 (d, J = 8.0 Hz, 0.95 H), 7.57 (m, 2.0 H), 7.53 (d, J = 8.4 Hz, 0.45 H), 7.35 (t, J = 9.1 Hz, 1.4 H), 7.11 (m, 2.8 H), 7.02 (m, 1.6 H), 6.70 (s, 0.72 H), 6.69 (s, 0.23 H), 4.80 (dd, J = 8.6, 4.1 Hz, 1.1 H), 4.77 (m, 0.53 H), 4.68 (m, 5.2 H), 4.62 (m, overlapping signals, 2.0 H), 4.57 (d, J = 9.7 Hz, 0.78 H), 4.47 (dd, J = 8.2, 4.5 Hz, 1.2 H), 4.38 (m, 0.54 H), 4.34 (d, J = 8.1 Hz, 0.48 H), 4.31 (dd, J = 8.3, 4.1 Hz, 0.91 H), 4.29 (dd, J = 5.7, 2.5 Hz, 0.52 H), 3.95 (m, 1.0 H), 3.71 (m, 26.0 H), 3.25 (dd, J = 14.8, 6.3 Hz, 1.5 H), 2.44 (s, 2.4 H), 2.43 (s, 0.78 H), 2.35 (s, 2.3 H), 2.34 (s, 1.0 H), 2.25 (m, 11 H), 1.99 (m, 42 H), 1.49 (m, 0.91 H), 1.43 (m, 0.46 H), 1.36 (m, 1.4 H), 1.17 (m, 0.79 H) ppm; mixture of conformers; **^{13}C NMR** (151 MHz, MeOH-d_4) δ 173.8, 173.5, 172.1, 170.2, 152.9, 138.8, 138.0, 133.9, 132.2, 130.6, 129.4, 124.8, 122.7, 120.1, 119.2, 118.2, 112.6, 110.2, 62.30, 60.12, 59.61,54.87,59.61, 54.87, 51.74, 35.31, 29.98, 29.72, 29.03, 27.80, 26.36, 25.80, 22.77, 21.17, 19.42 ppm; **HRMS** (ESI$^+$) calcd. for $C_{75}H_{89}N_{16}O_{13}$ $[M+H]^+$ 1422.6790, found 1422.6764;

LC-ESI(+)-MS: column: Agilent Poroshell 120 EC-C18 (2.1 x 50 mm, 2.7 µm); mobile phase: MeCN (A)/ water (B) solvent mixture complemented with 0.1% formic acid; method: 98 → 40% B in 14 min; 0.5 mL/min, 40 °C; t_R = 9.24 min; m/z = $[M+2H]^{2+}$ = 711.5; Calcd. for $C_{75}H_{90}N_{16}O_{13}$ $[M+2H]^{2+}$ = 711.3.

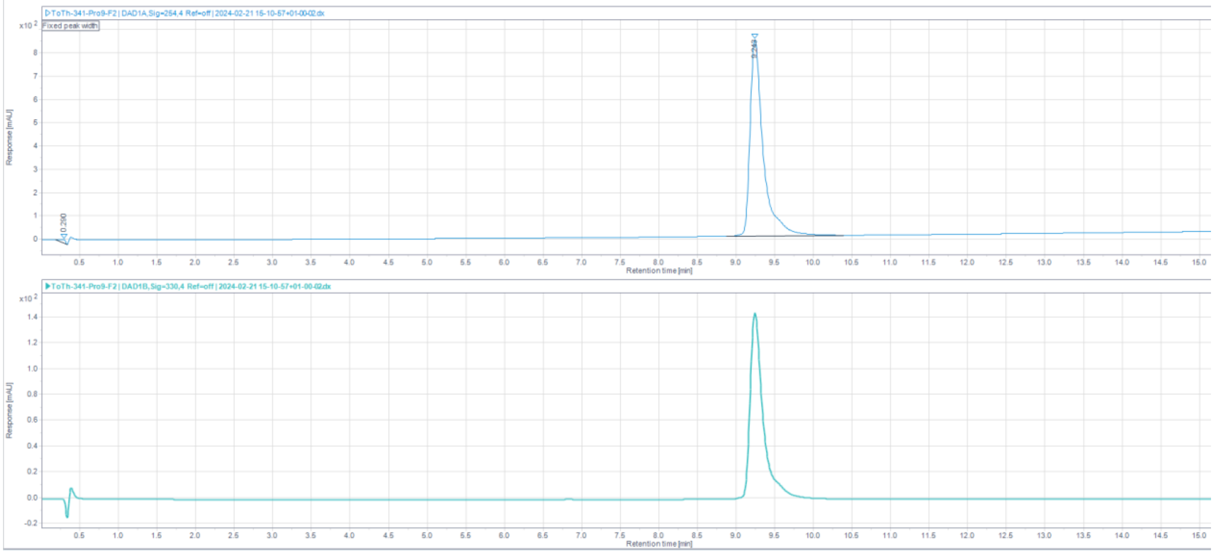

Figure S4: LC-UV chromatogram of **2d** at 254 nm and 330 nm.

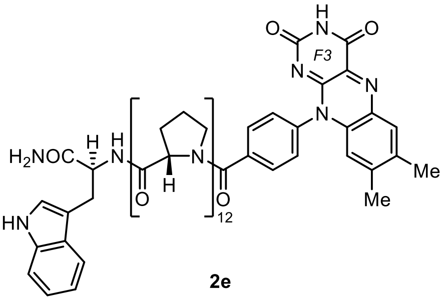

2e

F3-NH Pro-12 diad 2e: orange solid; **m.p.** = 236 °C (H_2O, decomp.); $[\alpha]^{21}$ = –123.2 (c = 0.5, DMSO); **^{1}H NMR** (600 MHz, MeOH-*d₄*) δ 8.02 (s, 0.77 H), 8.00 (s, 0.23 H), 7.78 (d, *J* = 8.1 Hz, 0.29 H), 7.63 (m, 1.3 H), 7.55 (d, *J* = 8.1 Hz, 1.8 H), 7.50 (m, 0.18 H), 7.35 (m, 1.1 H), 7.12 (m, 2.3 H), 7.03 (m, 0.94 H), 6.95 (m, 0.14 H), 6.90 (dd, *J* = 15.4, 8.1 Hz, 0.23 H), 6.71 (s, 1.0 H), 4.80 (dd, *J* = 8.6, 4.4 Hz, 0.96 H), 4.71 (m, 8.6 H), 4.62 (t, *J* not detectable due to overlapping signals, 1.1 H), 4.56 (m, 0.87 H), 4.48 (m, 1.1 H), 4.41 (m, 1.3 H), 4.33 (m, 0.94 H), 3.96 (t, *J* = 8.4 Hz, 0.61 H), 3.77 (m, 14 H), 3.62 (m, 14 H), 2.45 (s, 3 overlapping signals, 4.4 H), 2.35 (s, overlapping signals, 3.3 H), 2.24 (m, 11 H), 2.03 (m, 53 H) ppm; mixture of conformers; **^{13}C NMR** (126 MHz, MeOH-*d₄*) δ 176.5, 173.7, 173.5, 172.8, 172.5, 172.3, 172.2, 172.1, 171.4, 170.3, 161.8, 158.6, 153.0, 149.2, 149.0, 140.2, 139.4, 138.8, 138.7, 138.5, 138.0, 135.9, 135.7, 133.9, 133.8, 132.2, 130.9, 130.8, 130.6, 130.5, 129.9, 129.5, 129.4, 129.3, 129.0, 124.8, 124.4, 122.7, 120.1, 120.0, 119.3, 118.3, 112.5, 112.4, 111.4, 110.3, 71.39. 66.47, 64.06, 62.23, 62.08, 61.70, 60.43, 60.10, 59.76, 59.65, 59.61, 59.50, 56.99, 54.87, 51.75, 36.52, 34.93, 33.04, 32.08, 31.34, 30.81, 30.75, 30.58, 30.42, 30.30, 30.21, 29.73, 29.16, 29.09, 29.03, 28.33, 28.10, 26.90, 26.37, 26.07, 25.95, 25.92, 25.79, 25.73, 23.79, 23.72, 22.85, 22.34, 21.14, 21.01, 19.42, 19.40 ppm; **HRMS** (ESI$^+$) calcd. for $C_{90}H_{110}N_{19}O_{16}$ $[M+H]^+$ 1712.8378, found 1712.8372.

LC-ESI(+)-MS: column: Agilent Poroshell 120 EC-C18 (2.1 x 50 mm, 2.7 µm); mobile phase: MeCN (A)/ water (B) solvent mixture complemented with 0.1% formic acid; method: 98 → 40% B in 14 min; 0.5 mL/min, 40 °C; t_R = 9.37 min; m/z = $[M+2H]^{2+}$ = 857.4; Calcd. for $C_{90}H_{111}N_{19}O_{16}$ $[M+2H]^{2+}$ = 856.9.

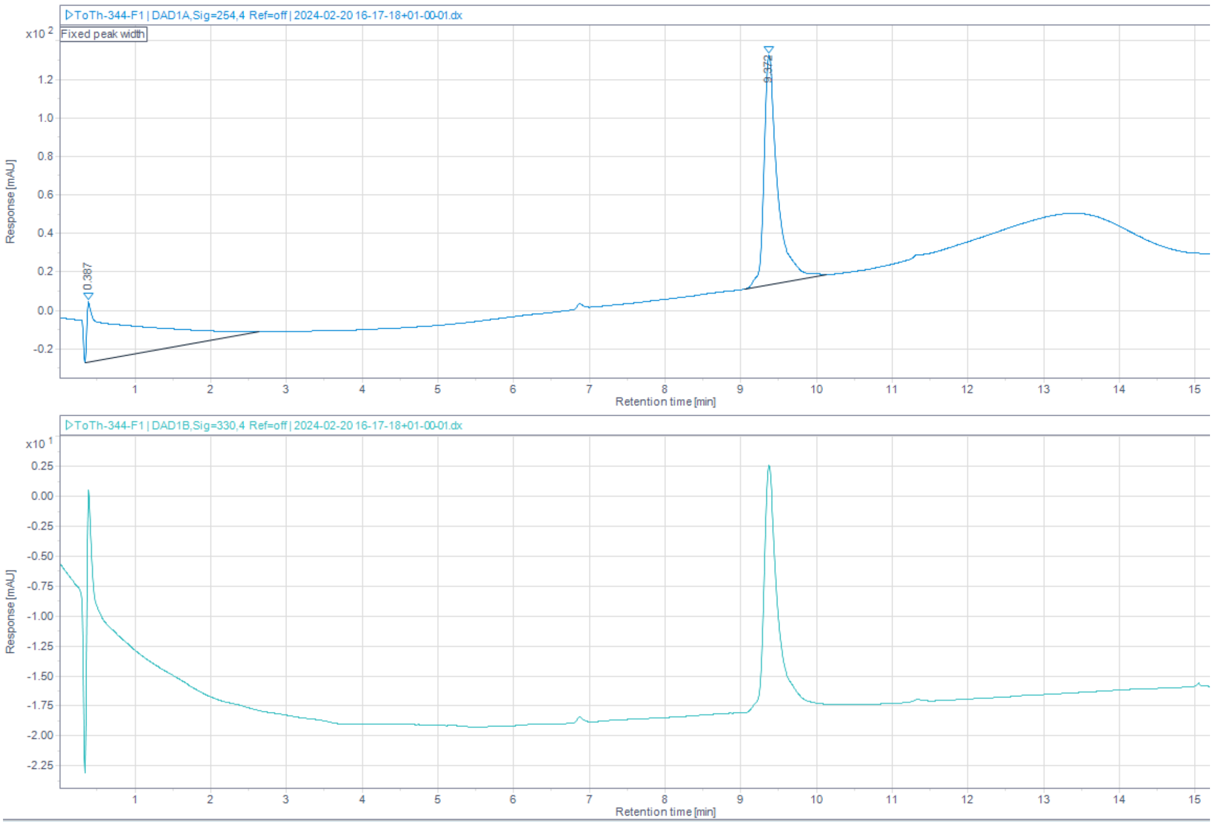

Figure S5: LC-UV chromatogram of **2e** at 254 nm and 330 nm.

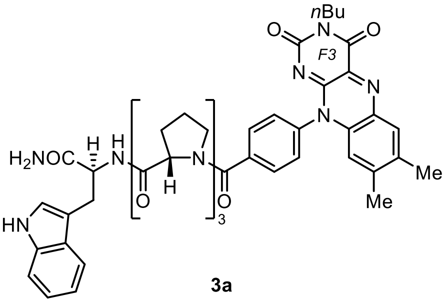

3a

F3-N*n*Bu Pro-3 diad 3a: orange solid; **m.p.** = 230 °C (H_2O, decomp.); $[\alpha]^{21}$ = –67.0 (c = 1, DMSO); **^{1}H NMR** (600 MHz, MeOH-*d*$_4$) δ 8.03 (s, 1H), 7.91 (m, 0.90 H), 7.87(two d overlaying, 0.51 H), 7.82 (d, *J* = 8.0 Hz, 0.08 H), 7.78 (m, 0.19 H), 7.73 (t, *J* = 8.1 Hz, 0.17 H), 7.61 (at least three conformers overlapping, 1.05 H), 7.52 (m, 2 H), 7.37 (d, *J* = 8.1 Hz, 0.47 H), 7.32 (m, 0.54 H), 7.12 (m, 1.5 H), 7.06 (m, 1.0 H), 6.99 (m, 0.58 H), 6.79 (s, 0.07 H), 6.71 (s, 0.42 H), 6.70 (s, 0.24 H), 6.69 (s, 0.19 H), 4.75 (m, 0.23 H, overlapping signals with MeOH-*d*$_4$), 4.65 (t, *J* = 6.0 Hz, 0.50 H), 4.57 (m, 1.3 H), 4.43 (d, *J* = 8.0 Hz, 0.24 H), 4.37 (dd, *J* = 8.4, 4.2 Hz, 0.60 H), 4.30 (dd, *J* = 8.6, 4.4 Hz, 0.17 H), 4.06 (t, *J* = 7.4 Hz, 0.45 H), 4.01 (q, *J* = 6.0, 4.0 Hz, 1.8 H), 3.87 (q, *J* = 7.7, 6.8 Hz, 0.69 H), 3.76 (m, 2.9 H), 3.63 (dq, *J* = 15.4, 8.5, 7.3 Hz, 0.83), 3.42 (m, 1.1 H), 3.21(m, 0.85 H, overlapping signals), 2.94 (t, *J* = 10.2 Hz, 0.08 H), 2.88 (t, *J* = 10.3 Hz, 0.20 H), 2.52 (m, 0.42 H), 2.46 (s, 0.36 H), 2.45

(s, 1.75 H), 2.44 (s, 0.78 H), 2.40 (m, 0.67 H), 2.37 (s, 0.26 H), 2.35 (s, 2.1 H), 2.31 (s, 0.63 H), 2.00 (m, 10.9 H), 1.66 (m, 2.0 H), 1.41 (q, *J* = 7.4 Hz, 2.1 H), 0.97 (tt, *J* = 7.3, 3.4 Hz, 3.0 H) ppm; mixture of conformers; **^{13}C NMR** (151 MHz, MeOH-*d$_4$*) δ 176.5, 173.8, 173.7, 172.3, 170,3, 161.7, 157.8, 151.6, 149.1, 139.4, 138.6, 138.0, 136.2, 133.9, 132.2, 130.6, 130.5, 129.5, 129.3, 129.1, 124.8, 122.7, 122.6, 120.1, 119.3, 118.1, 112.5, 110.3, 62.29, 62.01, 60.61, 60.24, 60.07, 59.66, 57.10, 54.91, 51.74, 42.70, 32.13, 30.93, 30.05, 29.69, 29.36, 29.24, 29.10, 27.90, 26.37, 26.23, 26.00, 25.89, 23.87, 21.21, 21.13, 19.39, 14.16 ppm; **HRMS** (ESI$^+$): calcd. for $C_{49}H_{55}N_{10}O_7^+$ $[M+H]^+$ 895.4250, found 895.4265.

LC-ESI(+)-MS: column: Agilent Poroshell 120 EC-C18 (2.1 x 50 mm, 2.7 µm); mobile phase: MeCN (A)/ water (B) solvent mixture complemented with 0.1% formic acid; method: 98 → 40% B in 14 min; 0.5 mL/min, 40 °C; t_R = 11.91 min; m/z = $[M+H]^+$ = 895.4; Calcd. for $C_{49}H_{55}N_{10}O_7$ $[M+H]^+$ = 895.4.

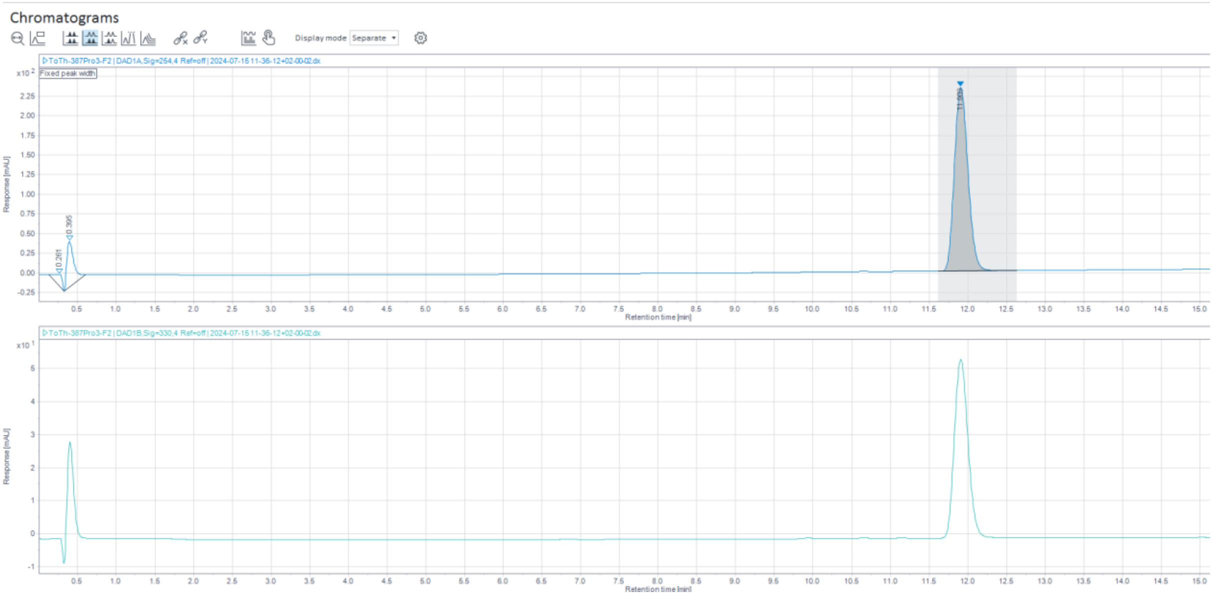

Figure S6: LC- UV chromatogram of **3a** at 254 nm and 330 nm.

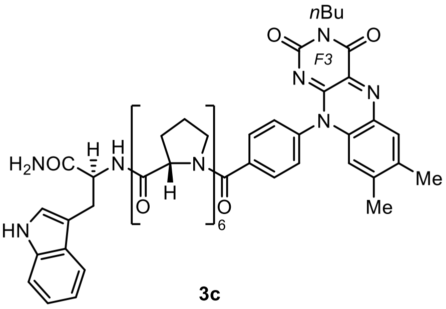

3c

F3-N*n*Bu Pro-6 diad 3c: orange solid; **m.p.** = 227 °C (H_2O, decomp.); $[\alpha]^{21}$ = –105 (c = 1, DMSO); **^{1}H NMR** (600 MHz, MeOH-*d$_4$*) δ 8.03 (s, 0.66 H), 8.01 (s, 0.28 H), 7.93 (dd, *J* = 8.5, 2.1 Hz, 0.7H), 7.90 (m, 0.75 H), 7.79 (m, 0.25 H), 7.65 (d, *J* = 7.9 Hz, 0.20 H), 7.61 (m, 0.99 H), 7.54 (dd, *J* = 8.1, 2.5 Hz, 1.68 H), 7.49 (dd, *J* = 8.0, 2.2 Hz, 0.31 H), 7.35 (m, 0.98 H), 7.11 (m, 2.0 H), 7.03 (m, 1.1 H), 6.72 (s, 1.0 H), 4.81 (ddd, *J* = 17.5, 8.8, 4.0 Hz, 1.2 H, overlapping signals with MeOH-*d$_4$*), 4.75 (m, 0.46 H), 4.71 (dd, *J* = 8.7, 4.4 Hz, 0.63 H), 4.63 (t, *J* = 5.7, 0.93 H, overlapping signals), 4.55 (dq, J = 8.4, 4.0 Hz, 0.51 H), 4.48 (m, 0.67 H), 4.43 (t, *J* = 6.7 Hz, 0.23 H), 4.36 (d, *J* = 8.1,

0.22 H), 4.32 (dt, J = 6.6, 3.4 Hz, 0.72 H), 4.01 (t, J = 7.5 Hz, 2.0 H), 3.95 (m, 0.72 H), 3.85 (m, 1.3 H), 3.79 (m, 1.3 H), 3.74 (m, 3.5 H), 3.66 (m, 2.2 H), 3.59 (m, 1.1 H), 3.50 (m, 0.94 H), 3.25 (m, 1.48 H), 2.82 (t, J = 10.2 Hz, 0.24 H), 2.45 (s, 3.5 H. overlapping signals), 2.35 (s, 3.3 H), 2.03 (m, overlapping signals, 22.1 H), 1.65 (q, J = 7.6 Hz, 2.1 H), 1.48 (m, 0.25 H), 1.41 (q, J = 7.5 Hz, 2.0 H), 1.20 (m, 0.61 H), 0.97 (t, J = 7.4 Hz, 3.0 H) ppm; mixture of conformers; **^{13}C NMR** (151 MHz, MeOH-d_4) δ 177.1, 176.5, 173.8, 173.5, 172.4, 172.3, 172.3, 172.2, 170.3, 161.7, 157.8, 151.6, 149.1, 148.9, 139.5, 138.6, 138.5, 138.3, 138.3, 138.1, 138.0, 136.2, 136.0, 133.9, 132.2, 130.6, 130.5, 129.9, 129.5, 129.3, 129.0, 128.7, 124.8, 124.3, 122.7, 122.6, 120.1, 119.2, 118.1, 118.0, 112.5, 112.4, 111.4, 110.2, 62.28, 62.07, 61.72, 60.45, 60.11, 59.77, 59.64, 59.59, 59.52, 54.89, 42.71, 42.57, 30.99, 30.93, 30.76, 29.99, 29.74, 29.25, 29.17, 29.09, 29.02, 28.98, 28.35, 27.84, 26.38, 26.09, 25.98, 25.93, 25.84, 25.80, 25.75, 22.35, 21.21, 21.13, 19.39, 14.20, 14.16 ppm; **HRMS** (ESI$^+$): calcd. for $C_{64}H_{76}N_{13}O_{10}^+$ [M+H]$^+$ 1186.5833, found 1186.5826;

LC-ESI(+)-MS: column: Agilent Poroshell 120 EC-C18 (2.1 x 50 mm, 2.7 µm); mobile phase: MeCN (A)/ water (B) solvent mixture complemented with 0.1% formic acid; method: 98 → 40% B in 14 min; 0.5 mL/min, 40 °C; t_R = 11.49 min; m/z = [M+H]$^+$ =1186.6; Calcd. for $C_{64}H_{76}N_{13}O_{10}$ [M+H]$^+$ = 1186.6.

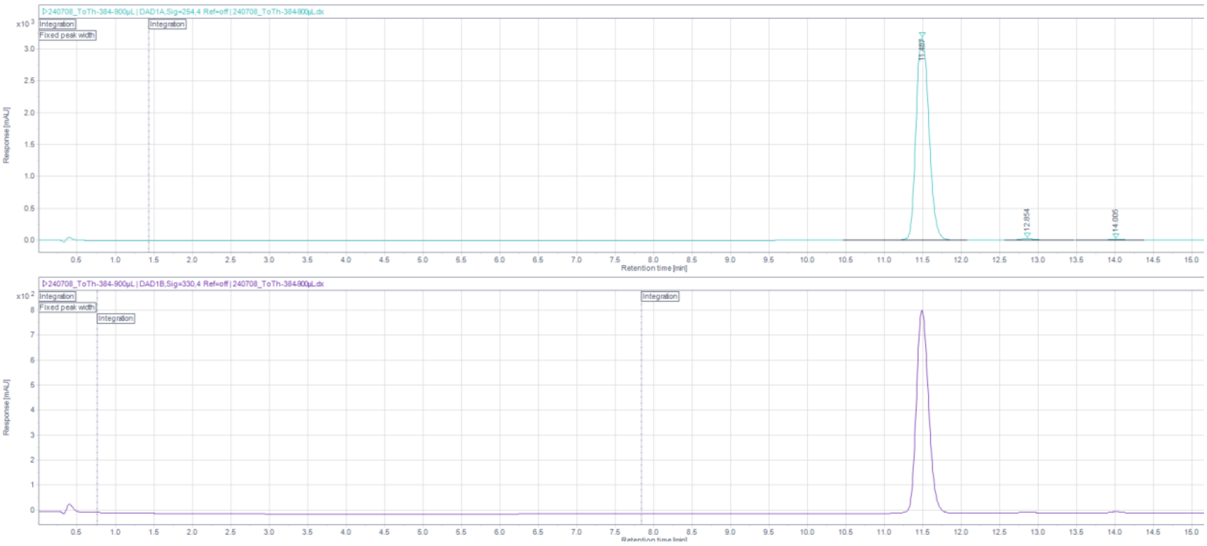

Figure S7: LC-UV chromatogram of **3c** at 254 nm and 330 nm.

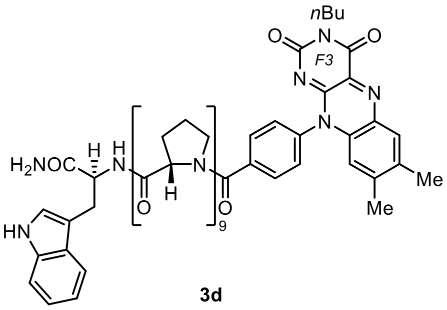

3d

F3-N*n*Bu Pro-9 diad 3d: orange solid; **m.p.** = 220 °C (H_2O, decomp.); $[\alpha]^{21}$ = –132.0 (c = 0.1, DMSO); **^{1}H NMR** (600 MHz, MeOH-d_4) δ 8.04 (s, 0.70 H), 8.02 (s, 0.24 H), 7.93 (m, 0.70 H), 7.90 (d, J = 8.1 Hz, 0.67 H), 7.79 (d, J = 7.6 Hz, 0.24 H), 7.62 (d, J = 7.8 Hz, 0.72 H), 7.54 (d, J = 8.3 Hz, 1.5 H), 7.49 (d, J = 8.0 Hz, 0.21 H), 7.36 (d, J = 8.5 Hz, 0.52 H), 7.12 (m, 1.6 H), 7.04 (t, J = 7.4 Hz, 0.81 H), 6.72 (s, 0.91 H), 4.80 (m, 2.0 H), 4.67 (m, 12.3 H), 4.55 (m, 1.0 H), 4.49 (m, 0.53 H), 4.32 (m, 0.66 H), 4.03 (t, J = 7.6 Hz, 1.8 H), 3.73 (m, 16.1 H), 2.46 (s, 2.0 H), 2.46 (s, 1.0 H), 2.36 (s, 3.0 H),2.08 (m, 34 H), 1.66 (q, J = 7.6 Hz, 2.1 H), 1.42 (q, J = 7.3 Hz, 2.0 H), 0.89 (t, J = 7.5 Hz, 3.0 H) ppm; mixture of conformers; **^{13}C NMR** (126 MHz, MeOH-d_4) δ 172.1, 170.3, 151.6, 136.2, 134.0, 132.3, 130.6, 130.5, 129.5, 129.3, 124.8, 119.2, 118.1, 69.34, 60.12, 59.78, 59.63, 56.49, 51.76, 49.57, 49.43, 49.36, 49.28, 49.22, 49.14, 49.08, 49.00, 48.94, 48.86, 48.79, 48.72, 48.65, 48.57, 48.51, 48.37, 42.71, 36.42, 30.94, 29.99, 29.75, 29.06, 27.83, 26.39, 25.94, 25.82, 25.19, 21.21, 21.12, 19.39, 14.17 ppm; **HRMS** (ESI$^+$): calcd. for $C_{79}H_{98}N_{16}O_{13}^{2+}$ $[M+2H]^{2+}$ 739.3744, found 739.3725;

LC-ESI(+)-MS: column: Agilent Poroshell 120 EC-C18 (2.1 x 50 mm, 2.7 µm); mobile phase: MeCN (A)/ water (B) solvent mixture complemented with 0.1% formic acid; method: 98 → 40% B in 7 min; 0.5 mL/min, 40 °C; t_R = 5.57 min; m/z = $[M+2H]^{2+}$ = 739.5; Calcd. for $C_{79}H_{98}N_{16}O_{13}$ $[M+2H]^{2+}$ = 739.4.

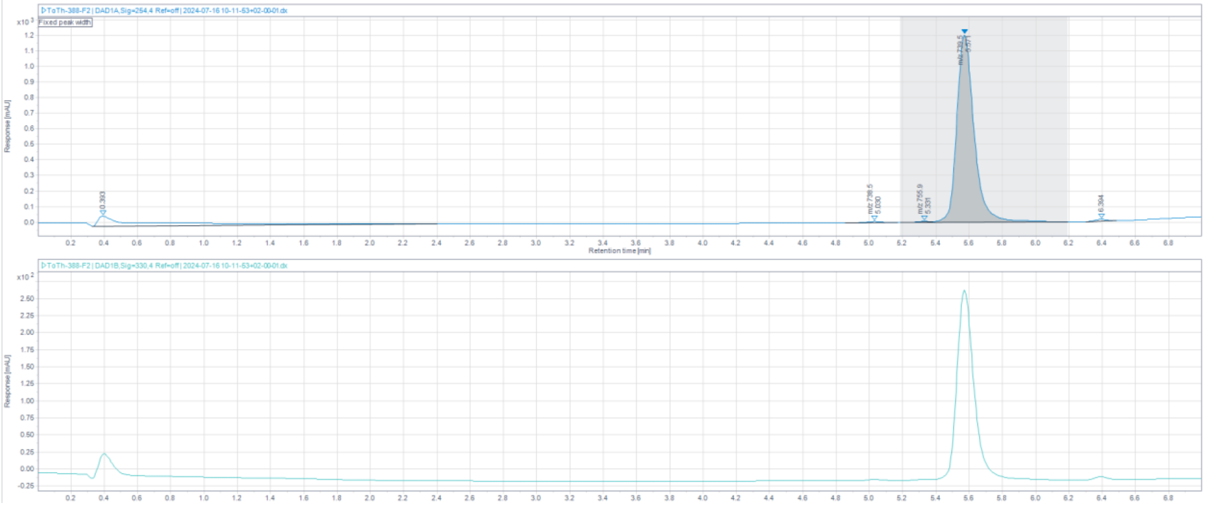

Figure S8: LC-UV chromatogram of **3c** at 254 nm and 330 nm.

4. Circular Dichroism (CD) Spectra of Diads **2** and **3**

CD spectra were recorded on a *Jasco* J-1500 spectrometer using a spectral bandwidth of 2 nm with a time constant of 4 s and a step resolution of 0.2 nm at 25 °C. A quartz cell with a path length of 2 mm was used with peptide solutions of 25 μM. All samples were freshly prepared and measured in methanol (UV grade) to adapt best to the experimental conditions of the ^{1}H-photo-CIDNP experiments. The spectra of Pro-6 to Pro-12 diads **2c**-**2e** and **3c**-**3e** shown in Figure S9 exhibit characteristics of a PPII helical secondary structure with negative Cotton effects at 205 nm and positive Cotton effects at 227 nm.[3] Diads containing less than six proline residues (**2a**, **2b**, **3a**), only show weak Cotton effects independent from their F3-substitution. This indicates a high amount of different isomers present, which do not contain an all-*trans* amide bond PPII structure. Overall, the CD spectra

show that the more proline residues are present within the diad linker, the more significant the PPII structure is pronounced (**2a** < **2b** < **2c** < **2d** < **2e** and **3a** < **3c** < **3d**).

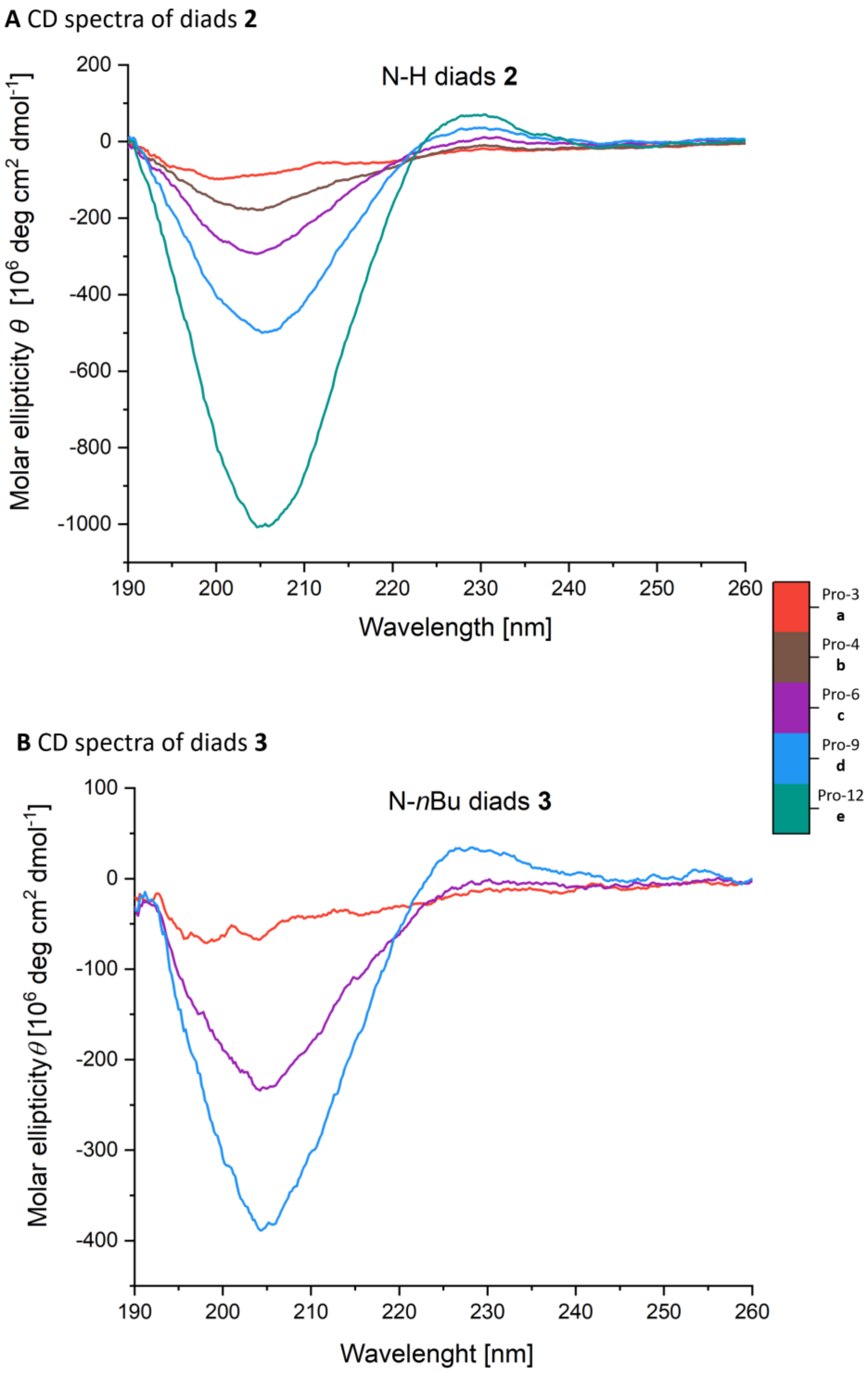

Figure S9: CD spectra of diads **2** and **3** measured in 25 µM MeOH-d_4 solutions with its color and numeration index on the right. **A**: Superimposed CD spectra of photo-CIDNP diads **2**. **B**: Superimposed CD spectra of photo-CIDNP diads **3**.

5. NMR-Analysis of Diads **2** and **3**

5.1 NMR-Assignment of Diad **2e**

As a representative diad for an NMR assignment, we used F3-*NH* Pro-12 diad **2e** since, with variation in the number of prolines, only the signal intensity changes when compared to the other diads (Table S1). All diads show a similar number of signals in the flavin part attributing to different rotamers, which manifests in shoulder peaks in the aromatic region and the aliphatic protons of F7’ and F8’.

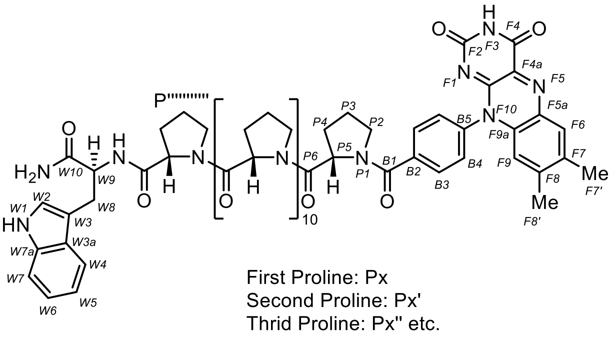

Table S1: NMR assignment of Pro-12 diad **2e**.

Atom	^{13}C-NMR (δ)	^{1}H-NMR (δ, m, *J*)	COSY	HMBC
F1	-	-	-	-
F2	-	-	-	-
F3	-	-	-	-
F4	-	-	-	-
F4a	-	-	-	-
F5	-	-	-	-
F5a	133.9	-		F9, F8’
F6	129.9	8.03, s	F7’	F9, F7’, F9a, F8
F7	136.5	-		F9, F7’, F8’
F7’	17.03	2.39, s	-	F6, F8’
				F9a, F9, F8, F7, F6, F8’
F8	147.0	-	-	F6, F7’, F8’
F8’	18.81	2.29, s	-	F9, F7’
				F5a, F9, F8, F7, F7’

F9	116.1	6.71, s	F8’	F7’, F8’, F9a, F5a, F7, F6, F8’
F9a	131.5	-	-	F9, F6, F7’
F10	-	-	-	-
F10a	-	-	-	-
B1	168.1	-	-	B3
B2	137.2	-	-	B4
B3	128.4	7.86, d, 8.1 Hz	B4	B1; B5
B4	127.1	7.56, d, 8.3 Hz	B3	B2
B5	136.5	-	-	B3
P1	-	-	-	-
P2	46.33	3.98, m	P2’, P2’’	P3, P5, P4
P3	23.57	2.24, m	-	P2’, P2’’, P5, P4’, P4’’
P4	27.38	2.06, m	-	P2’, P2’’, P5, P3, P5, P6
P5	57.47	4.80, m	-	P2’, P2’’, P4’’, P3, P4, P6
P6	169.2	-	-	P5, P4’’
W1	-	-	-	-
W2	122.0	7.03, s	-	W8’, W8’’, W3, W3a, W7a
W3	107.9	-	-	W9, W8’, W8’’, W2, W4
W3a	126.5	-	-	W8’, W8’’, W2, W4, W6, W7
W4	120.0	7.05, m	W5	W6, W3, W3a, W6
W5	110.0	7.27, m	W4	W7
W6	116.7	7.57, m	W7	W4, W3a, W7a
W7	117.5	6.99, m	W6	W3a, W5
W7a	135.8	-	-	W2, W6
W8	25.62	H’: 3.20, d, 6.0 Hz	W8’’ W8’	W9, W10, W3, W3a, W2

		H'': 3.29, d, 6.3 Hz		
W9	52.69	4.57, t, 6.1 Hz	-	W8', W8'', W10, W8, W3
W10	174.2	-	-	W9, W8', W8''

5.2 Conformational Analysis of **2** and **3** by 2D-TOCSY and 2D-NOESY/ROESY-NMR Spectroscopy

The ratio of the all-*trans* PPII structure to *cis*-amide isomers (minor products) in MeOH-d_4 of diads **2** and **3** was determined by superimposing the 2D-TOCSY and ROESY/NOESY-NMR spectra.[4] All TOCSY, NOESY, and ROESY spectra were recorded on a Bruker-Spectrospin 600 UltraShield NMR spectrometer (Bruker Corporation, Karlsruhe, Germany) with a magnetic field of 600 MHz and a Bruker Avance Neo 500 (500 MHz) equipped with a cryo probe in MeOH-d_4. The characterization was based on the intra-proline ^{1}H-TOCSY correlation of the P2 and P5 protons and the inter-proline ^{1}H-NOESY/ROESY correlation of the adjacent P2 and P5 protons (Figure S10). The diastereotopic nature of the P2 protons gives rise to two different NMR signals for the P2 position, which results in a hard-to-distinguish signal environment. By using the correlations with the more straightforwardly assigned P5 protons, which are shown on the x-axis of the following 2D-TOCSY-and NOESY/ROESY-NMR spectra, more detailed conclusions of the spectra can be drawn. 2D-ROESY/NOESY NMR spectra (green) showed P5-H-P2'-H doublets which were not superimposing with the doublets of P5-H-P2-H in the TOCSY spectra (blue), indicating that the ROE/NOE interactions stem from adjacent proline moieties (Figure S10).

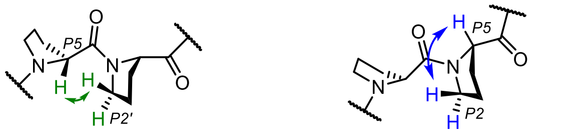

***inter*-Proline interaction**
NOESY/ROESY: *P5*-H - *P2'*-H

intra-Proline interaction
TOCSY: *P5*-H - *P2*-H

Figure S*10*: Intermolecular 2D NOESY/ROESY-NMR interaction of P5-H-P2'-H (left, green) and intramolecular TOCSY interaction P5-H-P2-H (right, blue) of the polyproline linker.

Beginning with the *N*-terminal proline Pro, there should be no TOCSY doublet lining up with a ROESY doublet since this is the first proline unit (no (n-1) proline present). The TOCSY doublet at 4.88 ppm indeed showed no lined-up ROESY doublet, indicating it as the *N*-terminal proline. Based on a study from *Mayer et. al.*[4] horizontal TOCSY/ROESY pairs indicating $(P5\text{-}H)_n\text{-}(P2\text{-}H)_{n+1}$ interactions of neighboring proline rings (n = number of proline ring) and vertical peak alignments showing $(P5\text{-}H)_n\text{-}(P2\text{-}H)_n$ interactions (Figure S11) could be detected. The following section examplifies the general procedure of determining the ratio of the amide conformers with 2D TOCSY- and NOESY/ROESY-NMR by employing the Pro-4-NH diad **2b**.

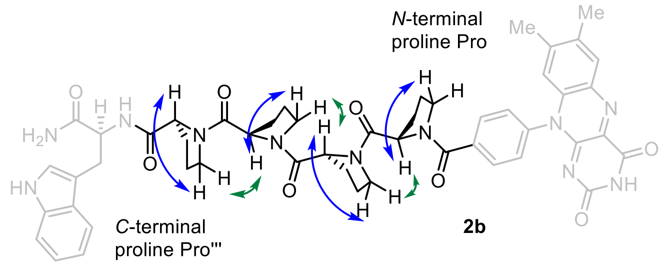

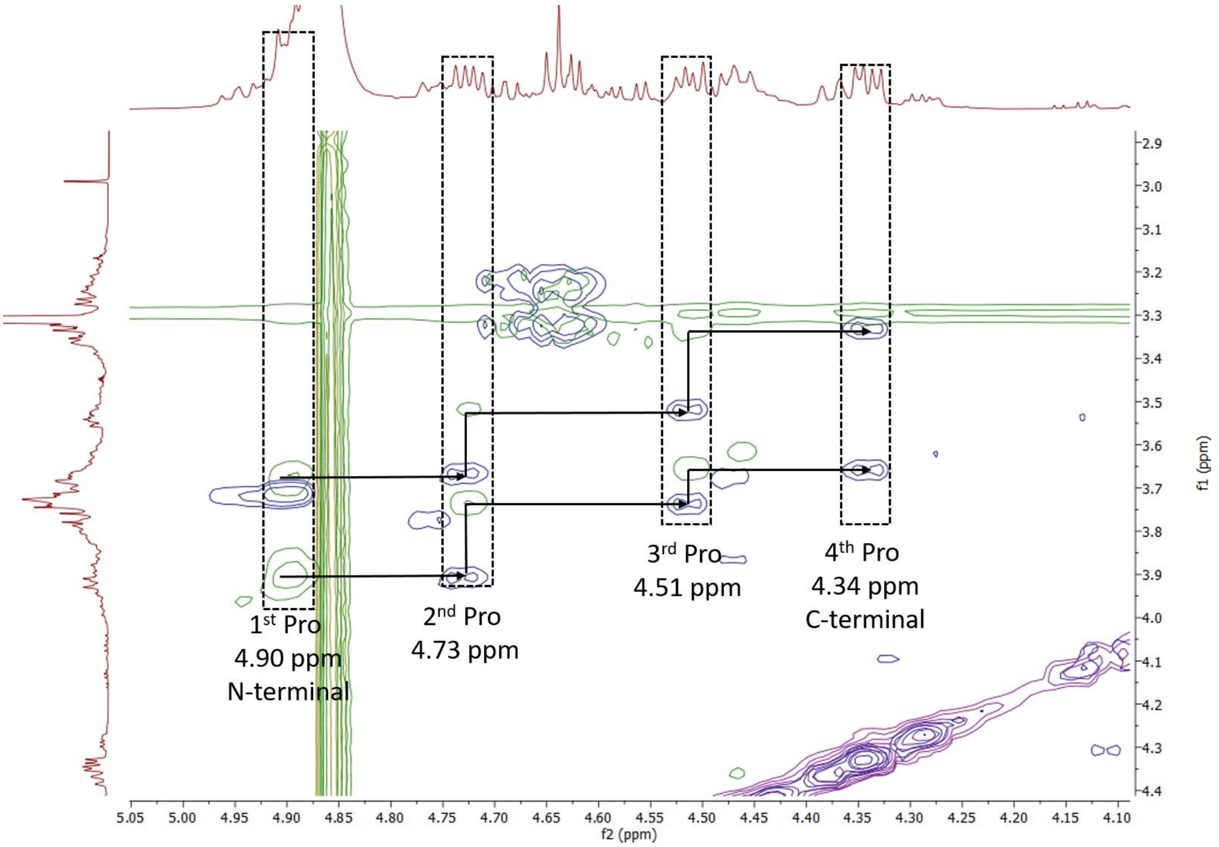

Figure S11: Superimposed ^1H-^1H-ROESY (green) and ^1H-^1H-TOCSY (blue) spectra for Pro-4-NH diad **2b** measured in MeOH-*d₄*. Horizontal black arrows indicate intermolecular interactions between adjacent prolines and vertical lines showing intermolecular interactions on the same resonance. Dashed boxes showing the assigned P5 ^1H-signals on the x-axis.

By increasing the signal intensity of the superimposed TOCSY-ROESY spectrum, additional TOCSY-ROESY pairs could be detected, hinting for *cis*-isomers disrupting the PPII structure (Figure S12). The NMR signals a, b, and c (red dashed boxes in Figure S12) correspond to the P5 protons of three different isomeric forms of **2b** located at the *C*-terminal proline Pro'''. Signal a corresponds to the PPII-helical isomer (all-*trans* amide bonds), while *P5*-H signals b and c are part of unassigned isomers containing *cis*-configured amide bonds. The signal resonating at 2.83 ppm can clearly be assigned as one of the diastereotopic P2-protons in Pro''' of isomer b (the black line in Figure S12).

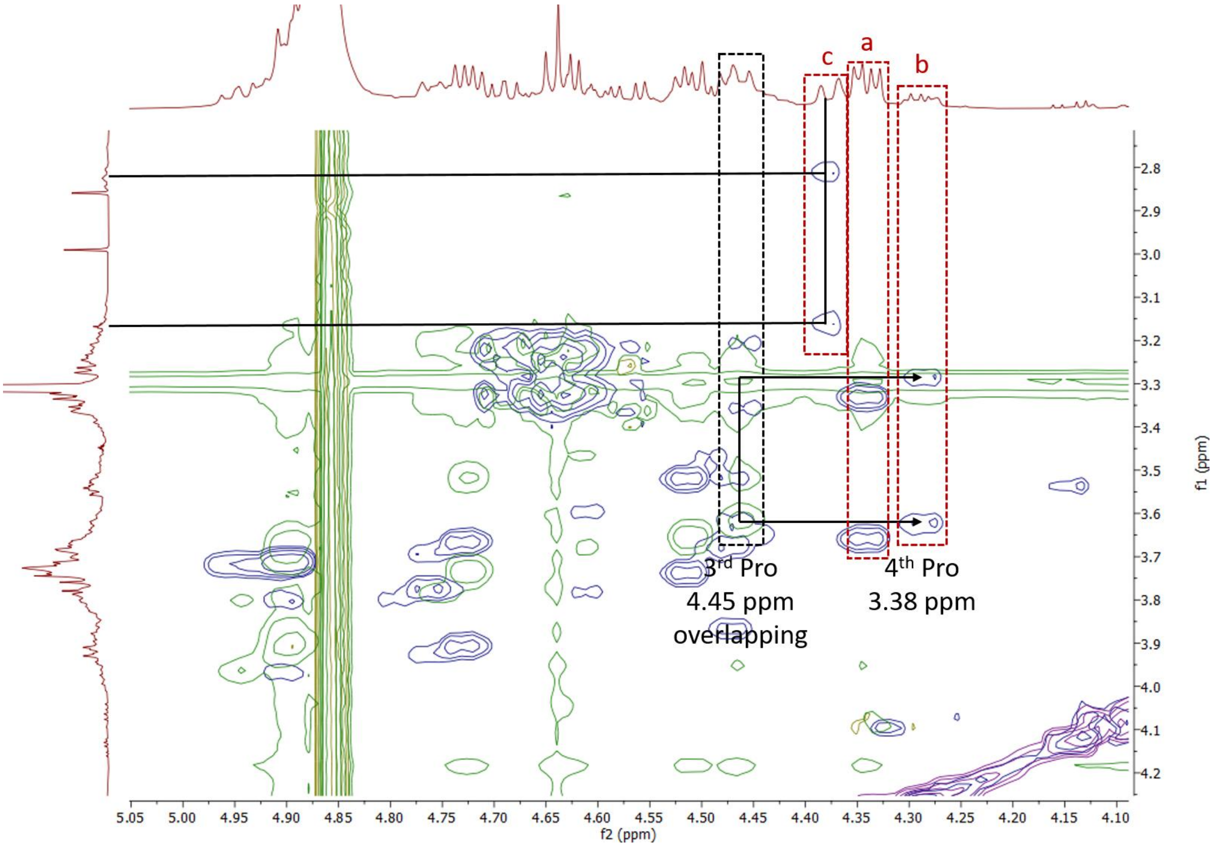

Figure S12: Zoomed-in intensities of the superimposed TOCSY-NOESY spectra of diad **2b** from Figure S11. **Isomer a** corresponds to the main species exhibiting a PPII helical structure (all-*trans* amide). **Isomer b** and **Isomer C** show at least one *cis*-amide conformation. The red dashed box showes the P5-proton of the C-terminal proline Pro, as there are only TOCSY (blue) doublets detectable due to the lack of another adjacent proline. The black dashed box highlights the P5-protons of the *N*-terminal proline Pro''' correlation with NOESY (green) doublets without the alignment with a TOCSY doublet as no (n-1)-proline residue is present.

We determined a 52:20:28 ratio of isomers a:b:c for diad **2b** by integrating the signals of the P5 protons (Figure S13). Therefore, the main isomer corresponds to the PPII conformer of **2b** a result in accordance with our findings in CD-spectroscopy (cf. Figure S9).

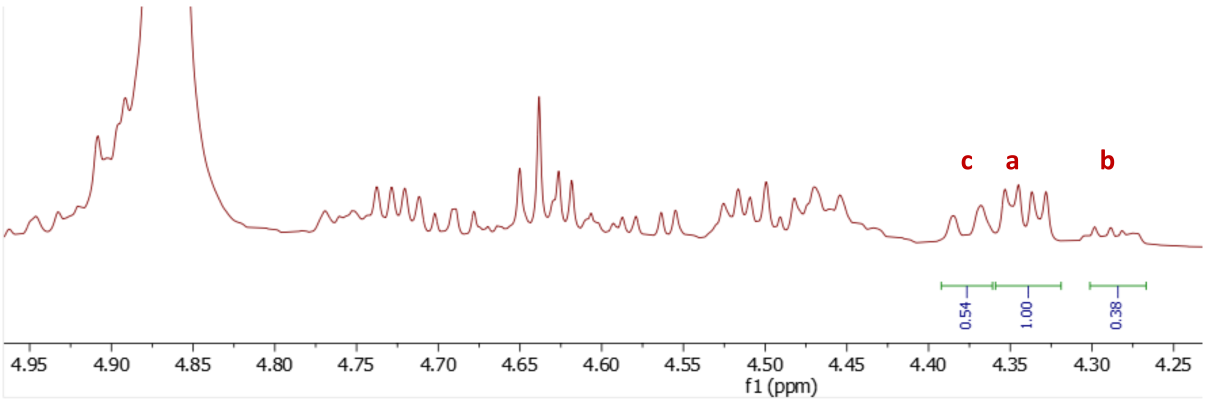

Figure S13: Integrals of the three different isomers a, b, and c in the ^{1}H-NMR spectrum of diad **2b**.

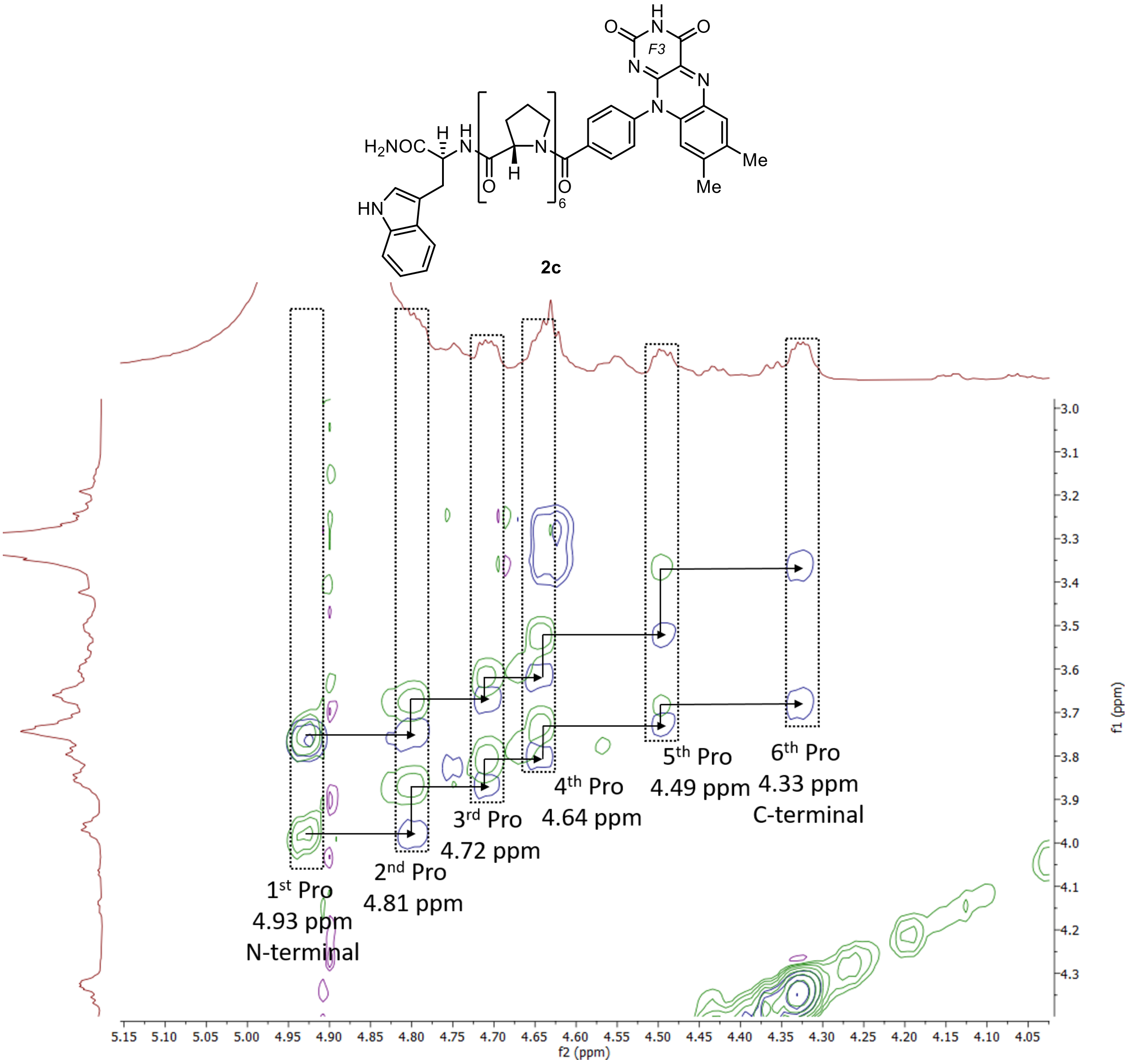

Figure S14: Superimposed ^{1}H-^{1}H-NOESY (green) and ^{1}H-^{1}H-TOCSY (blue) spectra of Pro-6-NH diad **2c** measured in MeOH-*d*$_4$. Horizontal black arrows indicate intermolecular interactions between adjacent prolines and vertical lines showing intermolecular interactions at the same resonance. Dashed boxes showing the assigned P5 ^{1}H-signals.

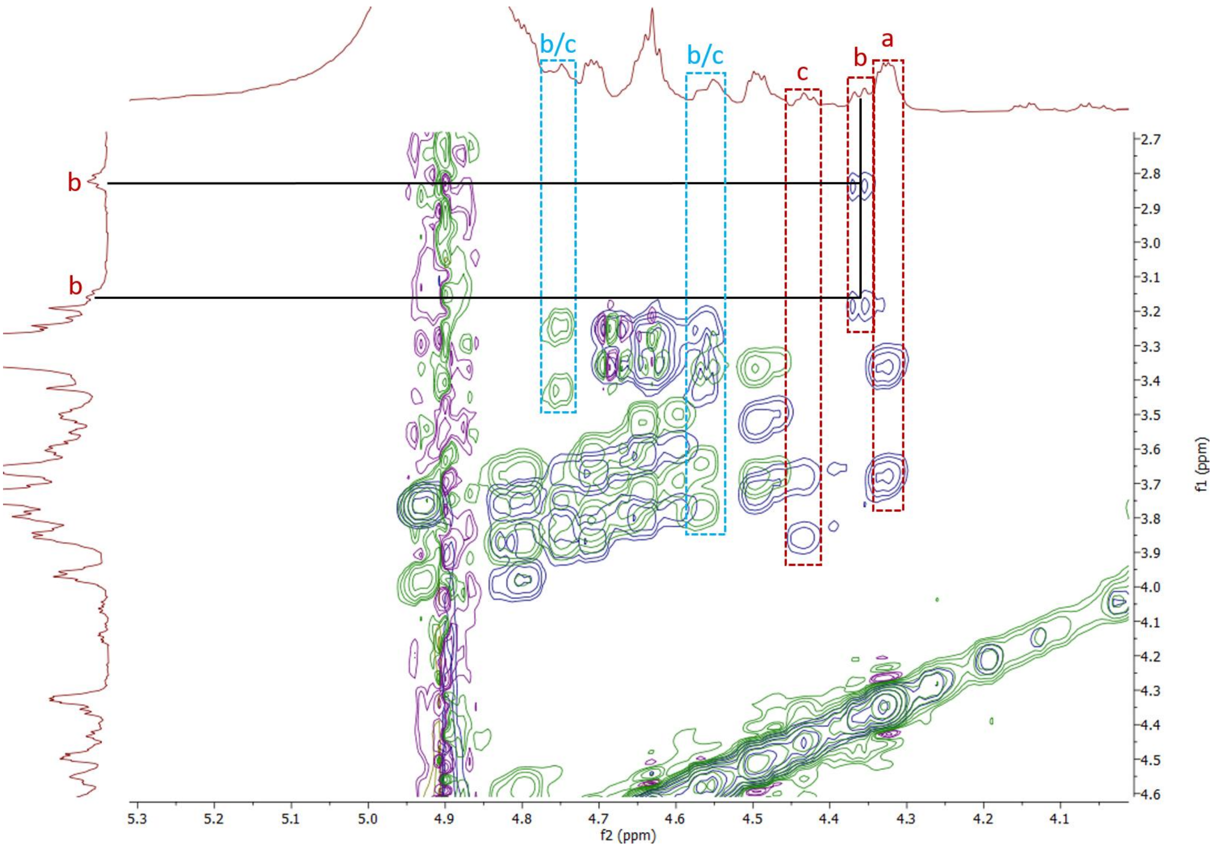

Figure S15: Zoomed-in intensities of the superimposed TOCSY-NOESY spectra of diad **2c** from Figure S14. **Isomer a** corresponds to the main species exhibiting a PPII helical structure (all-*trans* amide). **Isomer b** and **Isomer C** show at least one *cis*-amide conformation. The red dashed box showes the P5-proton of the C-terminal proline Pro, as there are only TOCSY (blue) doublets detectable due to the lack of another adjacent proline. The blue dashed box highlights the P5-protons of the *N*-terminal proline Pro’’’’’ correlation with NOESY (green) doublets without the alignment with a TOCSY doublet as no (n-1)-proline residue is present.

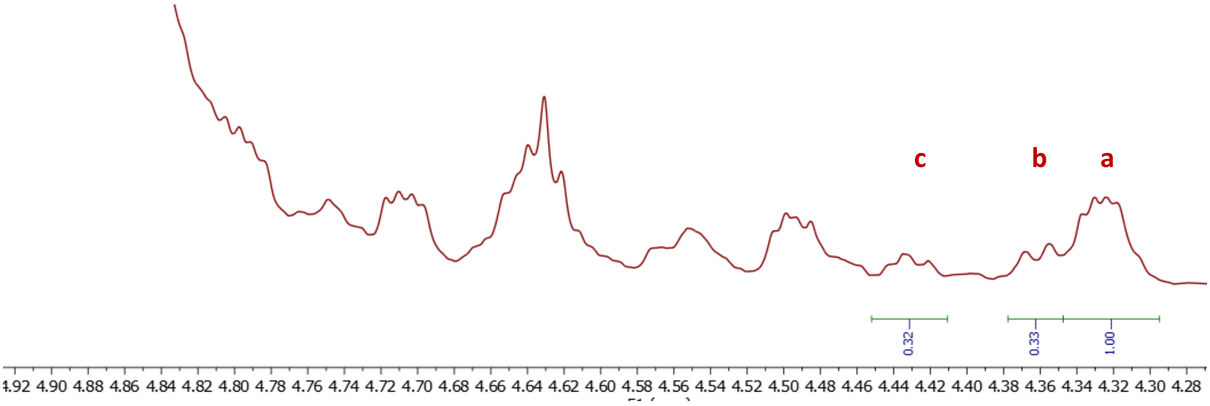

Figure S16: Integrals of the three different isomers a, b, and c in the ^{1}H-NMR spectrum of diad **2c**.

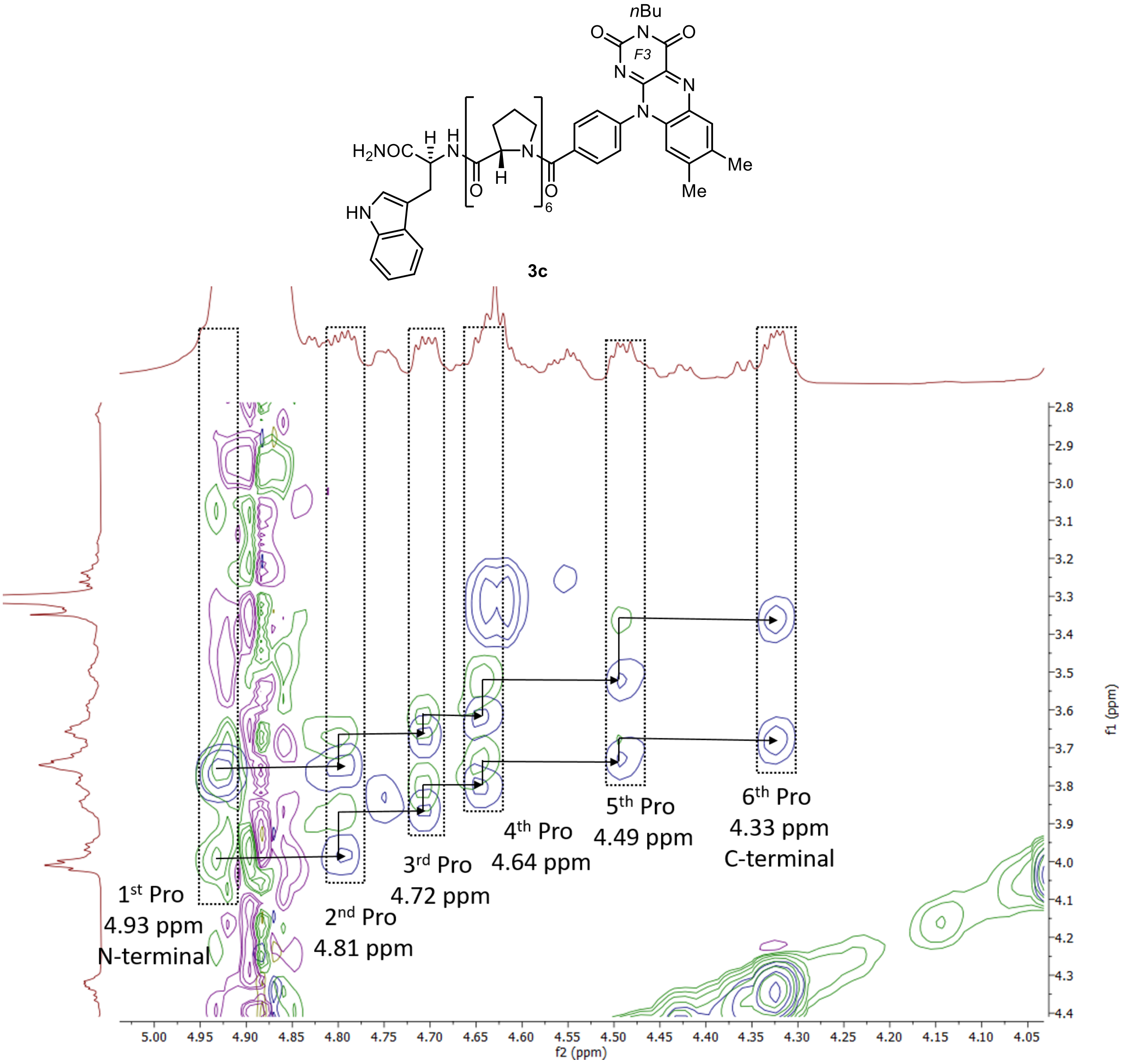

Figure S17: Superimposed ^{1}H-^{1}H-NOESY (green) and ^{1}H-^{1}H-TOCSY (blue) spectra of Pro-6-N*n*Bu diad **3c** measured in MeOH-*d*$_4$. Horizontal black arrows indicate intermolecular interactions between adjacent prolines and vertical lines showing intermolecular interactions on the same resonance. Dashed boxes showing the assigned P5 ^{1}H-signals.

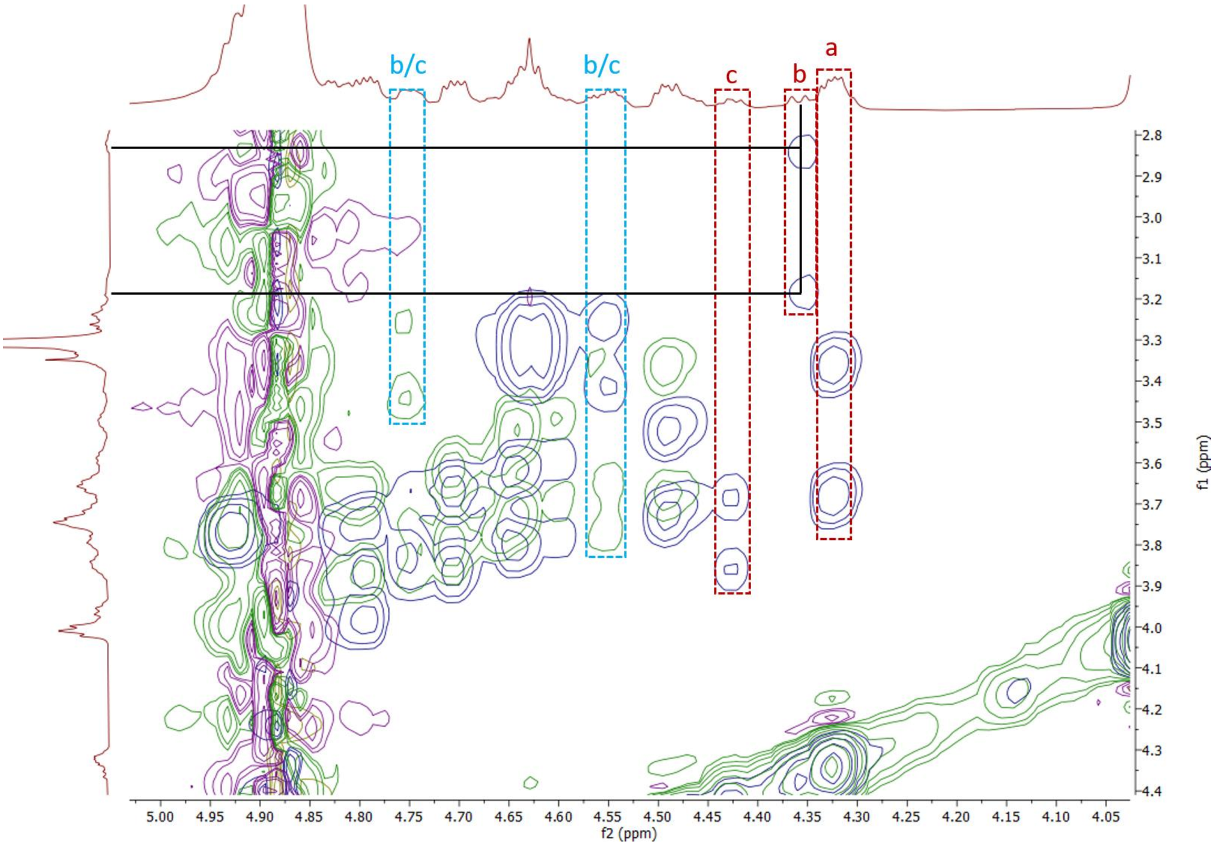

Figure S18: Zoomed-in intensities of the superimposed TOCSY-NOESY spectra of diad **3c** from Figure S17. **Isomer a** corresponds to the main species exhibiting a PPII helical structure (all-*trans* amide). **Isomer b** and **Isomer C** show at least one *cis*-amide conformation. The red dashed box showes the P5-proton of the C-terminal proline Pro, as there are only TOCSY (blue) doublets detectable due to the lack of another adjacent proline. The blue dashed box highlights the P5-protons of the *N*-terminal proline Pro'''''' correlation with NOESY (green) doublets without the alignment with a TOCSY doublet as no (n-1)-proline residue is present.

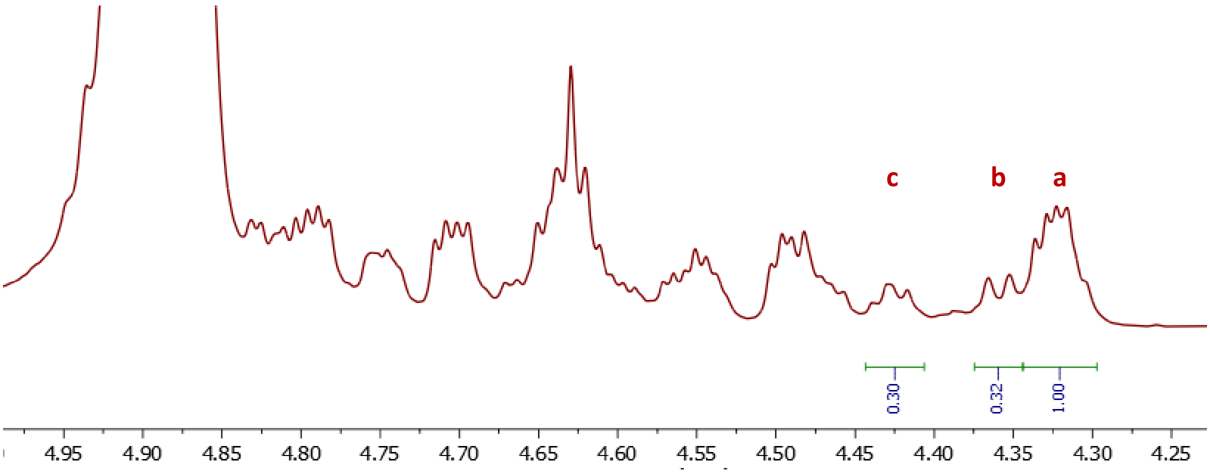

Figure S19: Integrals of the three different isomers a, b, and c in the ^{1}H-NMR spectrum of diad **3c**.

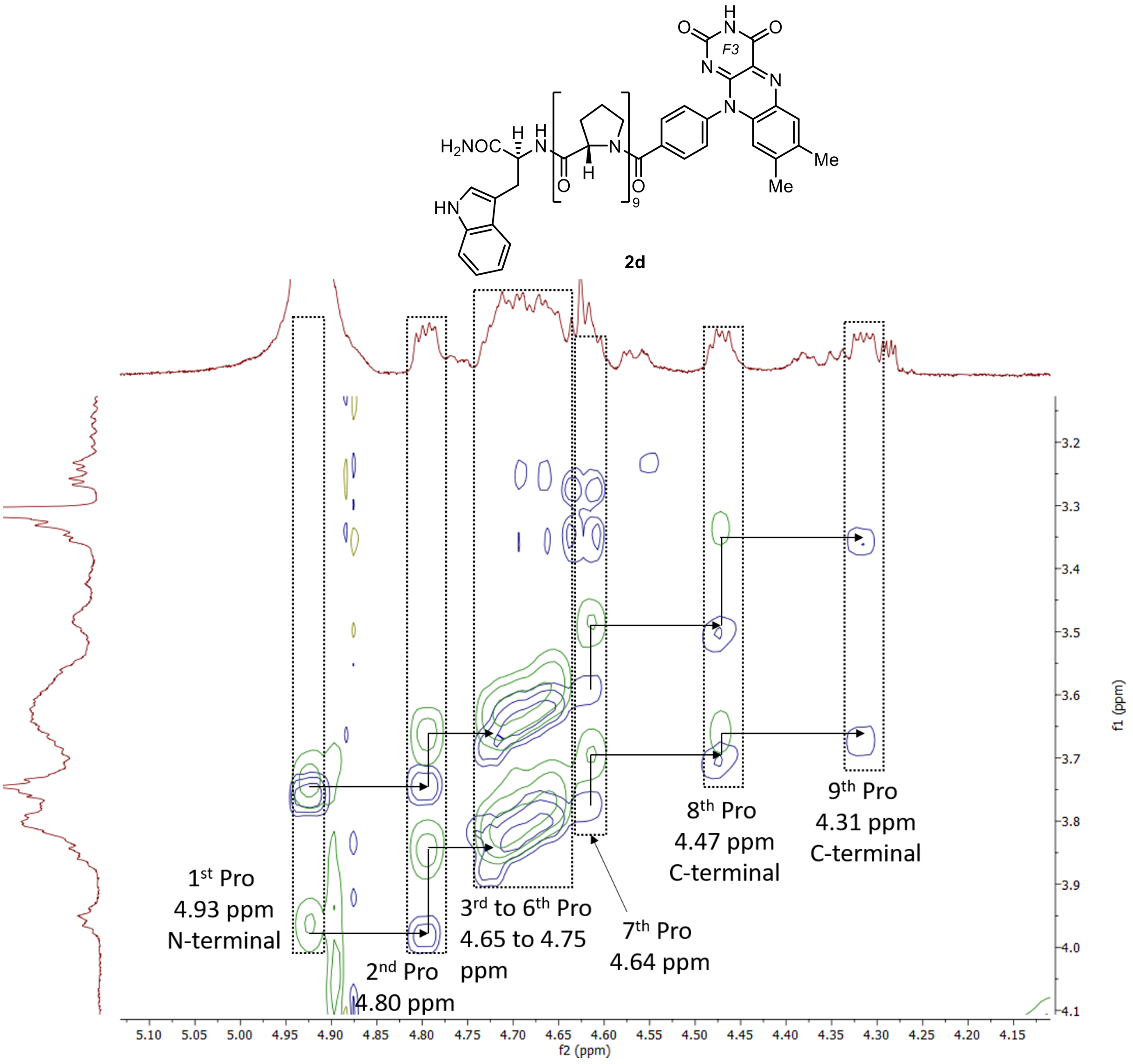

Figure S20: Superimposed ^1H-^1H-NOESY (green) and ^1H-^1H-TOCSY (blue) spectra of Pro-9-NH diad **2d** measured in MeOH-d_4. Horizontal black arrows indicate intermolecular interactions between adjacent prolines and vertical lines showing intermolecular interactions on the same resonance. Dashed boxes showing the assigned P5 ^1H-signals.

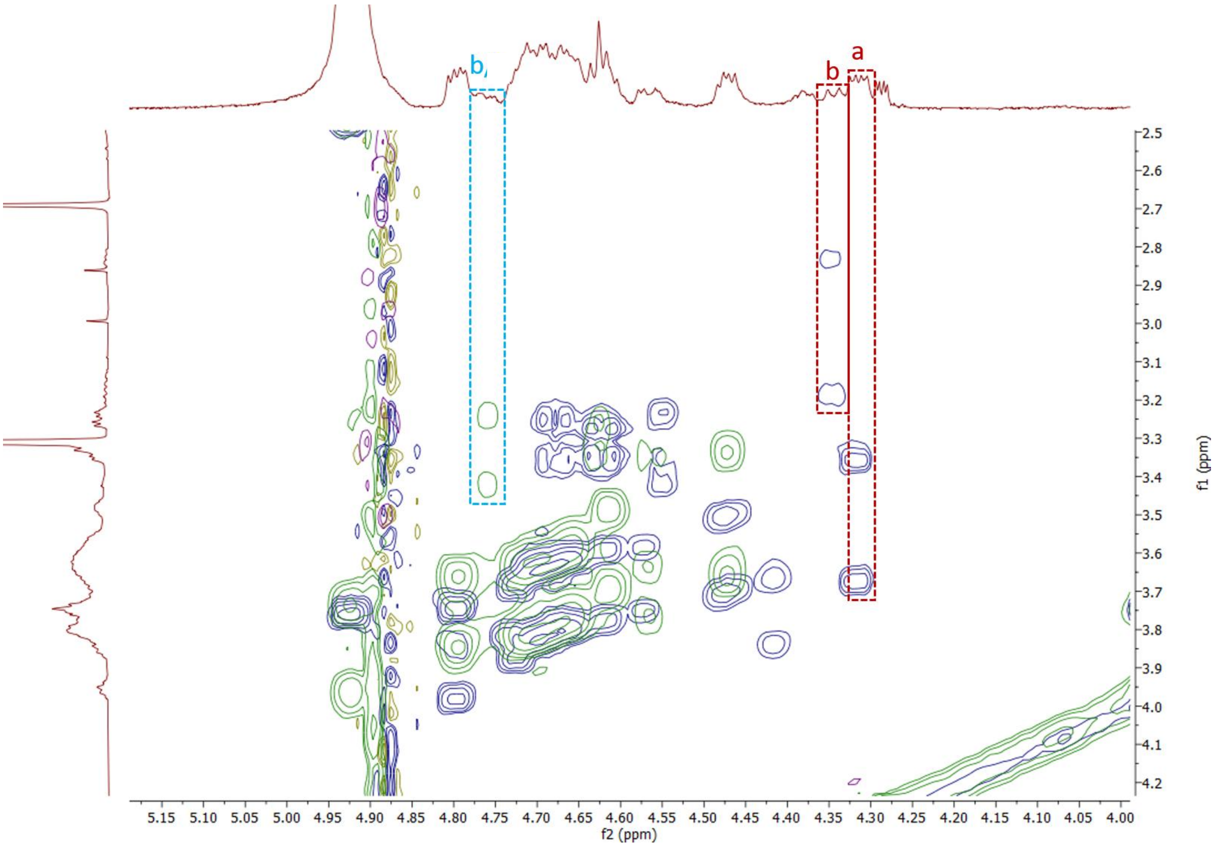

Figure S21: Zoomed-in intensities of the superimposed TOCSY-NOESY spectra of diad **2d** from Figure S20. **Isomer a** corresponds to the main species exhibiting a PPII helical structure (all-*trans* amide). **Isomer b** shows at least one *cis*-amide conformation. The red dashed box showes the P5-proton of the C-terminal proline Pro, as there are only TOCSY (blue) doublets detectable due to the lack of another adjacent proline. The blue dashed box highlights the P5-protons of the *N*-terminal proline Pro’’’’’’’ correlation with NOESY (green) doublets without the alignment with a TOCSY doublet as no (n-1)-proline residue is present.

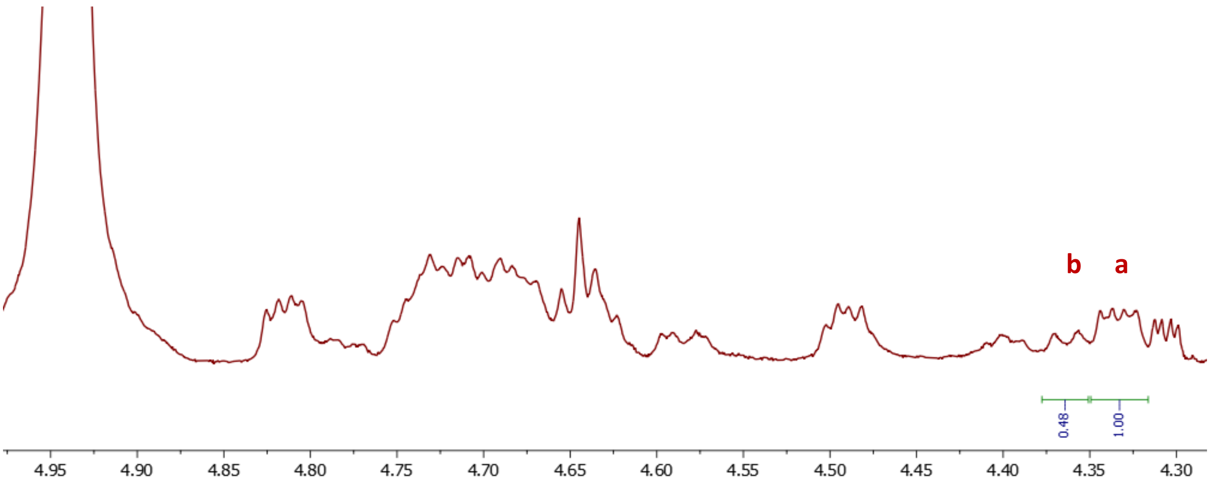

Figure S22: Integrals of the different isomers a and b in the ^{1}H-NMR spectrum of diad **2d**.

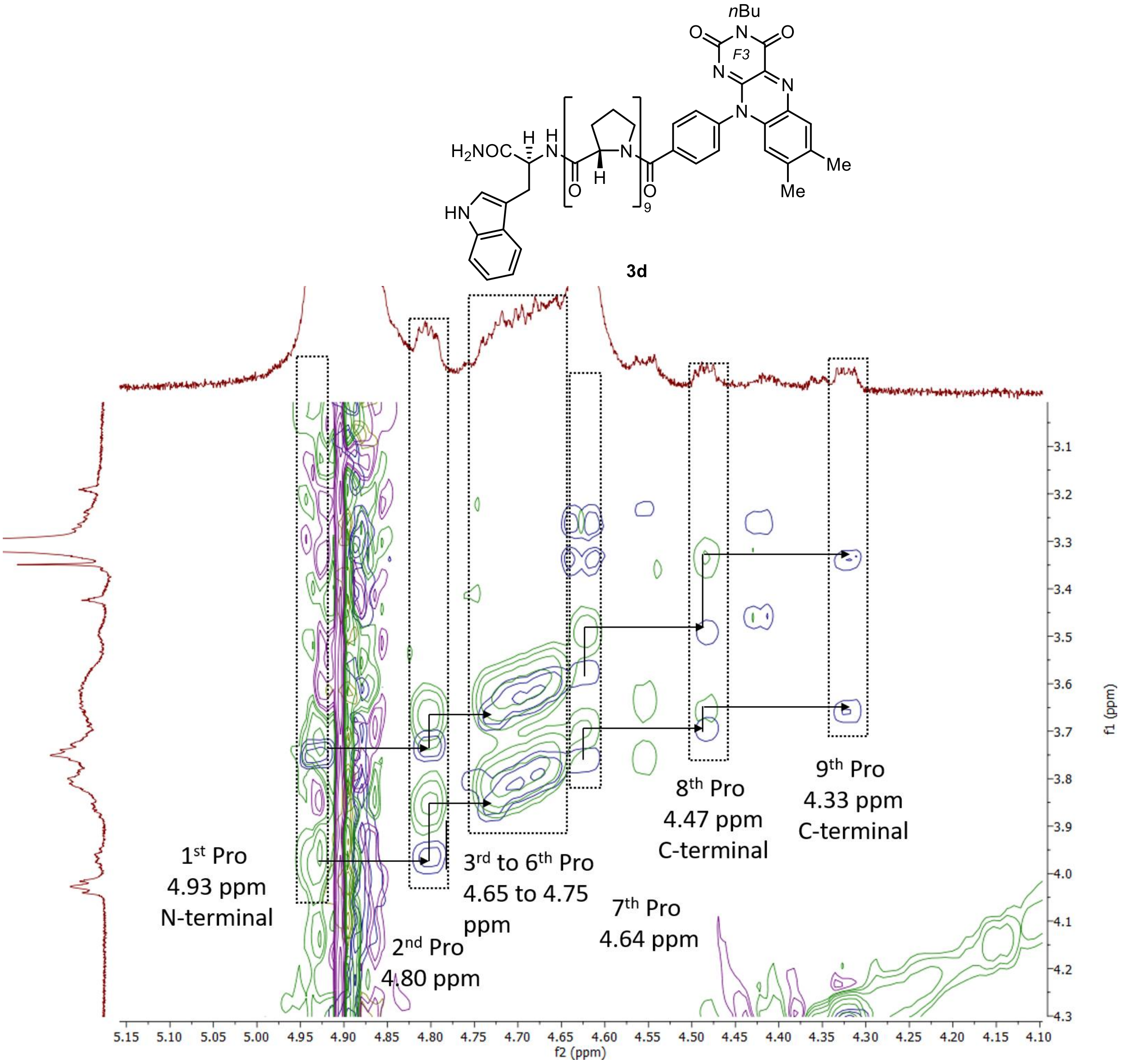

Figure S23: Superimposed ^{1}H-^{1}H-NOESY (green) and ^{1}H-^{1}H-TOCSY (blue) spectra of Pro-9-N*n*Bu diad **3d** measured in MeOH-*d$_4$*. Horizontal black arrows indicate intermolecular interactions between adjacent prolines and vertical lines showing intermolecular interactions on the same resonance. Dashed boxes showing the assigned P5 ^{1}H-signals.

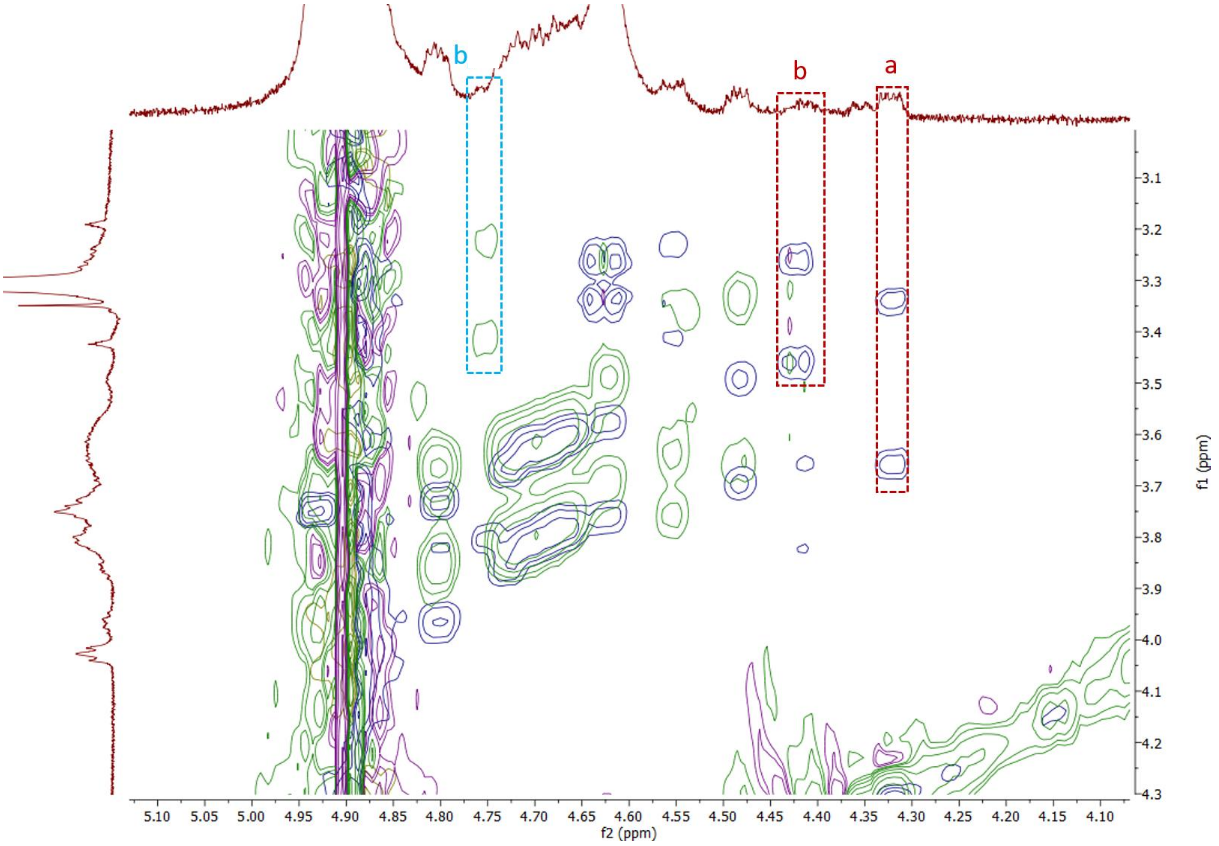

Figure S24: Zoomed-in intensities of the superimposed TOCSY-NOESY spectra of diad **3d** from Figure S23. **Isomer a** corresponds to the main species exhibiting a PPII helical structure (all-*trans* amide). **Isomer b** shows at least one *cis*-amide conformation. The red dashed box showes the P5-proton of the C-terminal proline Pro, as there are only TOCSY (blue) doublets detectable due to the lack of another adjacent proline. The blue dashed box highlights the P5-protons of the *N*-terminal proline Pro’’’’’’’’ correlation with NOESY (green) doublets without the alignment with a TOCSY doublet as no (n-1)-proline residue is present.

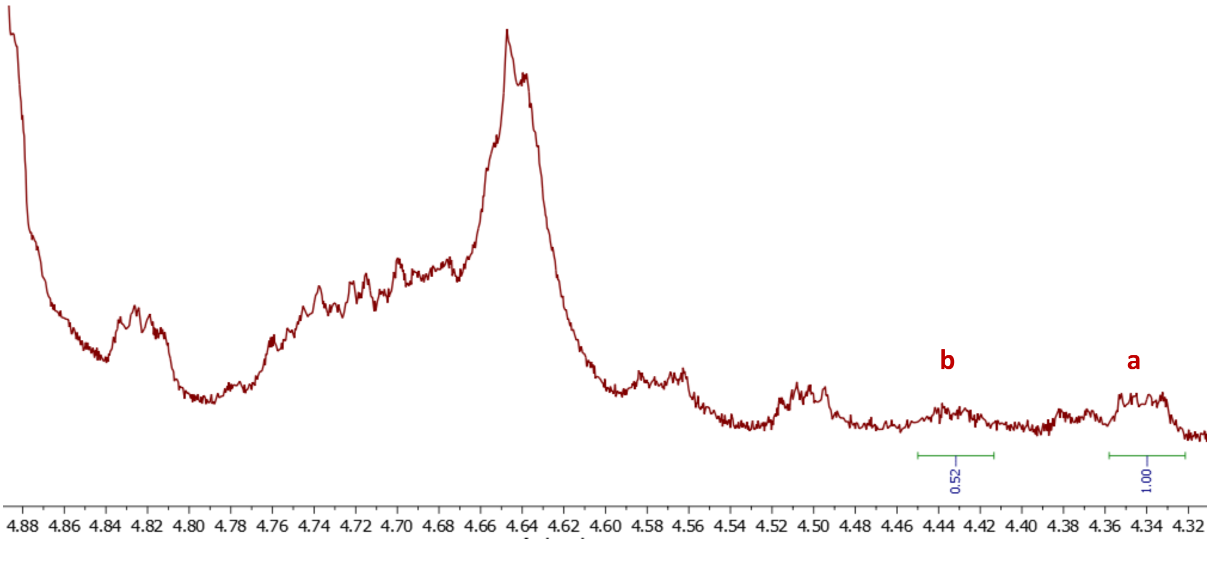

Figure S25: Integrals of the two different isomers a and b in the ^{1}H-NMR spectrum of diad **3d**.

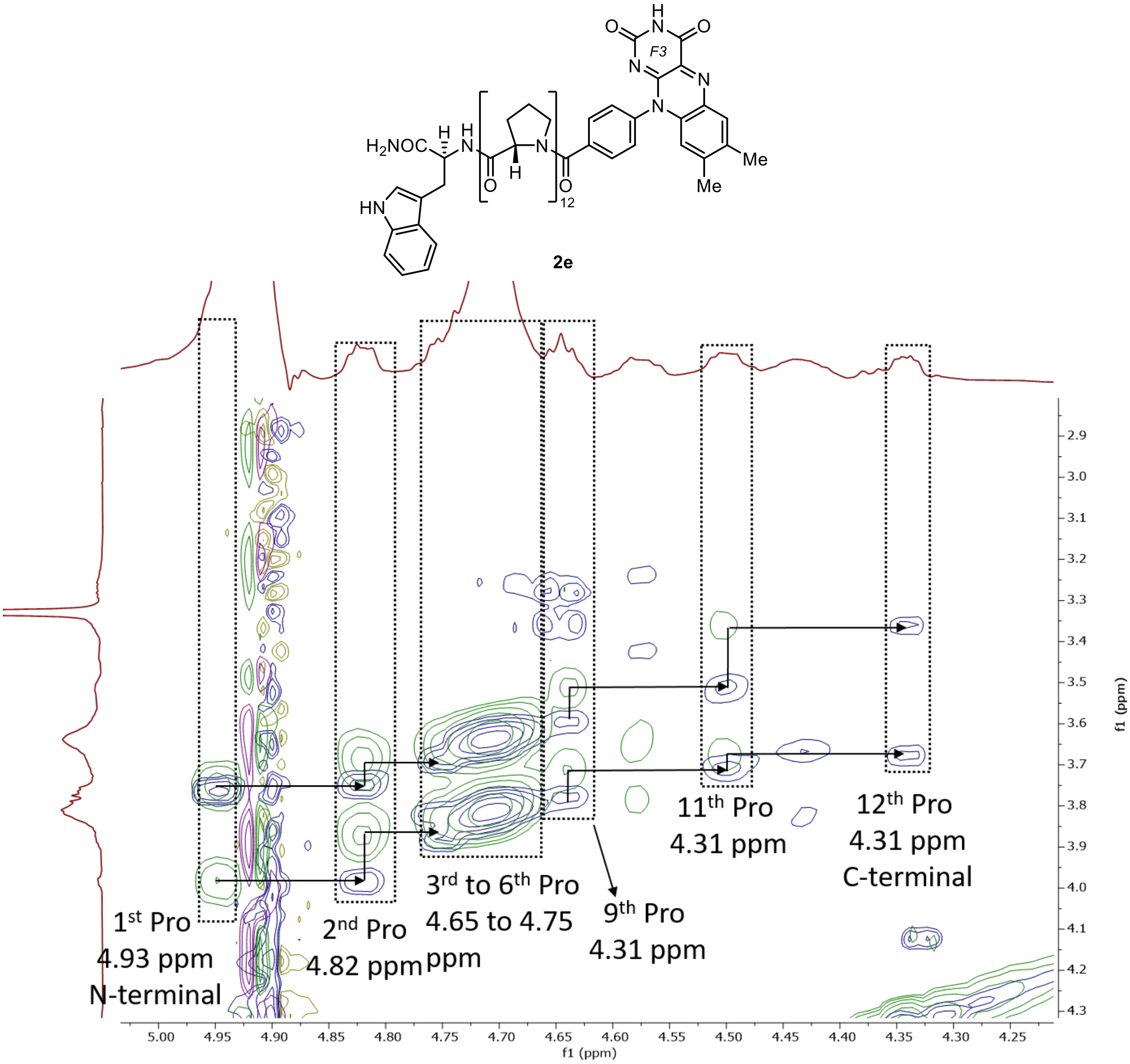

Figure S26: Superimposed ^{1}H-^{1}H-NOESY (green) and ^{1}H-^{1}H-TOCSY (blue) spectra of Pro-12-NH diad **2e** measured in MeOH-*d₄*. Horizontal black arrows indicate intermolecular interactions between adjacent prolines and vertical lines showing intermolecular interactions on the same resonance. Dashed boxes showing the assigned P5 ^{1}H-signals.

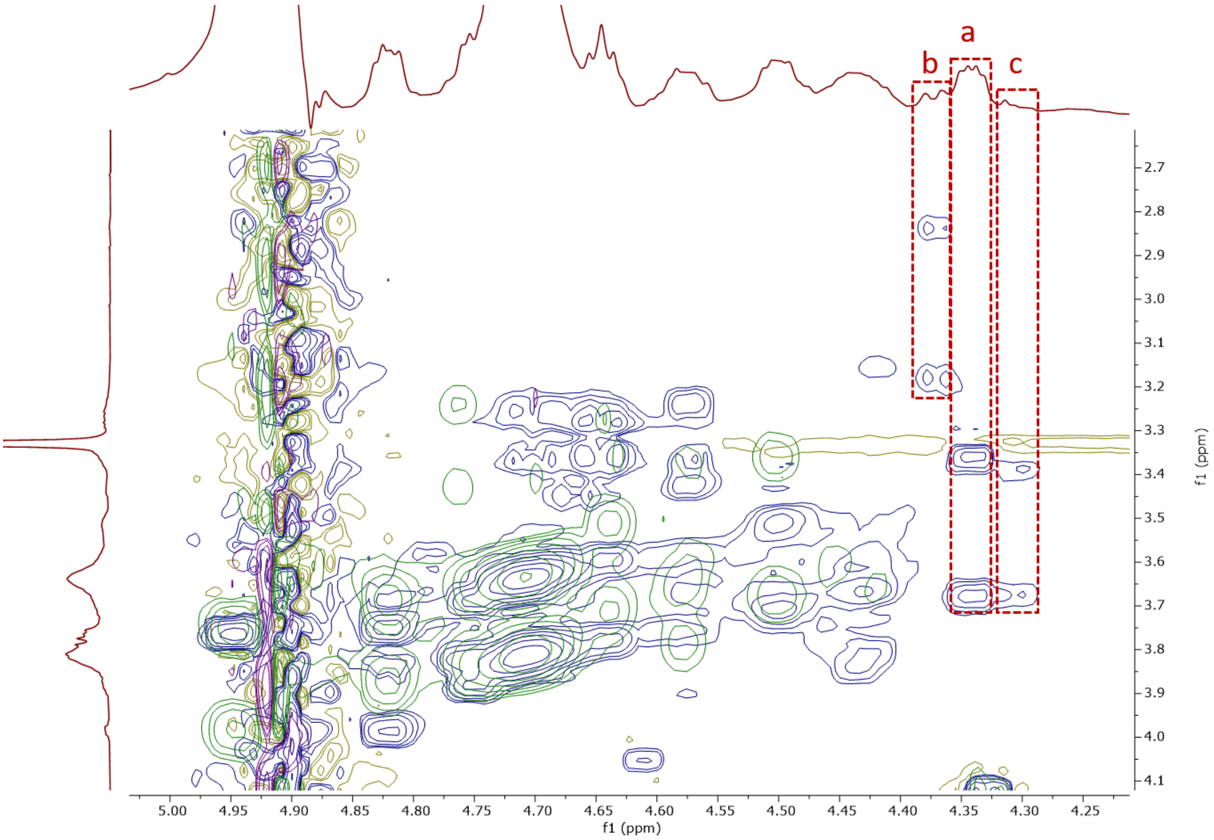

Figure S27: Zoomed-in intensities of the superimposed TOCSY-NOESY spectra of diad **2e** from Figure S2. **Isomer a** and **Isomer c** correspond to the main species exhibiting a PPII helical structure (all-*trans* amide). **Isomer b** and **Isomer c** show at least one *cis*-amide conformation. The red dashed box showes the P5-proton of the C-terminal proline Pro, as there are only TOCSY (blue) doublets detectable due to the lack of another adjacent proline.

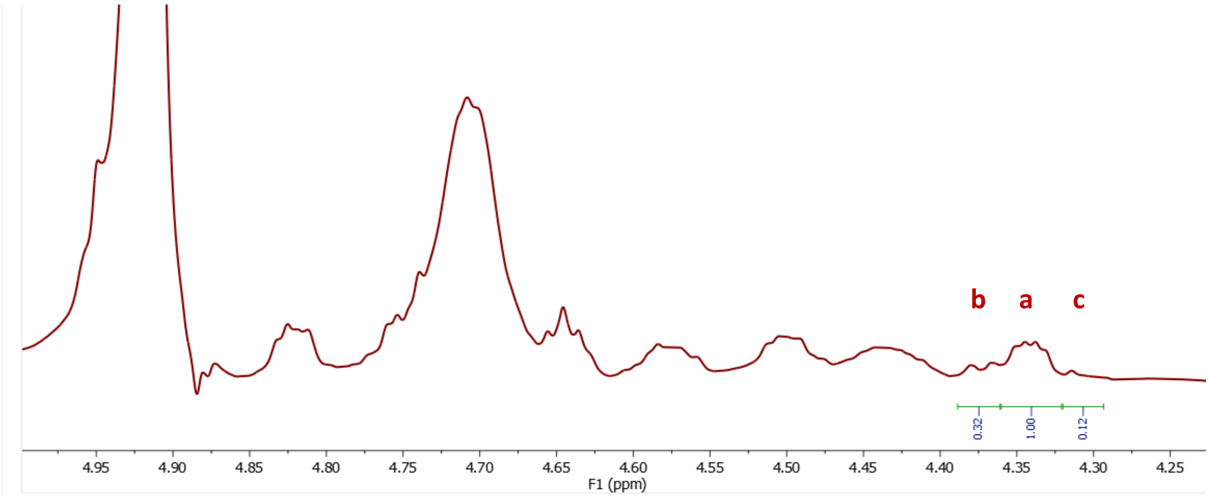

Figure S28: Integrals of the three different isomers a, b, and c in the ^{1}H-NMR spectrum of diad **2e**.

As already observed by CD spectroscopy (Figure S9), the Pro-3 diads **2a** and **3a** are highly conformationally flexible. Therefore, no through-space interactions could be detected by NOESY- or ROESY-NMR experiments corroborating the results obtained by CD.

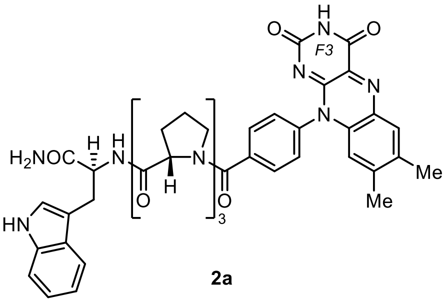

2a

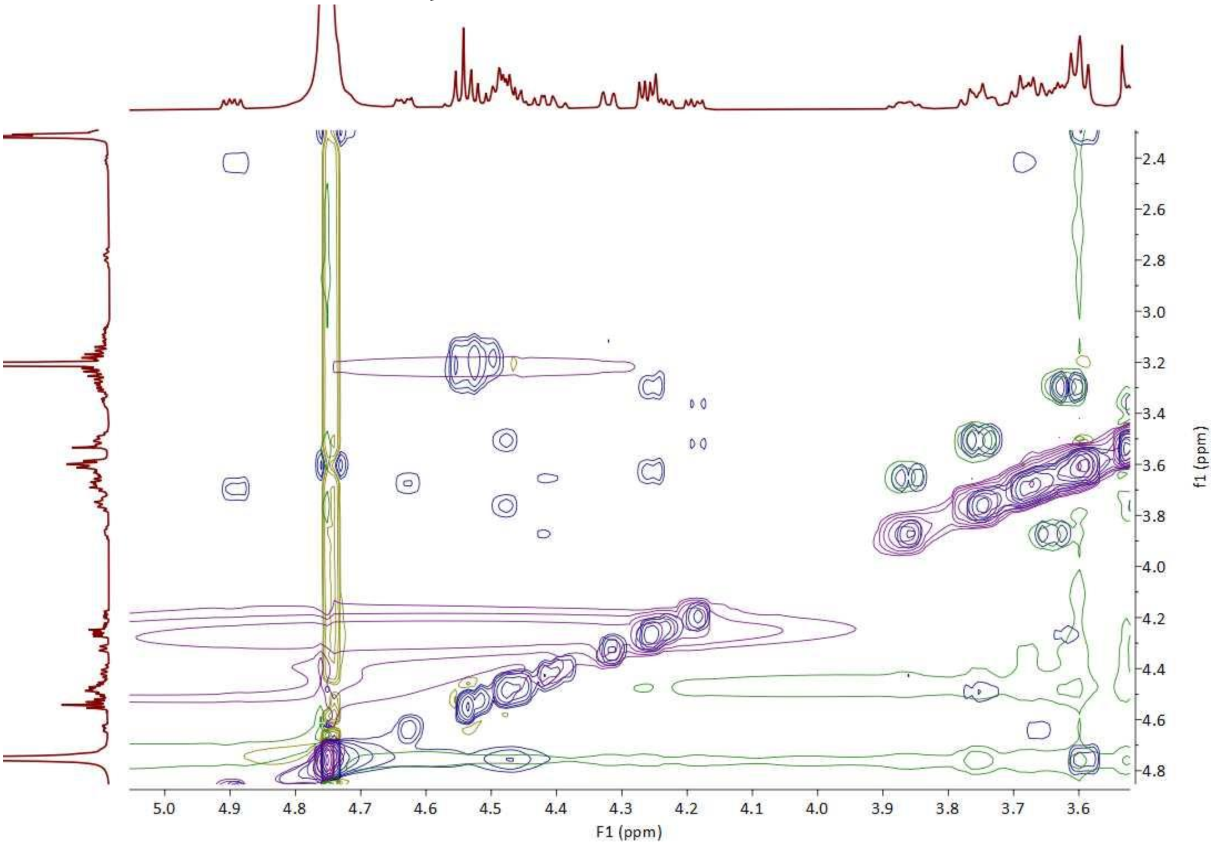

Figure S29: Superimposed ^1H-^1H-NOESY (green) and ^1H-^1H-TOCSY (blue) spectra of the conformationally highly flexible Pro-3-NH diad **2a** measured in MeOH-d_4.

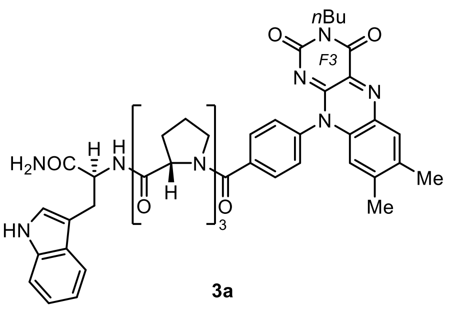

3a

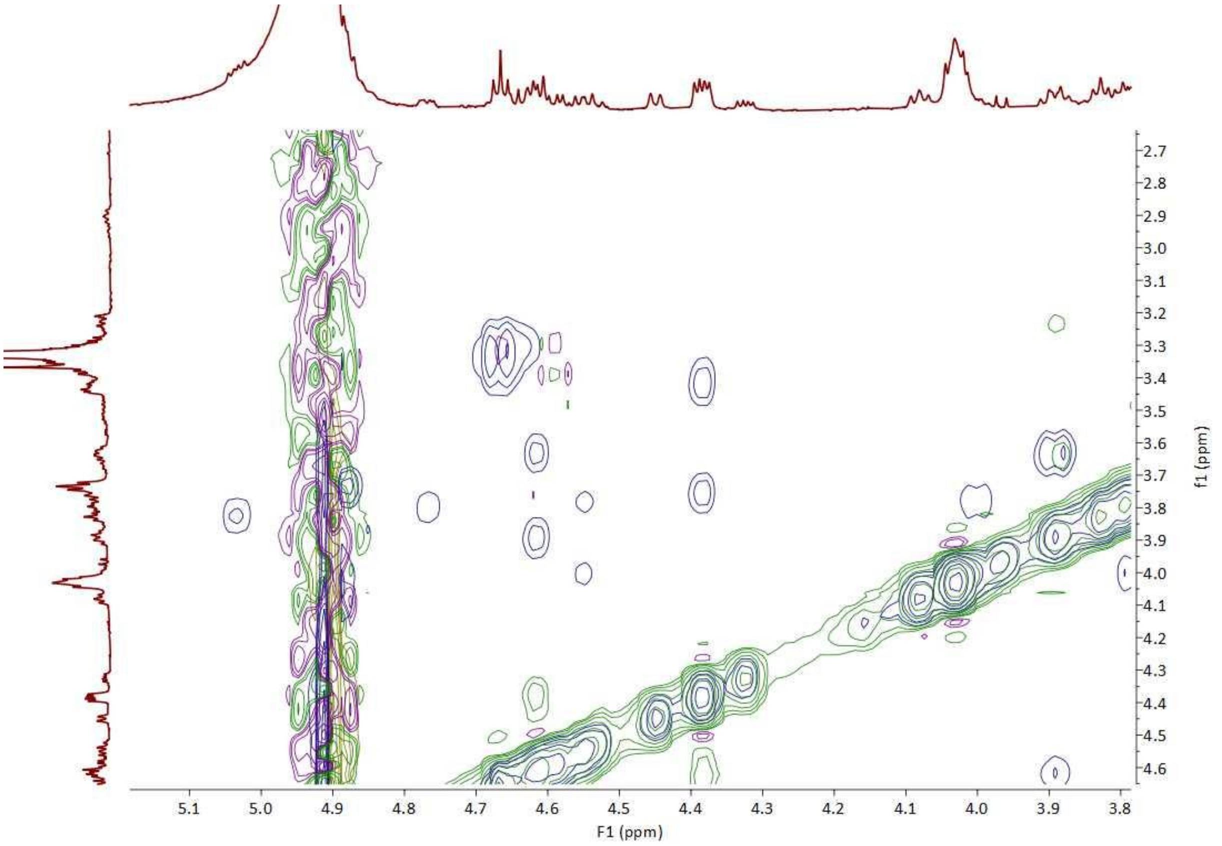

Figure S30: Superimposed ^{1}H-^{1}H-NOESY (green) and ^{1}H-^{1}H-TOCSY (blue) spectra of the conformationally highly flexible Pro-3-N*n*Bu diad **3a** measured in MeOH-*d*$_4$.

Altogether, the NMR investigations clearly show that the PPII helical conformation constitutes the dominant conformation in diads **2/3b - 2/3e**. Corresponding conformers showing at least one cis-amide bond become less populated with increasing linker length of the diads (Table S2). The conformational flexibility and preference for *trans*- or *cis*-amide conformations for the different diads is independent of the *F3* substituent.

Table S2: Ratio of PPII secondary structures in diads **2** and **3** depending on the Pro-linker length

Linker length	Isomers	PPII ratio [%]
Pro_3	-	n. d.
Pro_4	3	52
Pro_6	3	61
Pro_9	2	68
Pro_{12}	3	70

5.3 ^{1}H-NMR Spectra of Diads **2** and **3**

^{1}H-NMR spectrum recorded at 600 MHz

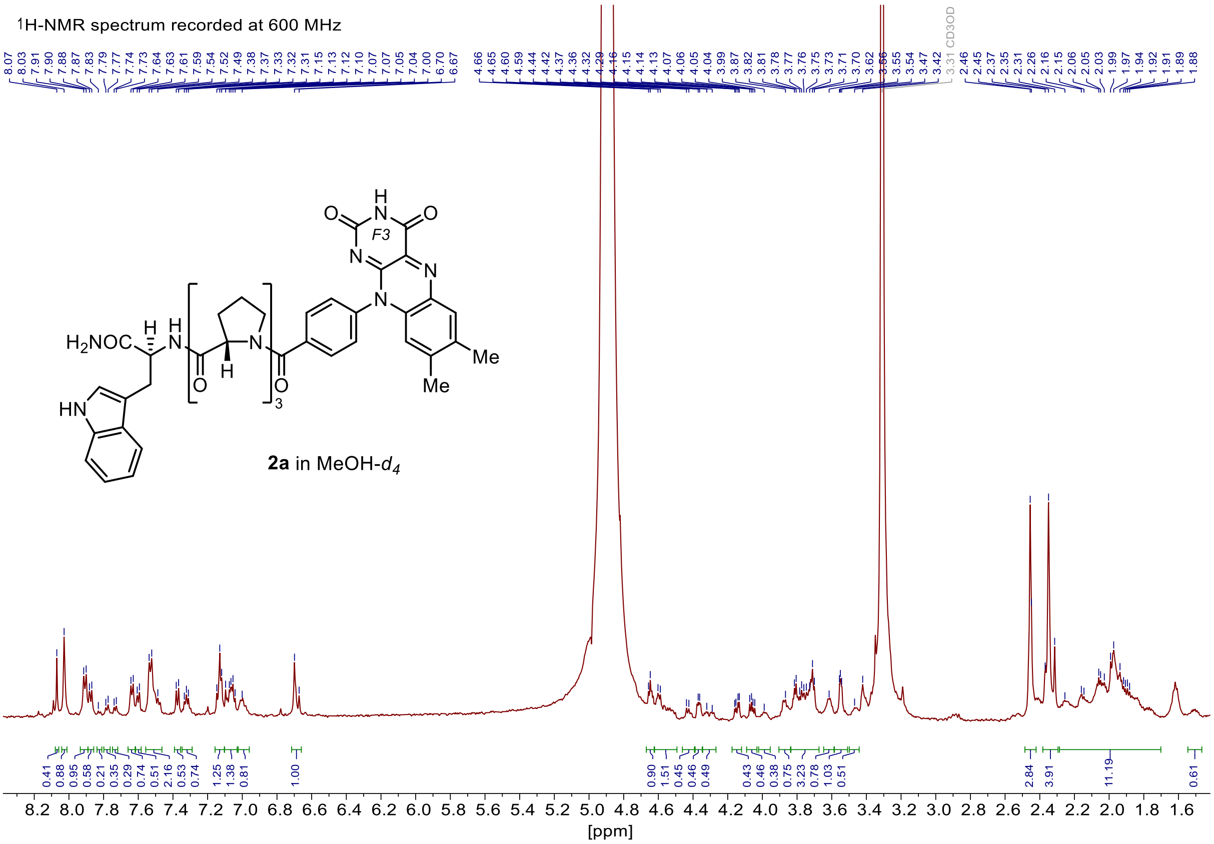

^{13}C-NMR spectrum recorded at 151 MHz

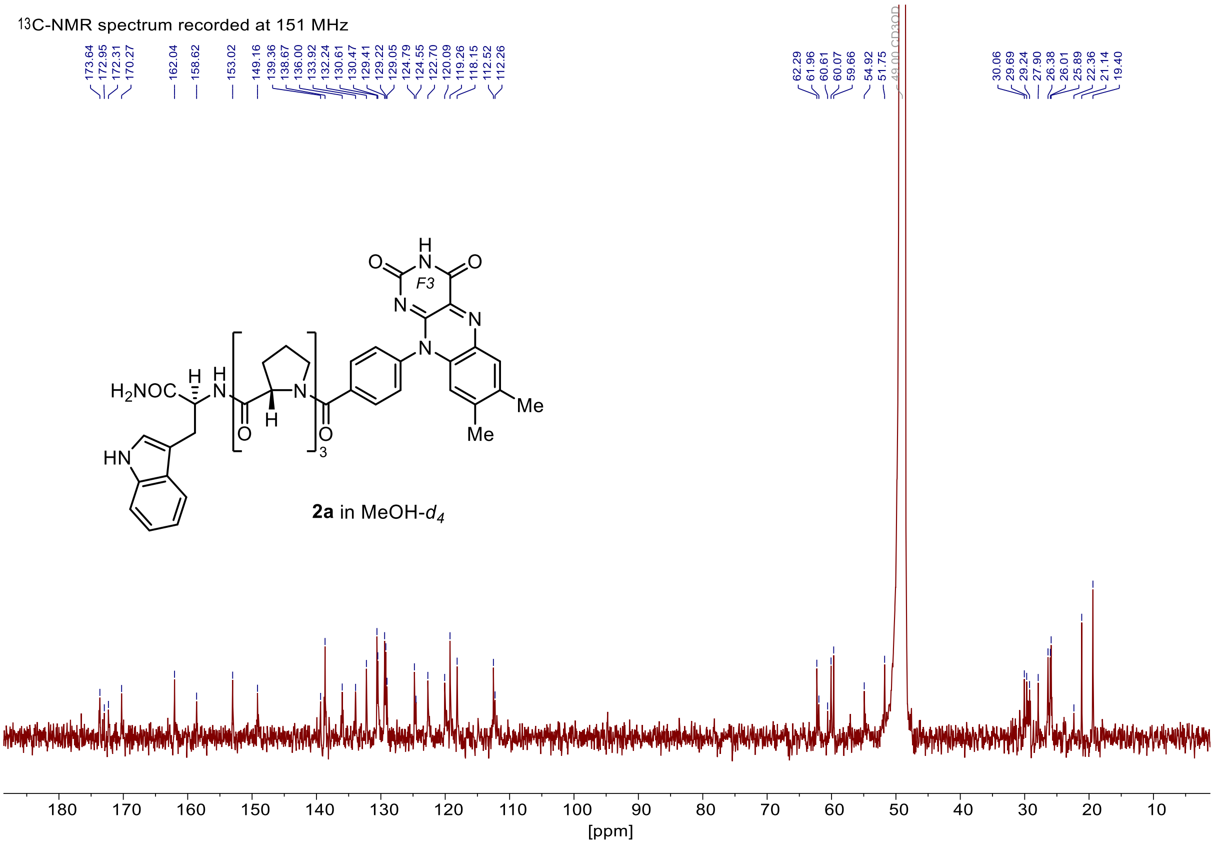

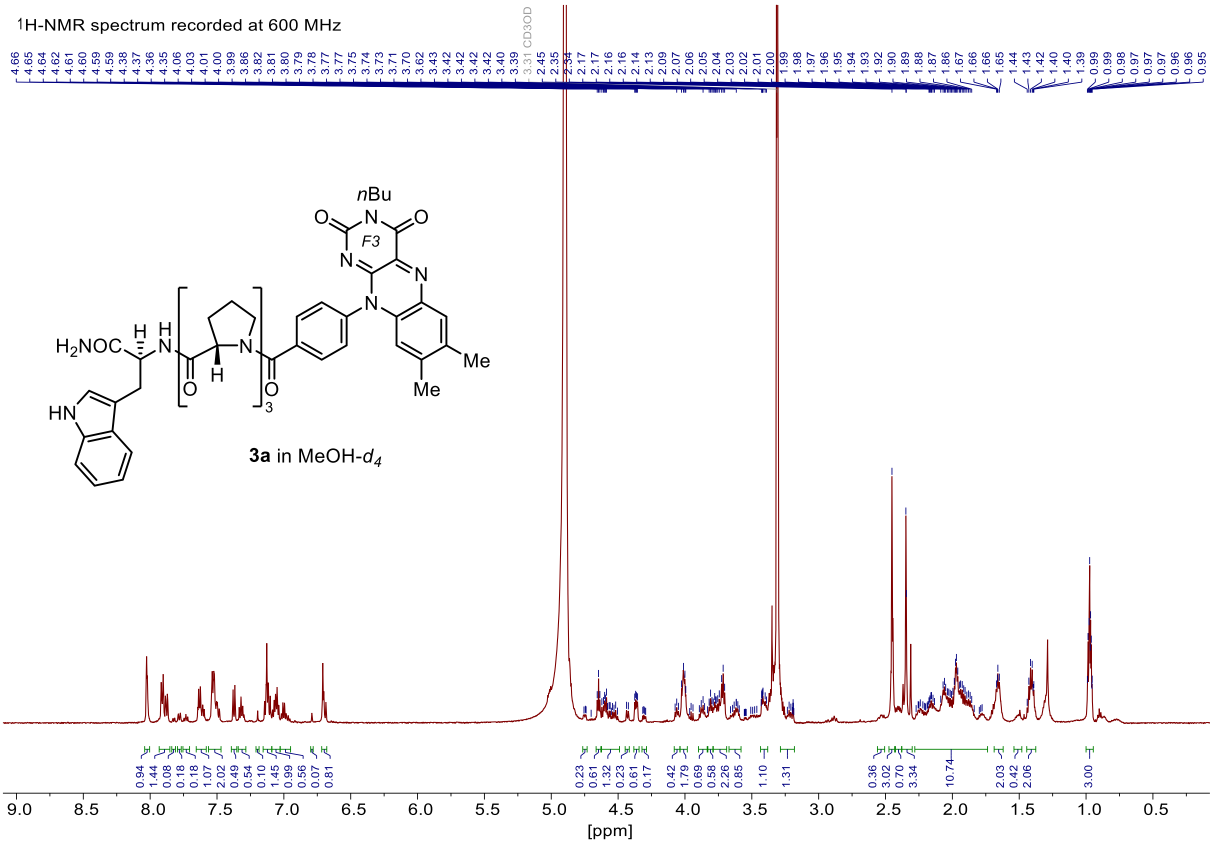
1H-NMR spectrum recorded at 600 MHz
nBu
F3
3a in MeOH-d4
[ppm]

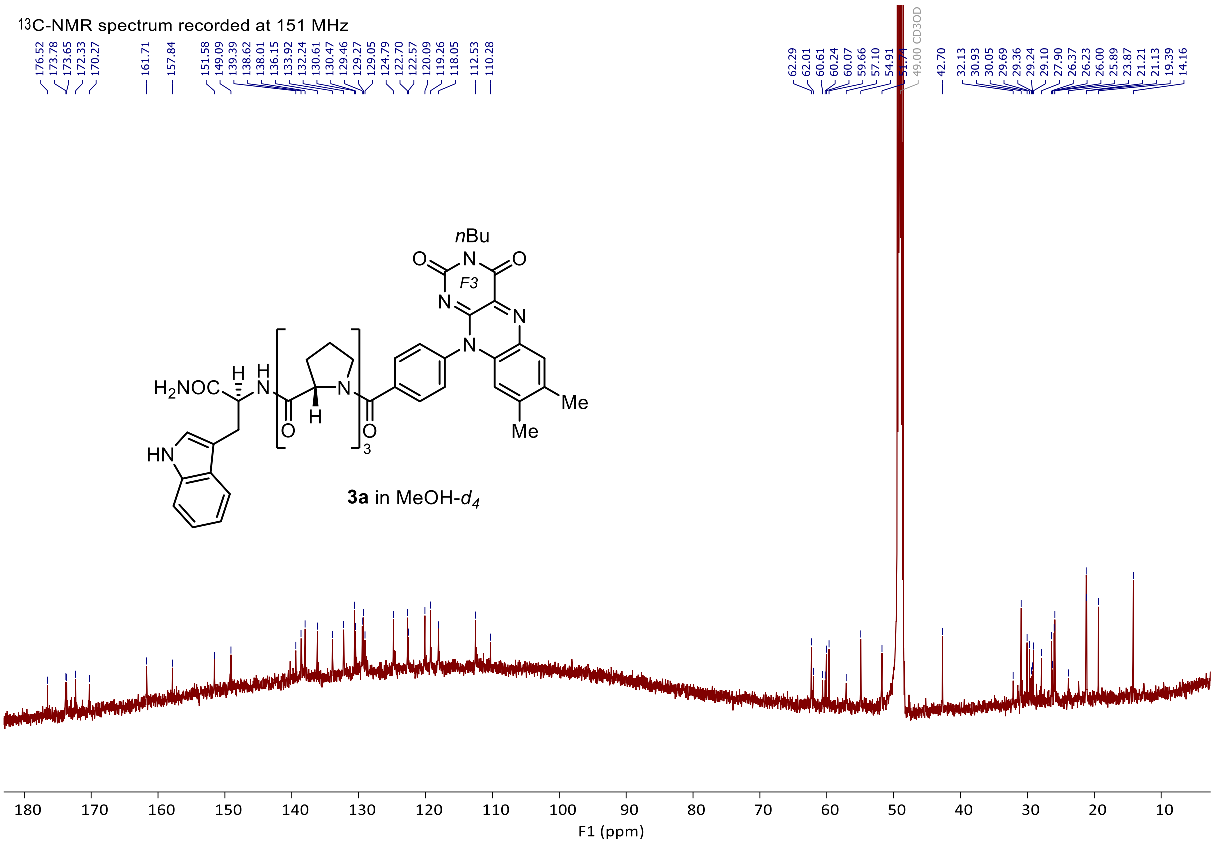
13C-NMR spectrum recorded at 151 MHz
nBu
F3
3a in MeOH-d4
F1 (ppm)

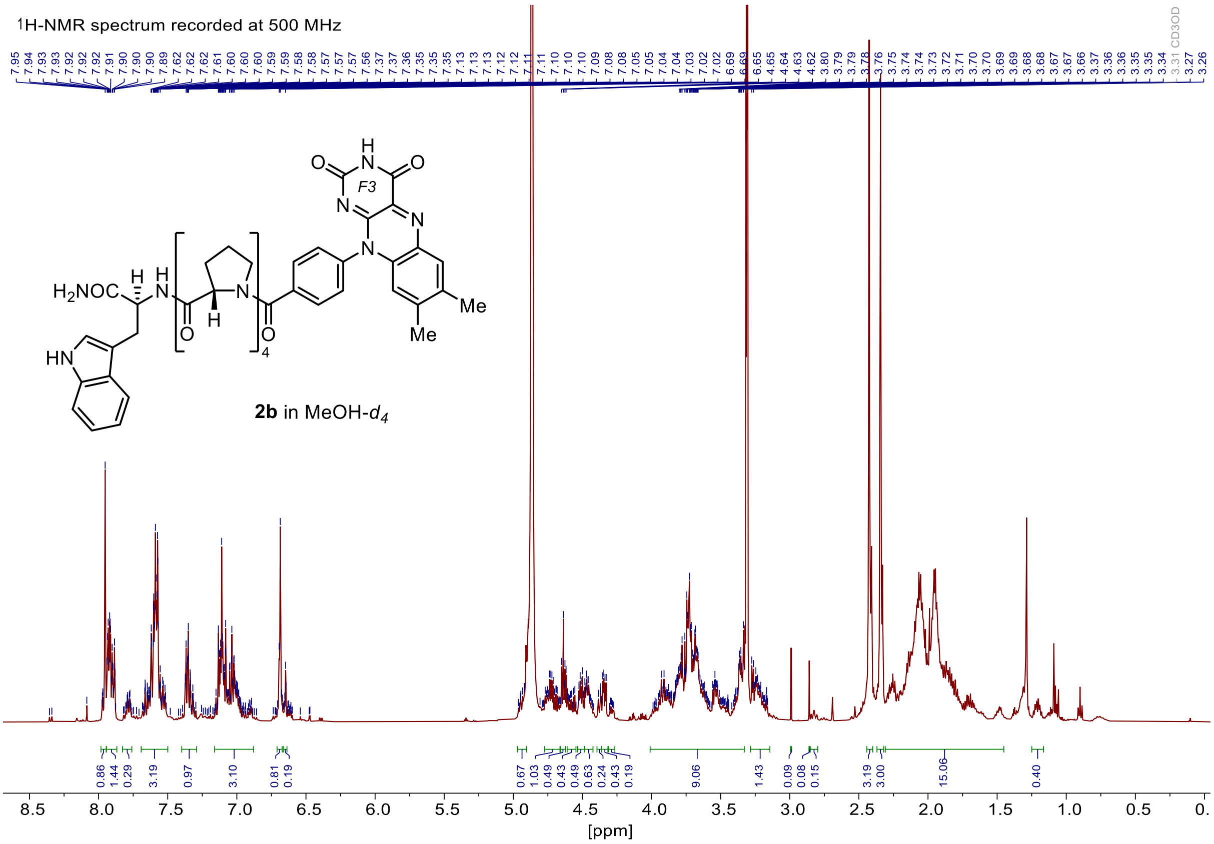
1H-NMR spectrum recorded at 500 MHz
2b in MeOH-d4
[ppm]

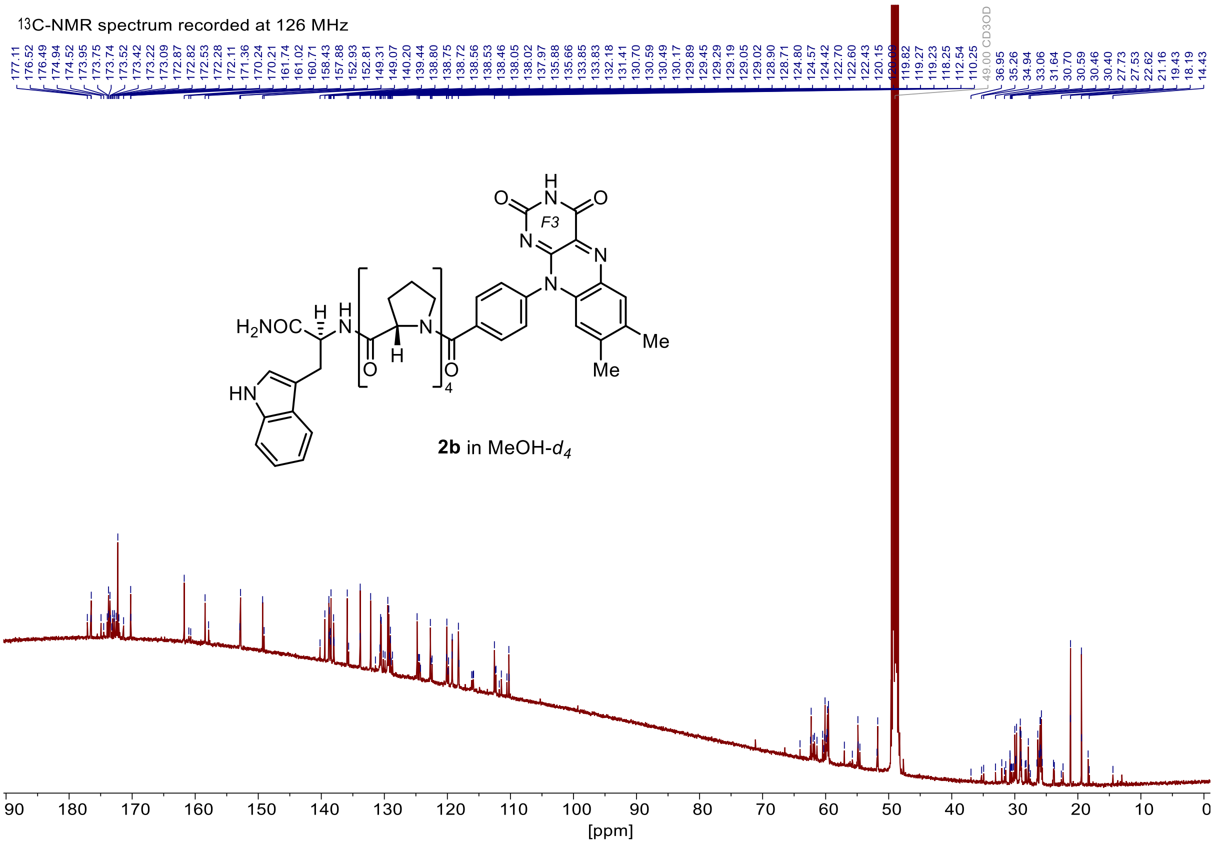
13C-NMR spectrum recorded at 126 MHz
2b in MeOH-d4
[ppm]

^{1}H-NMR spectrum recorded at 600 MHz

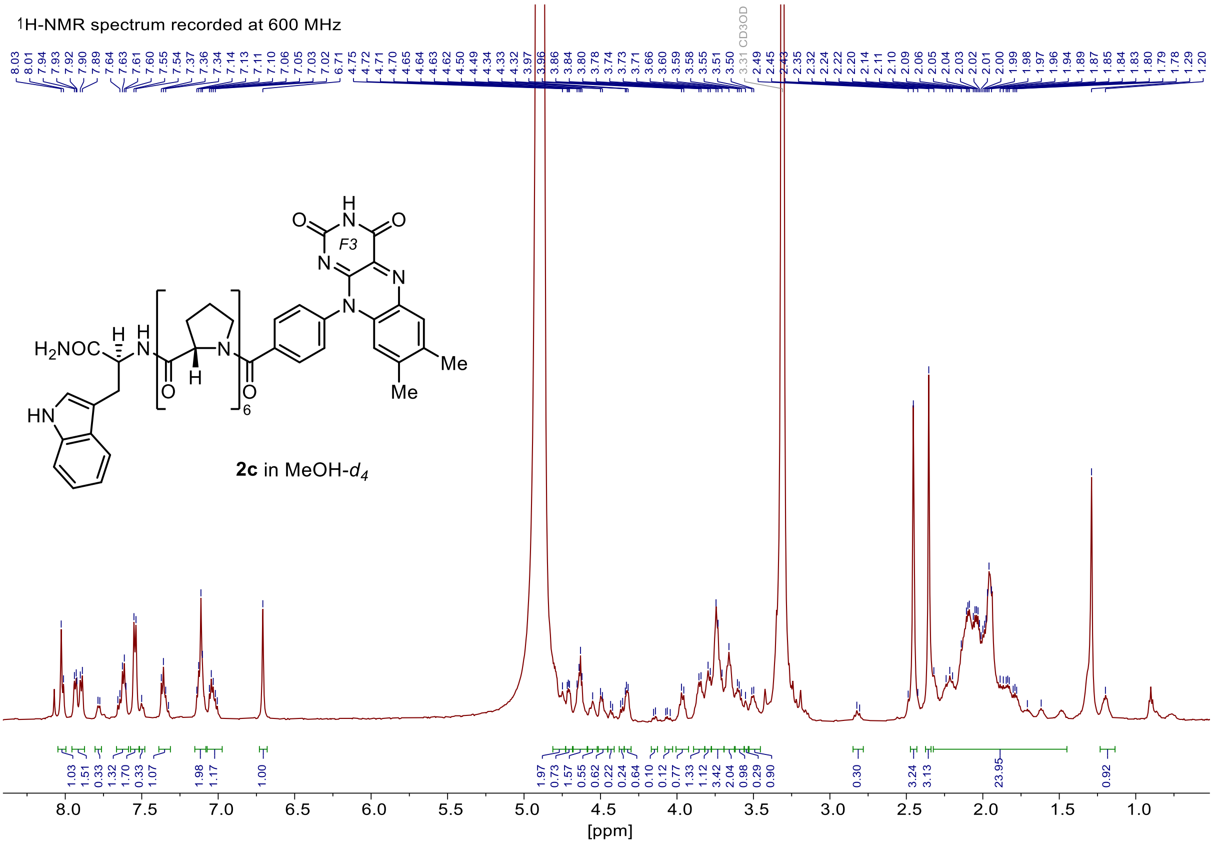

^{13}C-NMR spectrum recorded at 151 MHz

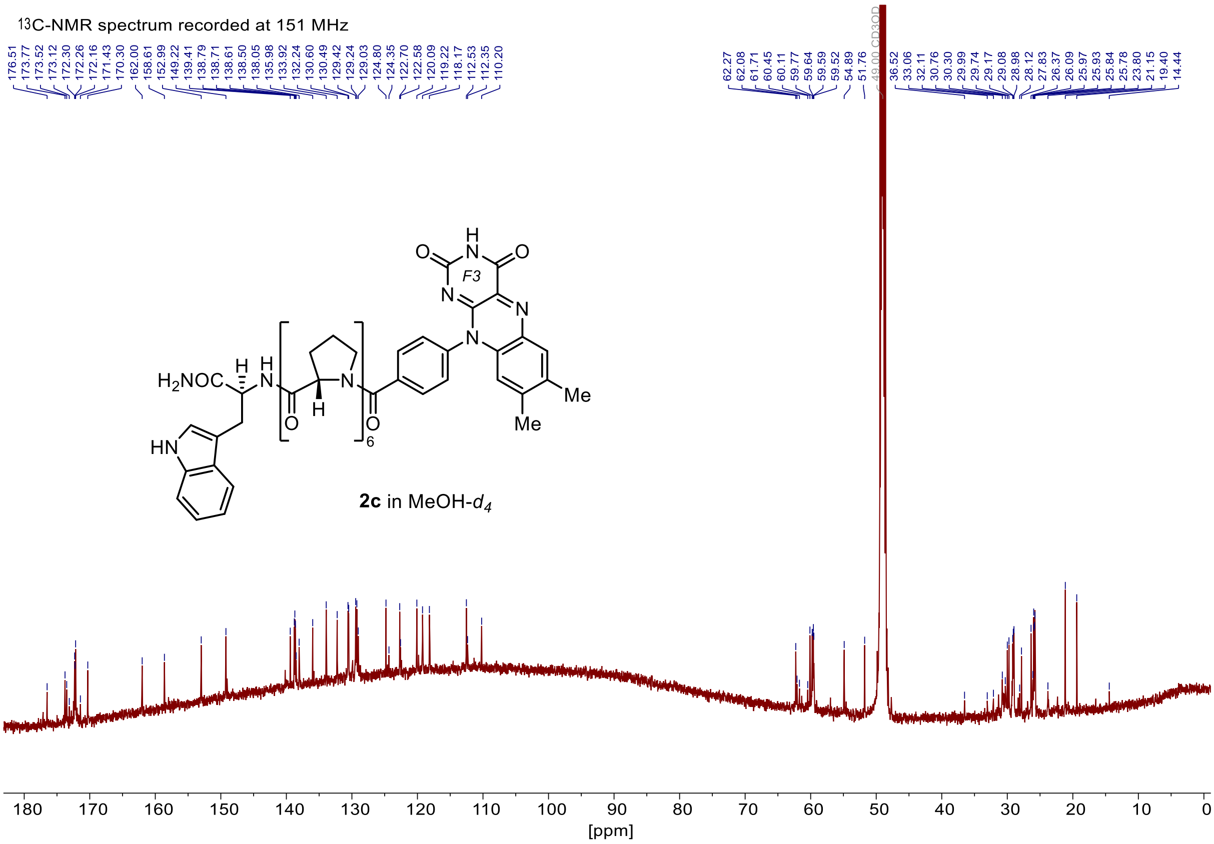

^{1}H-NMR spectrum recorded at 600 MHz

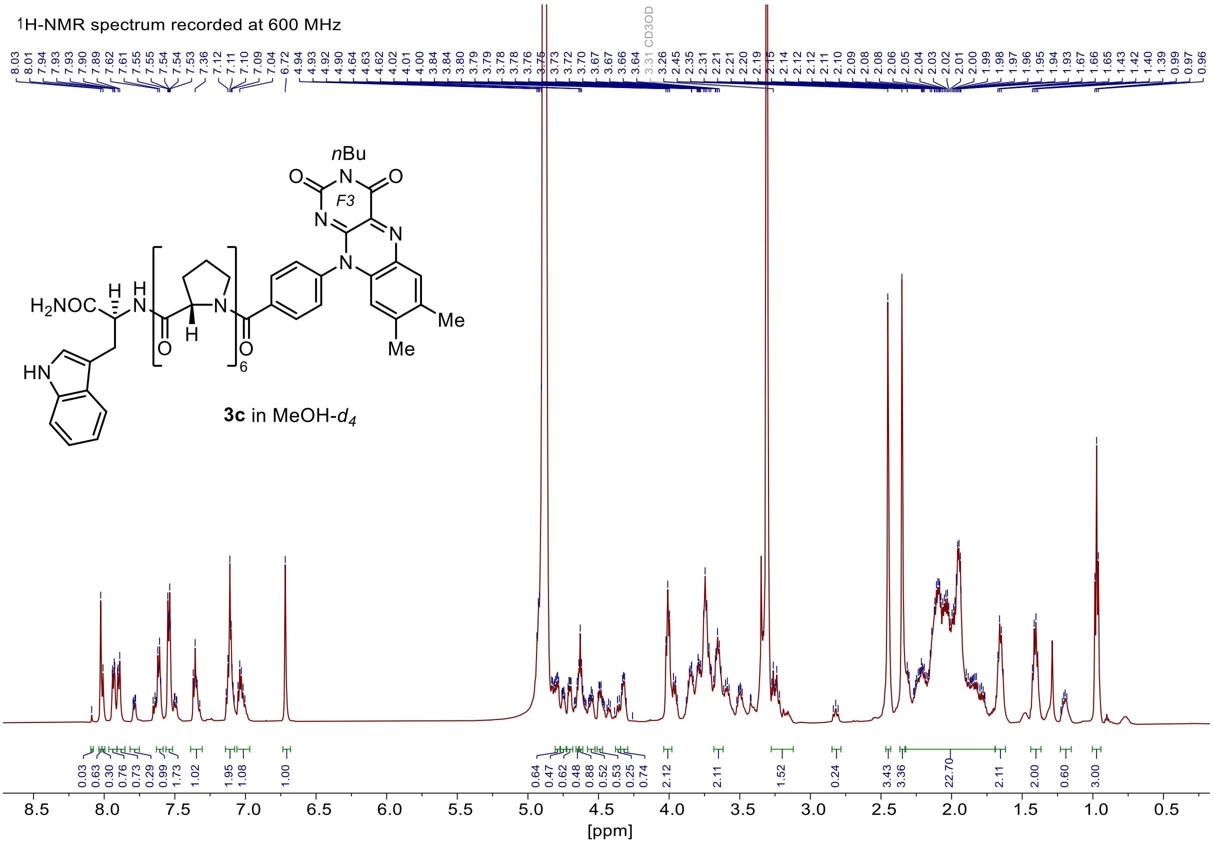

^{13}C-NMR spectrum recorded at 151 MHz

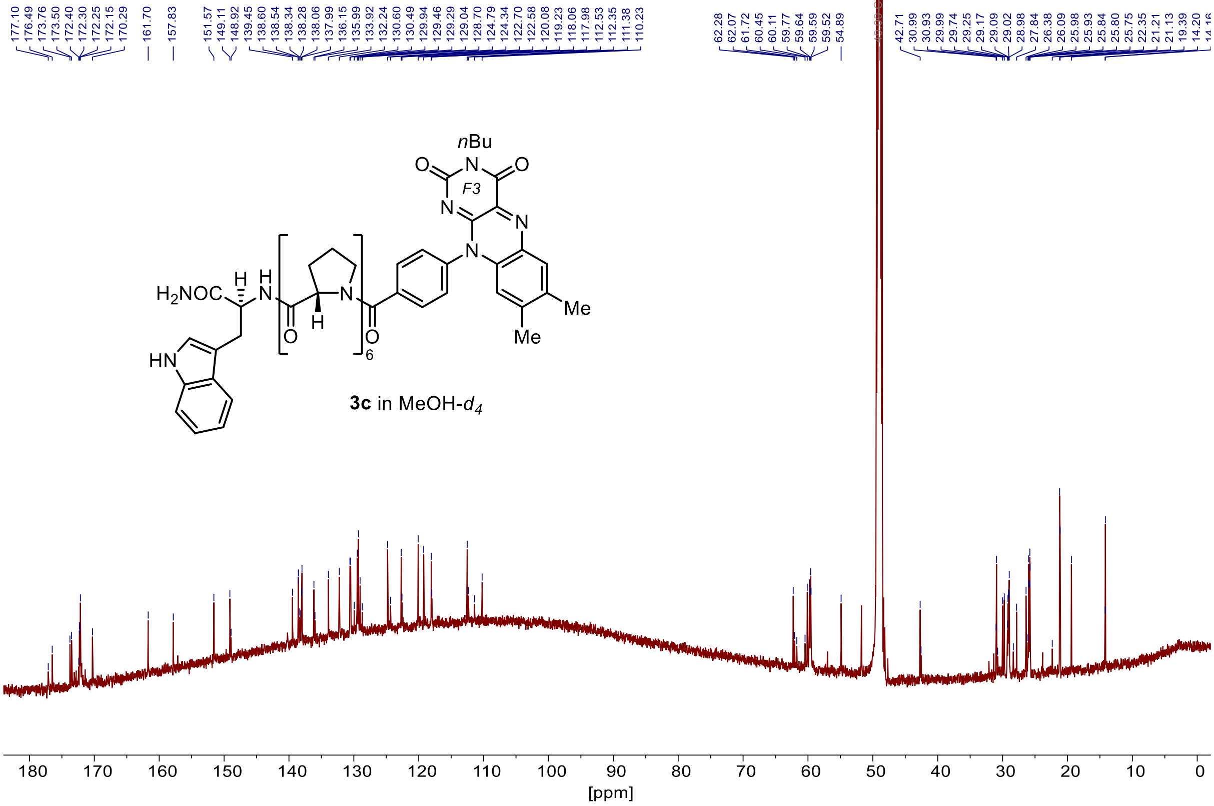

1H-NMR spectrum recorded at 600 MHz

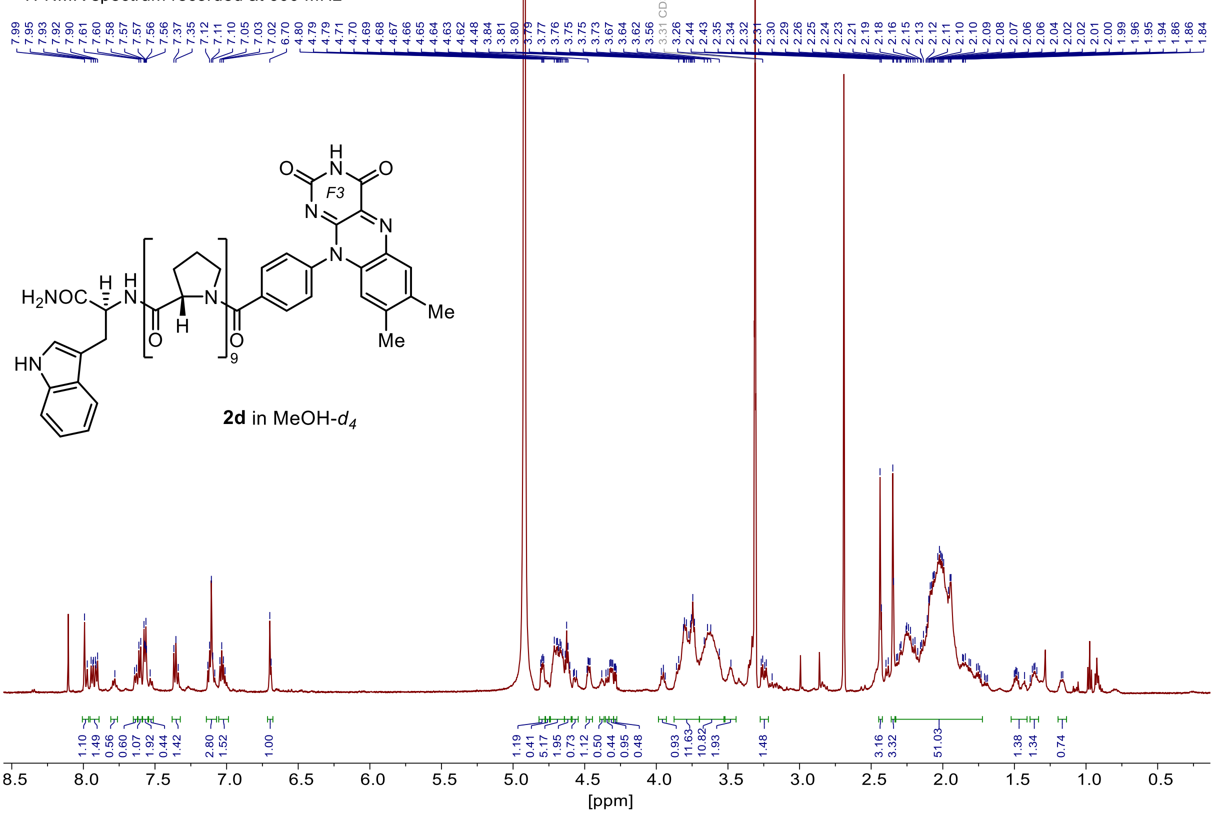

13C-NMR spectrum recorded at 151 MHz

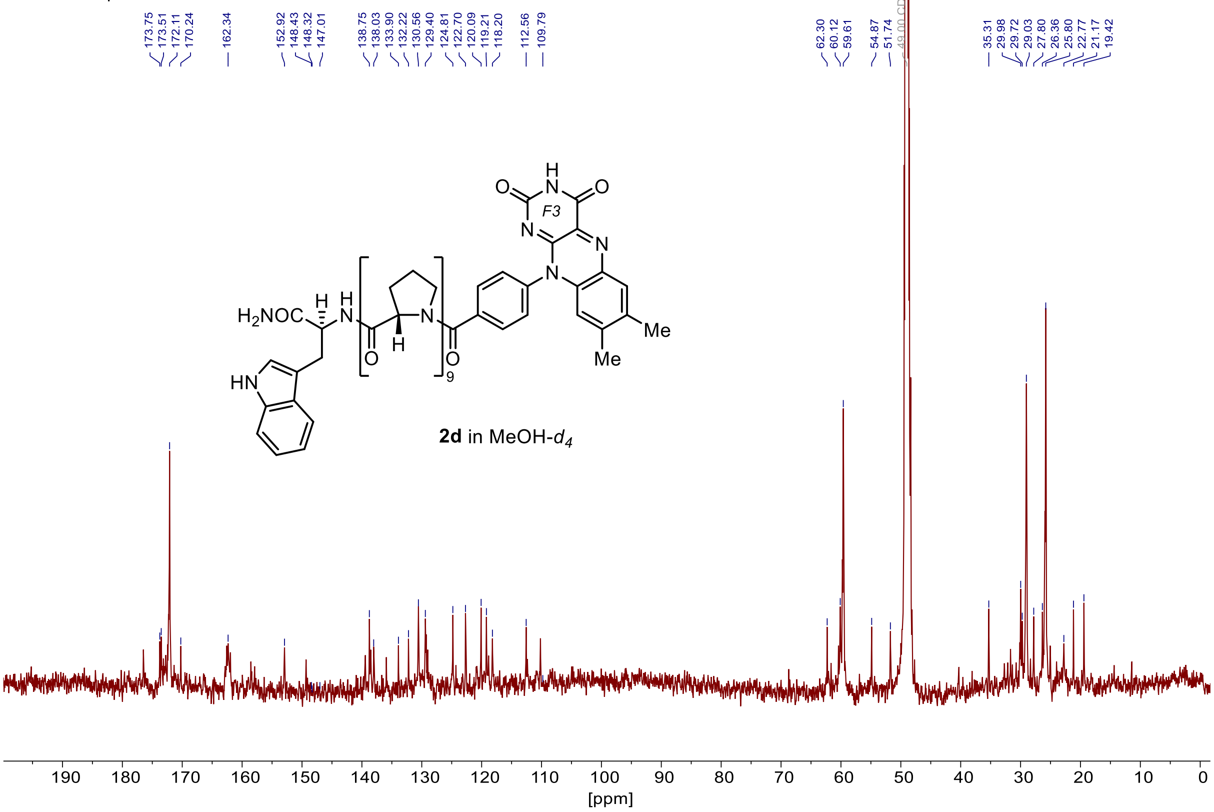

1H-NMR spectrum recorded at 600 MHz.

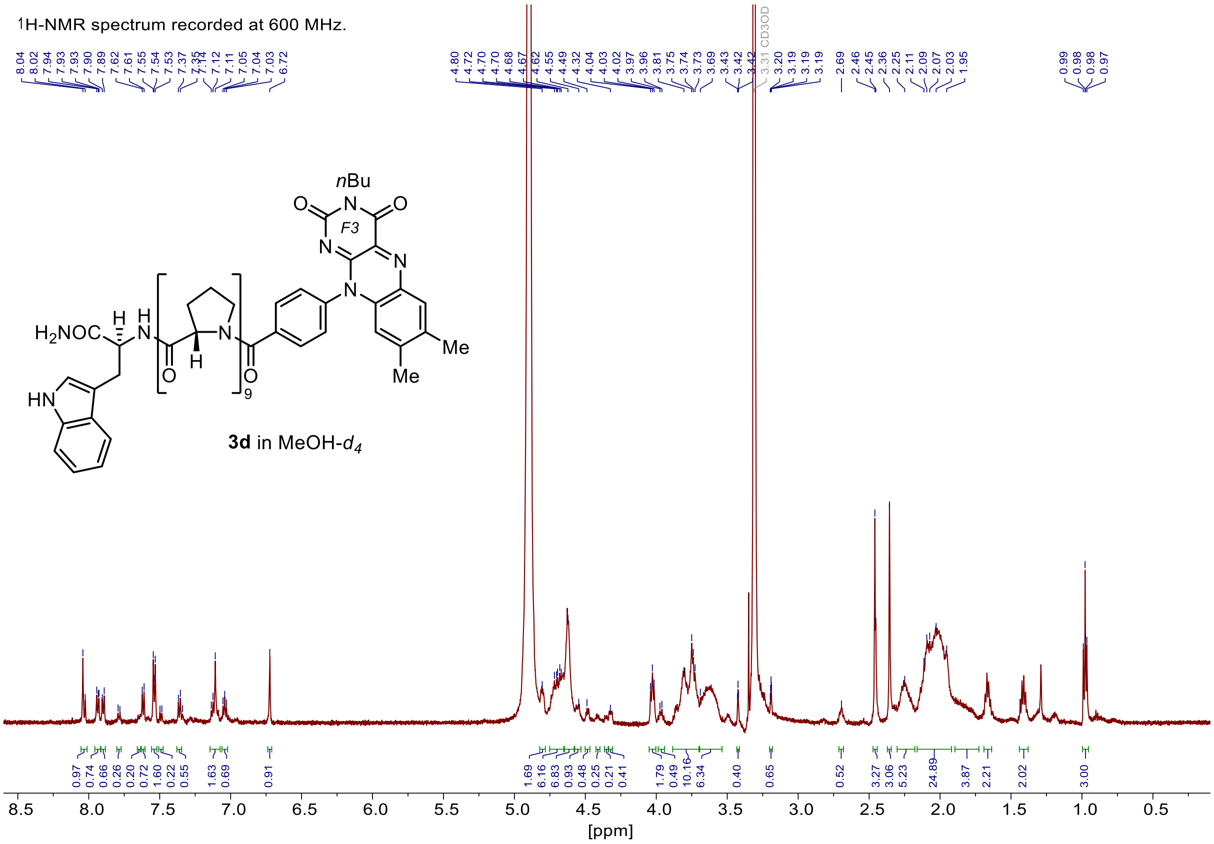

13C-NMR spectrum recorded at 126 MHz

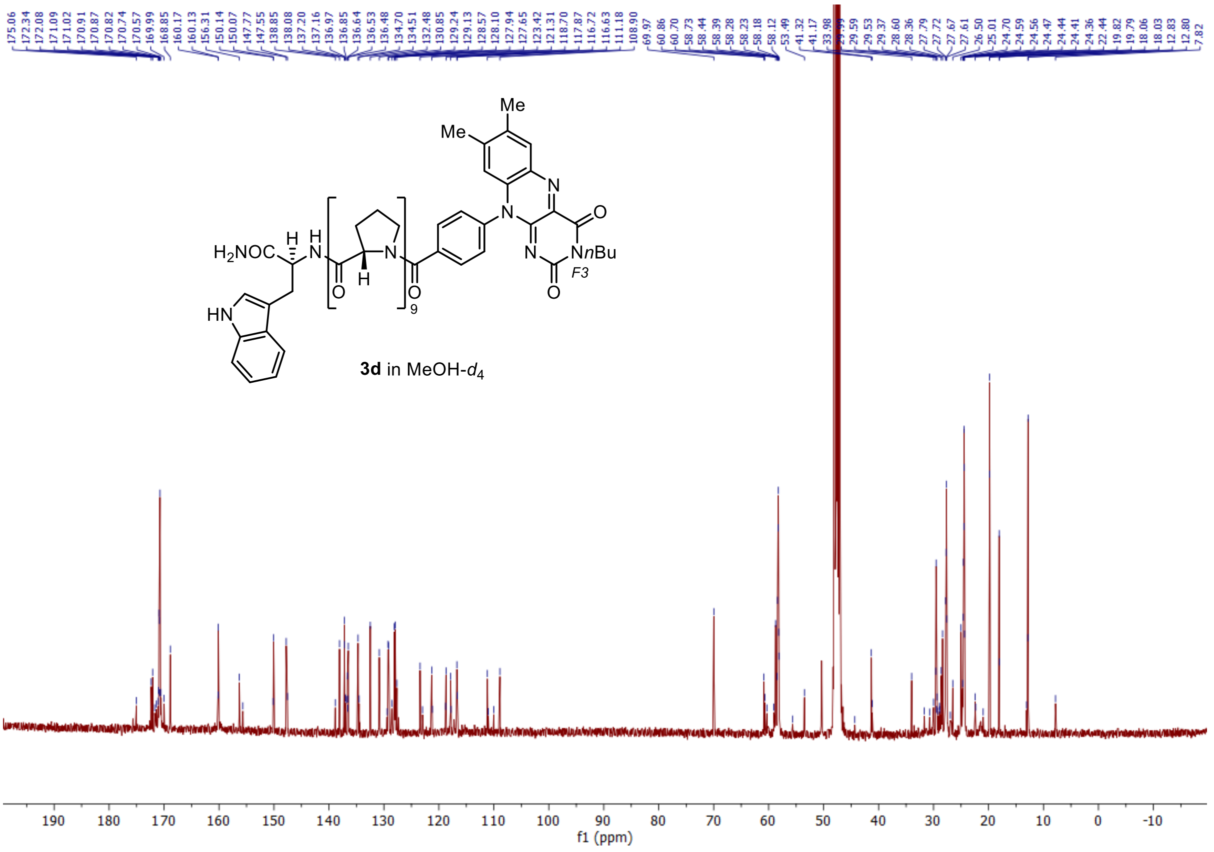

^{1}H-NMR spectrum recorded at 600 MHz

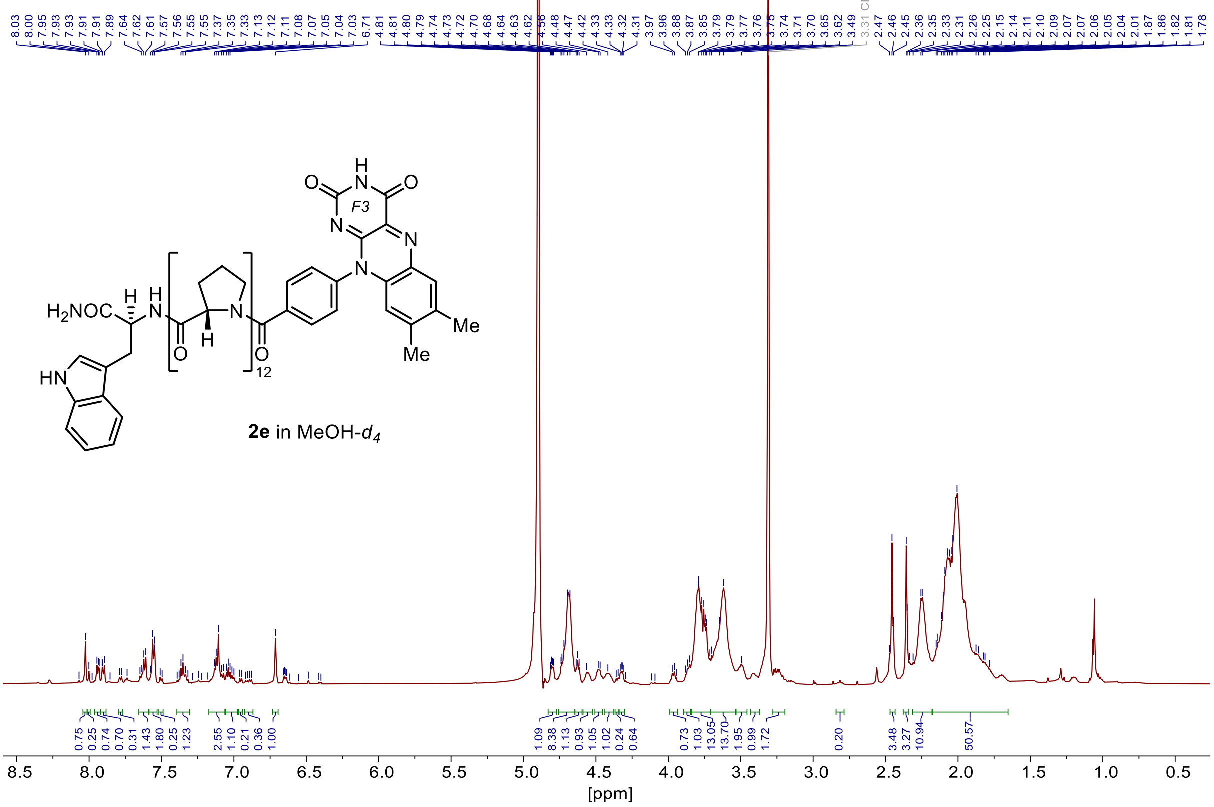

^{13}C-NMR spectrum recorded at 126 MHz

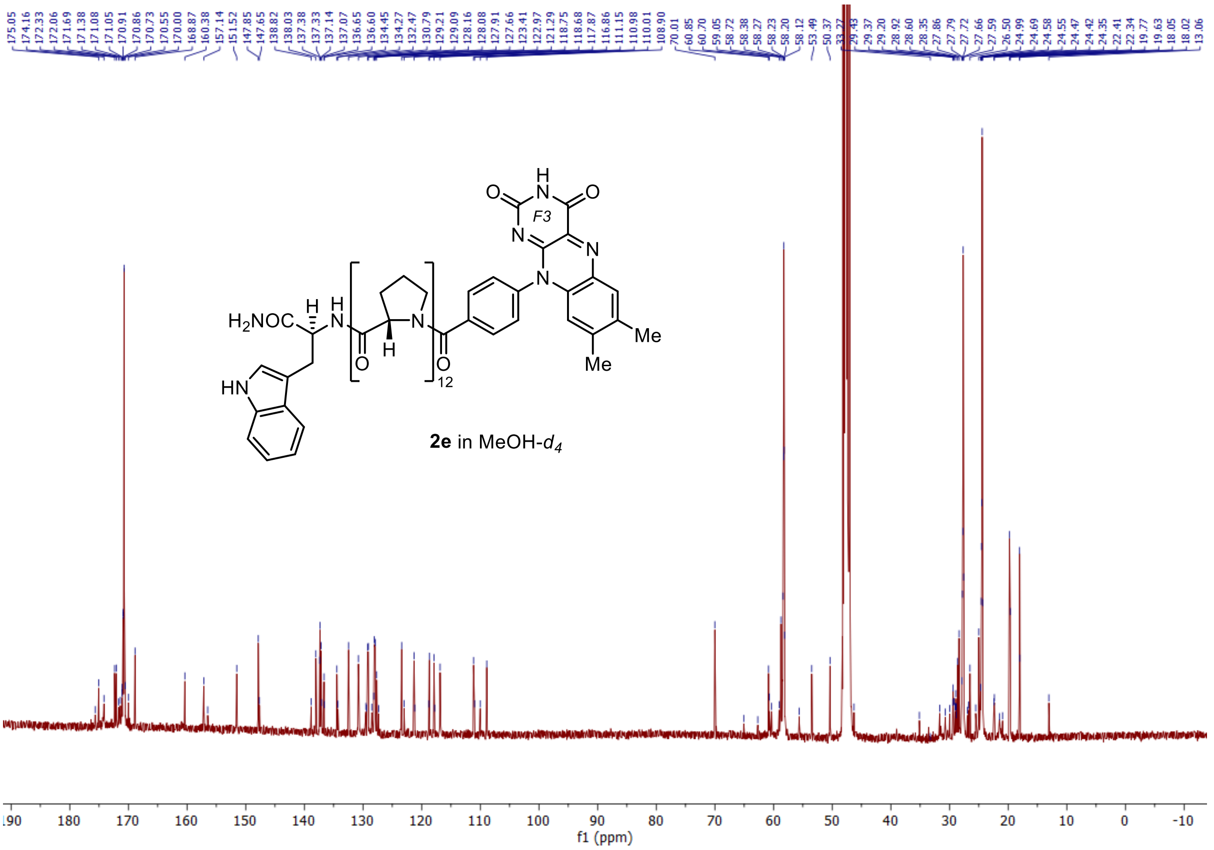

6. Experimental methods for ^{1}H-photo-CIDNP measurements

6.1 Materials and Methods

6.1.1 Sample Preparation

Diads **2** and **3** were dissolved in MeOH-d_4 (99.98% purity), which was purchased from Carl Roth GmbH+Co. KG, Karlsruhe, Germany, for UV-VIS and NMR spectroscopy. The concentrations used are specified with each experiment.

6.1.2 NMR Spectroscopy

For the NMR experiments, a Bruker-Spectrospin 600 UltraShield NMR spectrometer (Bruker Corporation, Karlsruhe, Germany) was used with a 5 mm TBI probe (PH TBI600S3 H/C-BB-D-05 Z LTB). Experiments were performed at 293 K. For the acquisition of thermally polarized ^{1}H spectra, a standard pulse sequence was employed. The samples were illuminated by the insertion of an optical fiber (diameter: 1mm, Thorlabs, Dachau, Germany) into the NMR tube via a coaxial insert (Wilmad WGS 5BL). For light illumination, a laser (MDL-III-445-1000mW) at 455 nm wavelength was used. A 600 mW output power was applied.

^{1}H-NMR data was collected with 32k complex data points. Assignments of ^{1}H- and ^{13}C-NMR signals of the artificial diads **2** and **3** were achieved from signal multiplicities, integral values, and characteristic chemical shifts from the through-bond correlations in 2D-COSY spectra, through-space correlations in 2D-NOESY spectra as well as from ^{1}H-^{13}C heteronuclear correlations in 2D-HSQC and HMBC spectra. The NMR data was analyzed using Bruker's TopSpin 4.0.9 and MestReNova 14.2.1 software.

6.1.3 ^{1}H-Photo-CIDNP Measurements

^{1}H-photo-CIDNP-NMR experiments were conducted using a presaturation pulse train combined with an additional destructive phase cycle, where every second scan included a light excitation pulse. The illumination time for all experiments was 0.5 s, the delay after illumination was 0.05 s, and the relaxation time was 0.5 s. The gradient pulse was 5 ms, and the gradient recovery delay was 0.2 ms. This method was employed to reduce errors stemming from the subtraction of light and dark spectra acquired in separate experiments. 32 scans were collected for each spectrum.

6.2 ¹H-Photo-CIDNP Measurement Setup and Spectra of Diads **2** and **3**

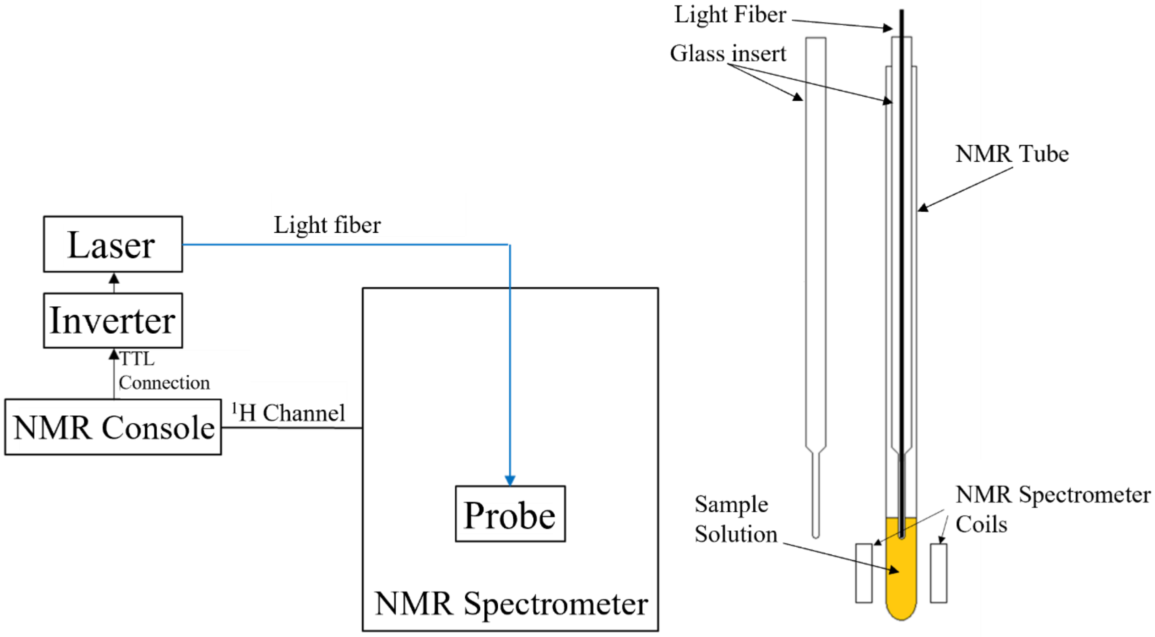

Figure S31: Experimental setup for photo-CIDNP-NMR measurements.

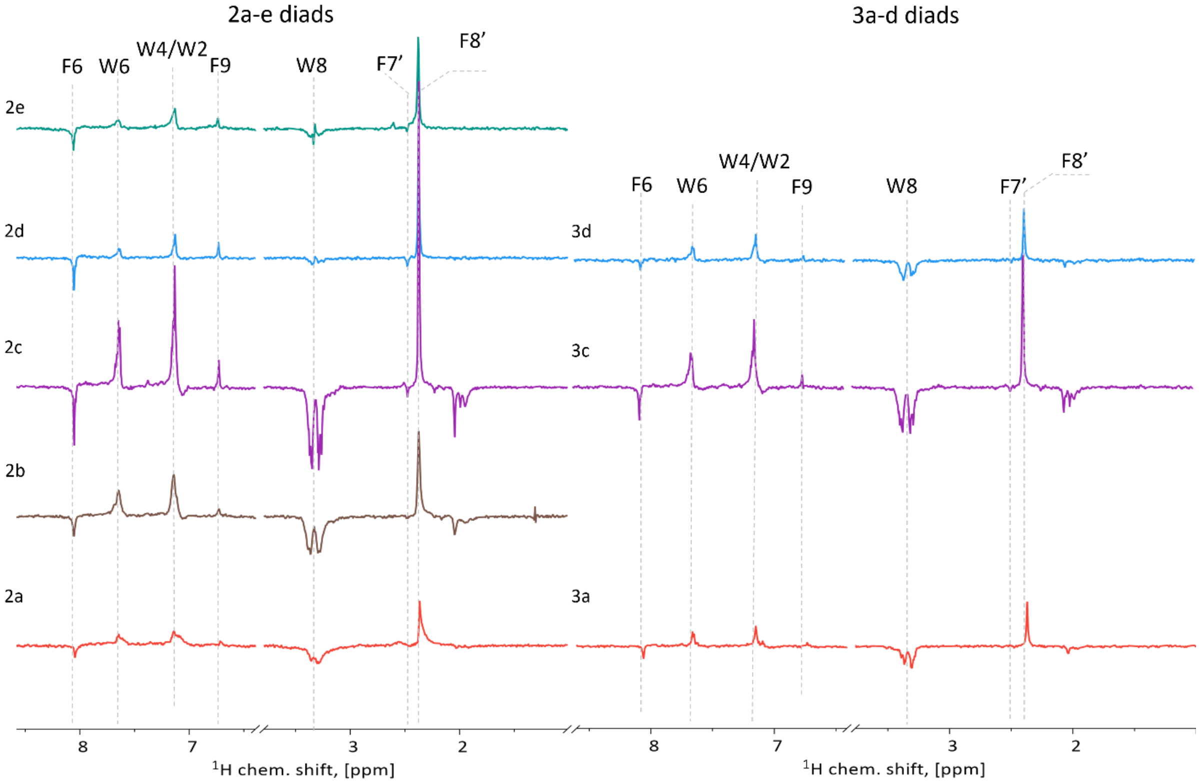

Figure S32: ¹H-photo-CIDNP spectra of diads **2a-2e** (0.2 mM concentration) and **3a**, **3b,** and **3d** (0.1 mM concentration) in MeOD-d_4, T=293 K; irradiated with 445 nm at 600 mW for 0.5 s.

Since our pulse sequence directly measures the difference signal (I_{light}- I_{dark}), we calculated the enhancement factor (EF) using the following ratio:

$$EF = \frac{I_{light}}{I_{dark}} = 1 + \Delta I / I_{dark} \qquad (1)$$

where ΔI is the signal observed in the difference spectrum and I_{dark} is the thermal (dark) signal. We computed EF values for three representative protons (*F6*, *W6*, and *F8'*) across eight different diads (see Table S3).

Table S3: The enhancement factors (ε) for the CIDNP effect for all studied diads.

Diad	Species	EF for F6	EF for W6	EF for F8'
**2**	**a**	0.4	1.6	1.9
	**b**	-0.1	4.7	1.7
	**c**	-0.5	4.3	2.8
	**d**	0.0	1.6	1.9
	**e**	-0.7	2.1	2.7
**3**	**a**	0.3	2.4	1.7
	**c**	-0.6	7.3	2.9
	**d**	0.5	2.0	1.5

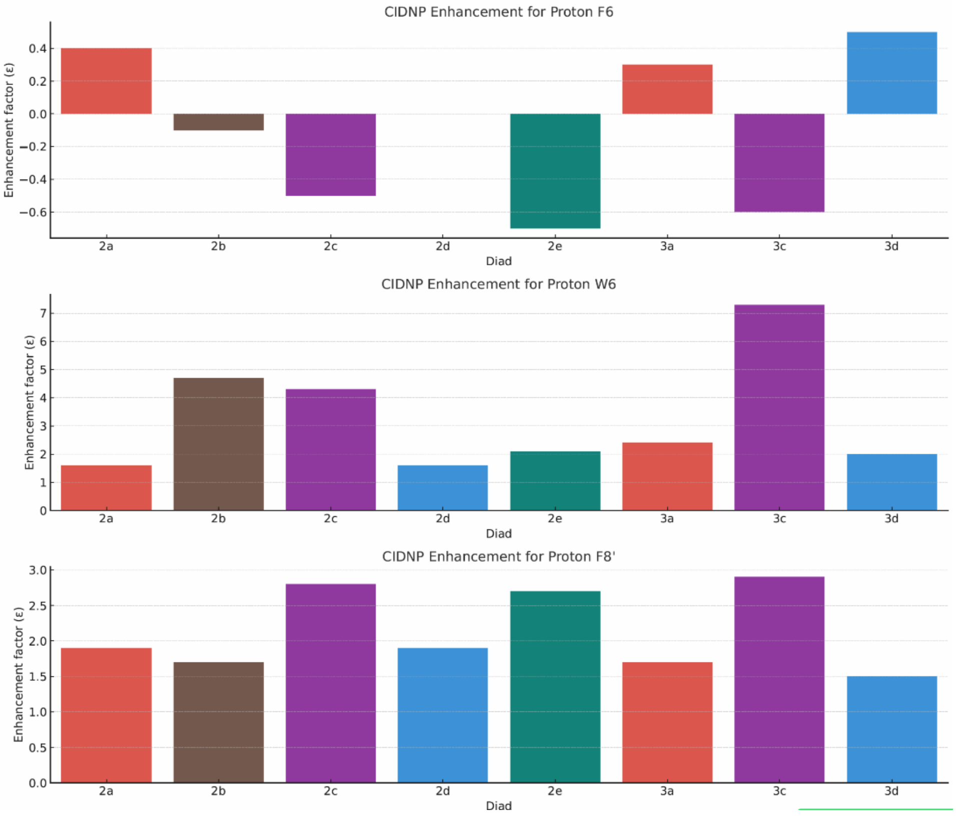

Figure S33: Photo-CIDNP enhancement factors (ε) for three representative protons (*F6*, *W6*, and *F8'*) across a series of diads (**2a–2e** 0.2 mM and **3a**, **3c**, **3d** 0.1 mM). The enhancement factor was calculated as the signal intensity ratio under irradiation relative to the thermal signal (see eq 1).

Proton F6 consistently exhibits negative enhancement. In contrast, protons *W6* and *F8′* show positive enhancements in all diads. Importantly, in the *N-n*Bu-substituted series, a clear structure-dependent trend is observed: the Pro-6 *N-n*Bu dyad shows the strongest overall enhancement, particularly at *W6* (EF = 7.34) and *F8′* (EF = 2.93), suggesting an optimal spatial arrangement for intramolecular spin polarization. This highlights a meaningful correlation between proline chain length and CIDNP efficiency in the *N-n*Bu series.

In contrast, the *N-H*-substituted dyads do not exhibit a similarly consistent trend. Although some *N-H* diads (e.g., 6pro *N-H*) also show significant enhancements, the overall pattern is more variable. This may reflect additional contributions from intermolecular interactions, such as hydrogen bonding or aggregation, which could interfere with or obscure the underlying structure-efficiency relationship.

6.3 Photochemical Stability of Diad **2c**

A

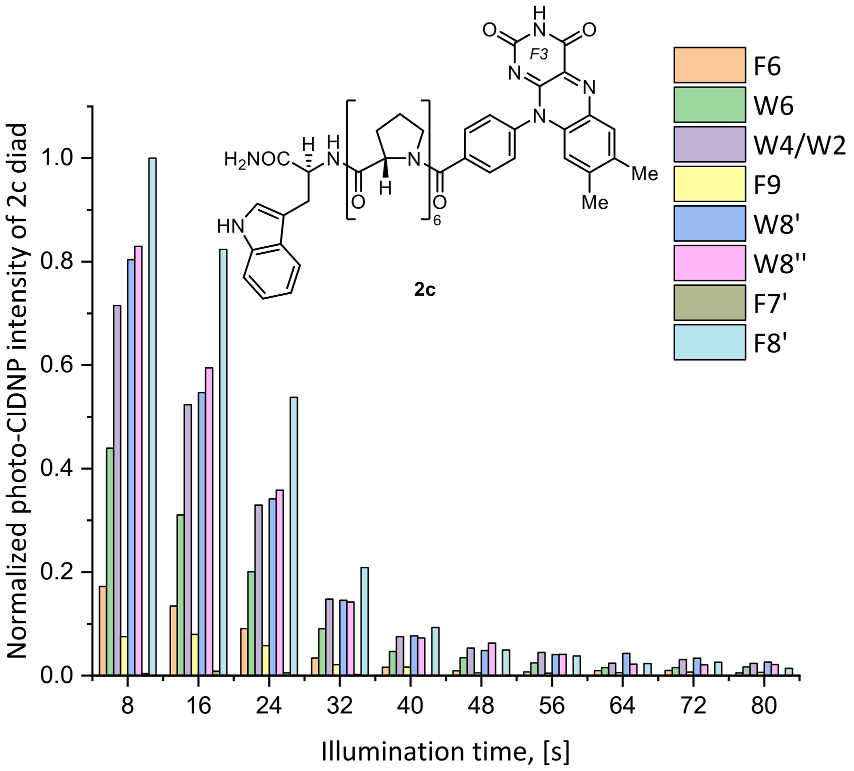

B

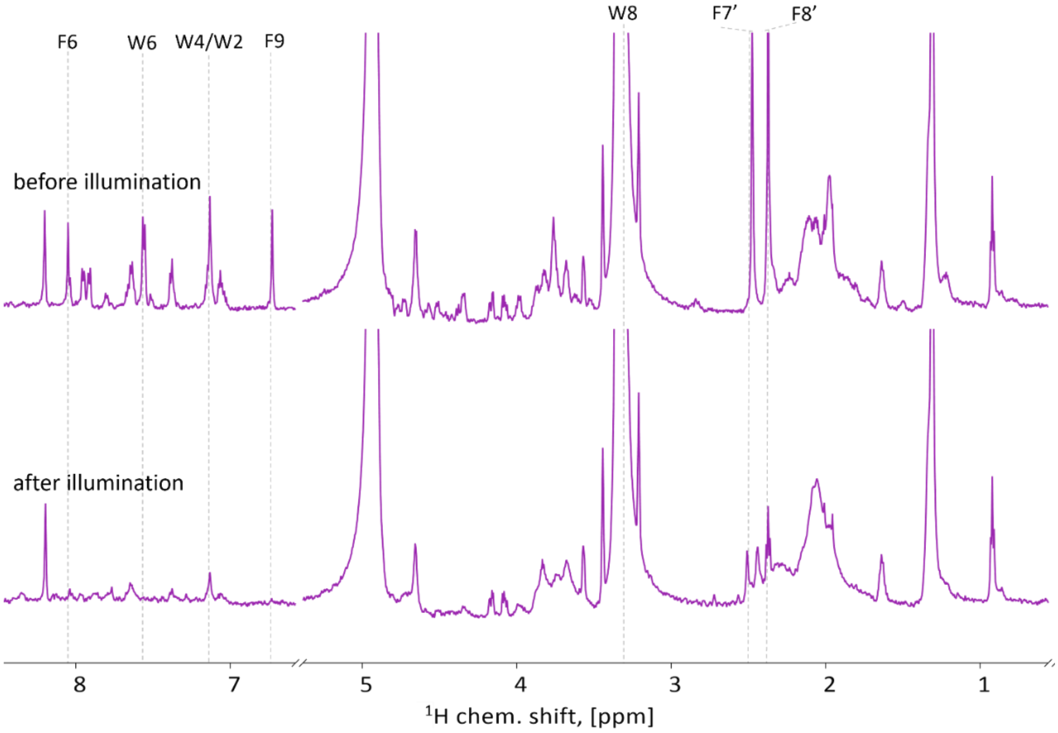

C

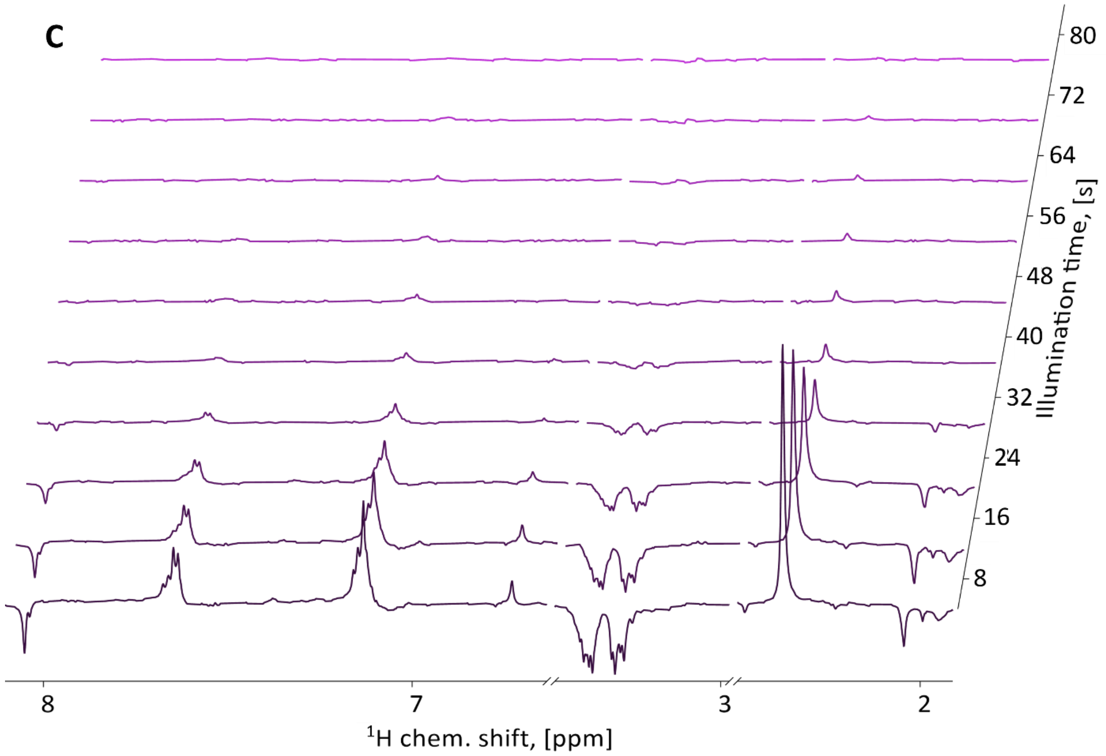

Figure S34: Decay of signal intensity for diad **2c**. **A)** Decay of the ^{1}H-photo-CIDNP effect for different protons over time; **B)** ^{1}H-NMR spectra before illumination and after 80 s of illumination; **C)** Time-dependent decay of ^{1}H-photo-CIDNP spectra. All measurements were performed in 200 mM in MeOD-d_4, T = 293 K; irradiated with 445 nm laser at 600 mW for 0.5 s.

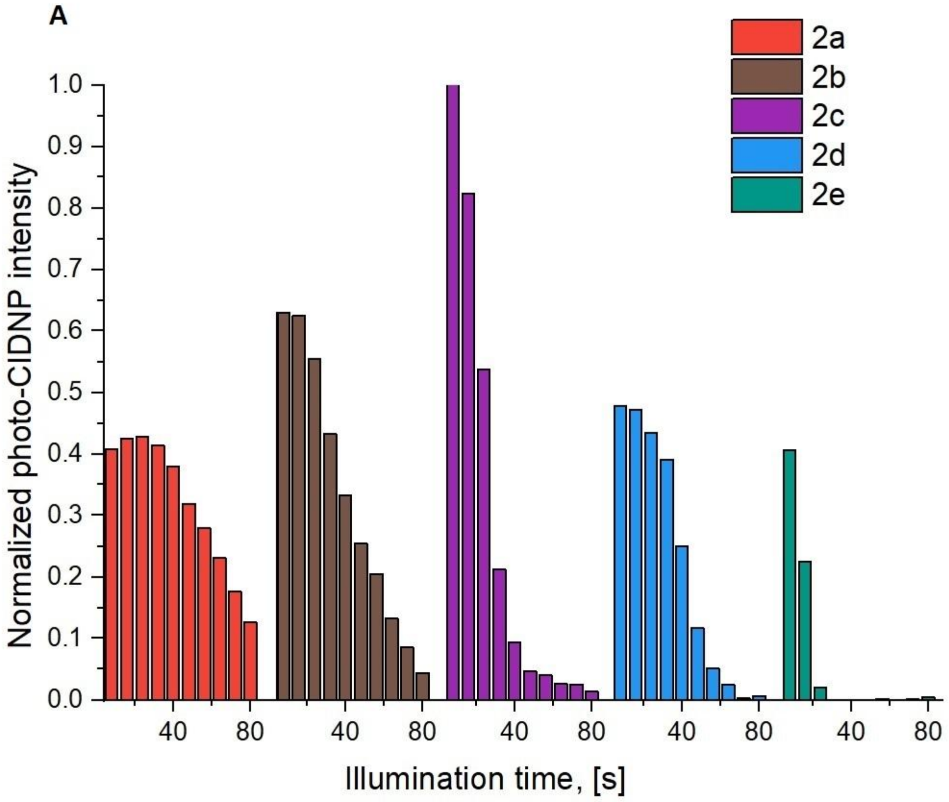

Figure S35: Time-dependent decay of ^{1}H-photo-CIDNP signal intensities of F8'-methyl protons in MeOD-d_4 in diads **2a**-**2e**, T=293 K; irradiated 445 nm at 600 mW for 0.5 s.

6.4 Temperature Dependent Experiment of Diads 2d and 3d

The intensity of photo-CIDNP signal was shown to be temperature dependent. In the case of diad **2d**, an enhanced photo-CIDNP intensity was observed with increasing temperature, suggesting that intermolecular interactions may become more favourable or effective at elevated temperatures.

For the diad**, 3d** temperature dependence shows a less straightforward pattern; however, a general increase in photo-CIDNP intensity with rising temperature is still observed. This suggests that similar intermolecular interactions may have a place, although the underlying mechanisms might be more complex in this case.

These findings support the hypothesis that intermolecular hydrogen bonding significantly influences the photoinduced spin dynamics in protic solvents such as methanol.

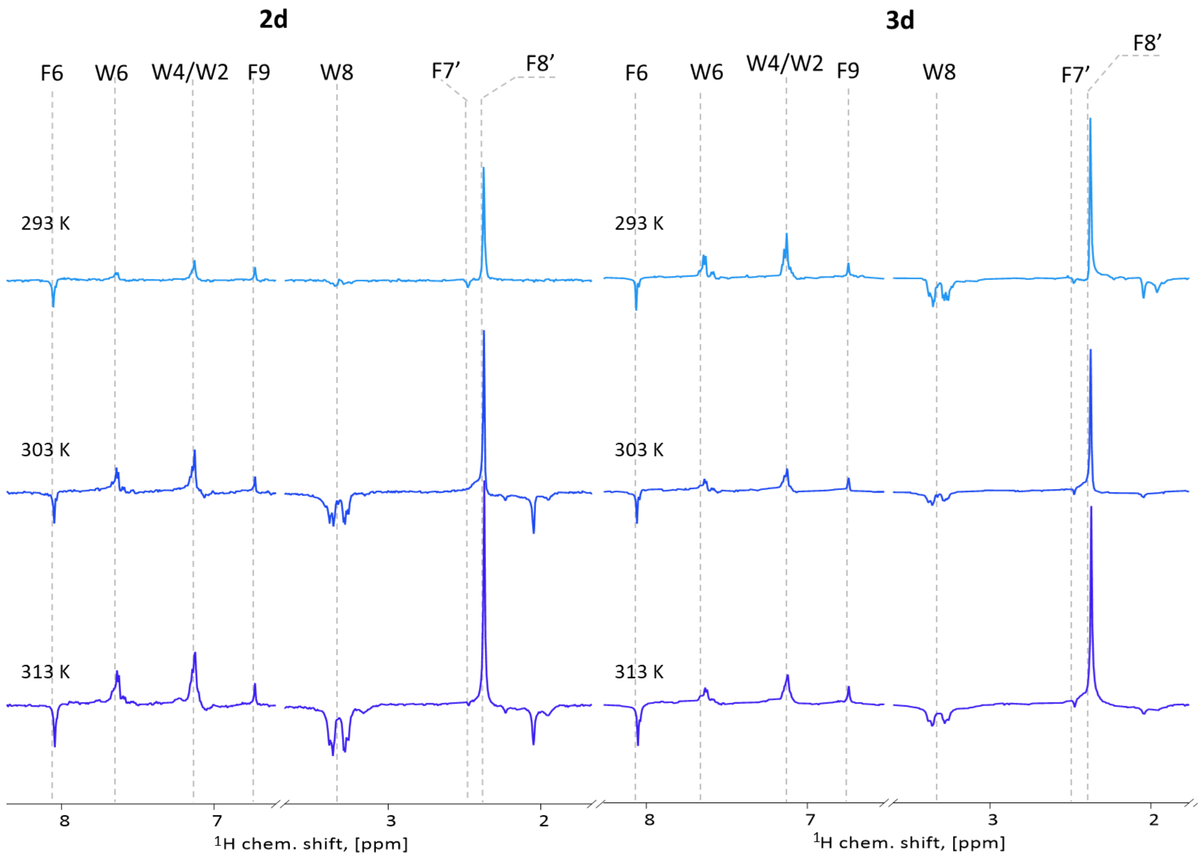

Figure S36: Temperature dependent ^{1}H-photo-CIDNP spectra of diads **2d** and **3d** (0.2 mM concentration) in MeOD-d_4; irradiated with 445 nm at 600 mW for 0.5 s.

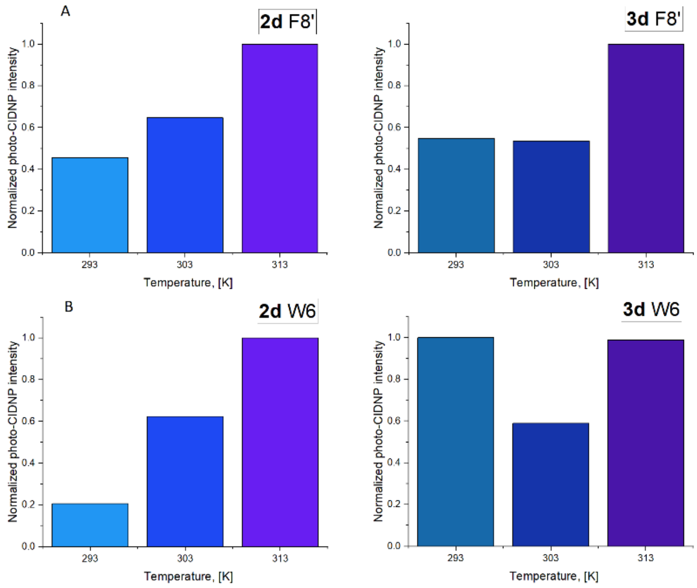

Figure S37: Normalized ^{1}H-photo-CIDNP intensities for diads **2d** and **3d** at different temperatures. Panel A corresponds to *F8’* proton and Panel B to *W6* proton resonances. Spectra obtained at 0.2 mM concentration in MeOD-d_4; irradiated with 445 nm at 600 mW for 0.5 s. Signals of the corresponding peaks were integrated and normalized with the most intense one set to 1.

7. Molecular dynamics simulations

Molecular dynamics (MD) simulations were performed using NAMD[5-6] utilizing the CHARMM36 force field.[7-13] Parameters for the flavin and tryptophan radicals were adapted from earlier studies.[14-15] All MD simulations assumed the diads **2** and **3** to be solvated in MeOH-d_4, modeled using the parameters available for regular methanol,[12-13] but with the changed mass of the deuterium atoms. The complete list of parameters used in the simulations is included in the Supporting File 1.

The atomistic structures of the *N-H* **2** and *N-n*Bu **3** diads were constructed for the five different lengths of the proline linker employing the GaussView program.[16] Six different diad conformations were obtained for each structure using the software package CREST;[17-18] these structures were used as initial diad configurations in the MD simulations. The six structures were obtained from meta dynamics that explored the conformational space at a temperature of T = 400 K. Conformers were selected within an energy threshold of Ew = 10.0 kcal/mol calculated from the energy of the most stable conformer. Six of the most stable but distinctly different conformers were selected. Exemplary PSF and PDB files for the **3a** diad used for the simulations are included as Supporting files 2 and 3, respectively.

All MD simulations included diads placed in a rectangular simulation box of MeOH-d_4. All MD simulations included the equilibration phase of 21 ns, followed by the production simulation phase of 240 ns (carried out for each diad and six replica simulations). Equilibration stages were performed under pressure control, employing the using the Nosé -Hoover Langevin piston algorithm[19-20] with a target pressure of 1.01325 bar, a period time of 200 fs and a decay time of 50 fs. Long-range electrostatics were calculated directly with a cutoff distance of 12 Å. Periodic boundary conditions were imposed. The temperature was controlled at 293 K using the Langevin thermostat.[6]

Following the equilibration simulations, each diad was further simulated for 240 ns, assuming the canonical NVT ensemble, resulting in a total simulation time of 1.44 µs, being long enough to extensively sample the conformational space of the studied molecules. The simulation box sizes used for each diad after equilibration are compiled in Table S4. Several exemplary renderings of the diads are presented in supplementary videos 1-5.

The distances and relative angles between the Trp and Flavin moieties within the diads were analyzed using VMD.[21] The relative angle α between two normal vectors $\mathbf{n_1}$ and $\mathbf{n_2}$ (see Figure 2B) defined perpendicular to the planes of the Trp and Flavin cofactors was determined as

$$\cos\alpha = \frac{(\mathbf{n_1},\mathbf{n_2})}{|\mathbf{n_1}||\,\mathbf{n_2}|}\,. \qquad (2)$$

Figures **S38-S42** illustrate the temporal evolution of the center-to-center distances d and the relative angle α between the Trp and the Flavin molecules obtained from the MD simulations for *N-H* **2** and *N-nBu* **3** diad variants. The results sampled across all six replica simulations were used to obtain the corresponding probability densities for d and α, as shown in Figure 2 and Figure **S43**, respectively. The figures show the distributions for the *N-H* and *N-n*Bu diad systems with different linker lengths. The visualization of the representative diad structures with the most probable distance and angle is further illustrated in Figure **S44**.

Table S4. Box sizes used for simulation after equilibration for all diads and six different replicas.

Diad	Replica	10			11		
		x, Å	y, Å	z, Å	x, Å	y, Å	z, Å
a	1	82.39	82.13	81.71	83.25	83.16	82.97
	2	82.04	81.78	81.61	82.78	82.56	82.53
	3	82.33	82.19	81.79	82.39	82.21	81.74
	4	82.22	81.83	81.66	82.58	82.32	82.47
	5	82.36	82.20	81.80	82.66	82.65	82.00
	6	82.51	82.25	81.83	82.56	82.55	81.91
b	1	83.24	83.14	82.95	76.04	76.21	76.33
	2	82.68	82.46	82.43	76.56	76.30	76.30
	3	82.55	82.37	81.90	76.57	76.32	76.31
	4	82.70	82.44	82.59	77.03	77.16	76.94
	5	82.68	82.67	82.03	77.05	76.99	77.13
	6	82.57	82.56	81.92	76.97	77.52	77.35
c	1	75.88	76.04	76.17	86.46	85.68	85.28
	2	76.26	76.10	76.23	86.57	85.79	85.39
	3	75.97	76.14	76.27	86.53	85.75	85.35
	4	76.10	76.27	76.40	86.38	85.60	85.20
	5	76.42	76.26	76.39	86.50	85.71	85.32
	6	76.49	76.44	76.45	86.59	85.81	85.41
d	1	86.48	85.70	85.31	86.53	85.75	85.36

	2	86.51	85.72	85.33	86.54	85.75	85.36
	3	86.51	85.73	85.33	86.46	85.67	85.28
	4	86.41	85.63	85.23	86.43	85.65	85.25
	5	86.54	85.76	85.36	86.60	85.82	85.42
	6	86.40	85.62	85.23	86.56	85.78	85.38
**e**	1	86.55	85.77	85.37	86.53	85.75	85.36
	2	86.57	85.78	85.39	86.54	85.75	85.36
	3	86.54	85.76	85.36	86.46	85.67	85.28
	4	86.53	85.75	85.35	86.43	85.65	85.25
	5	86.64	85.86	85.46	86.60	85.82	85.42
	6	86.51	85.72	85.33	86.56	85.78	85.38

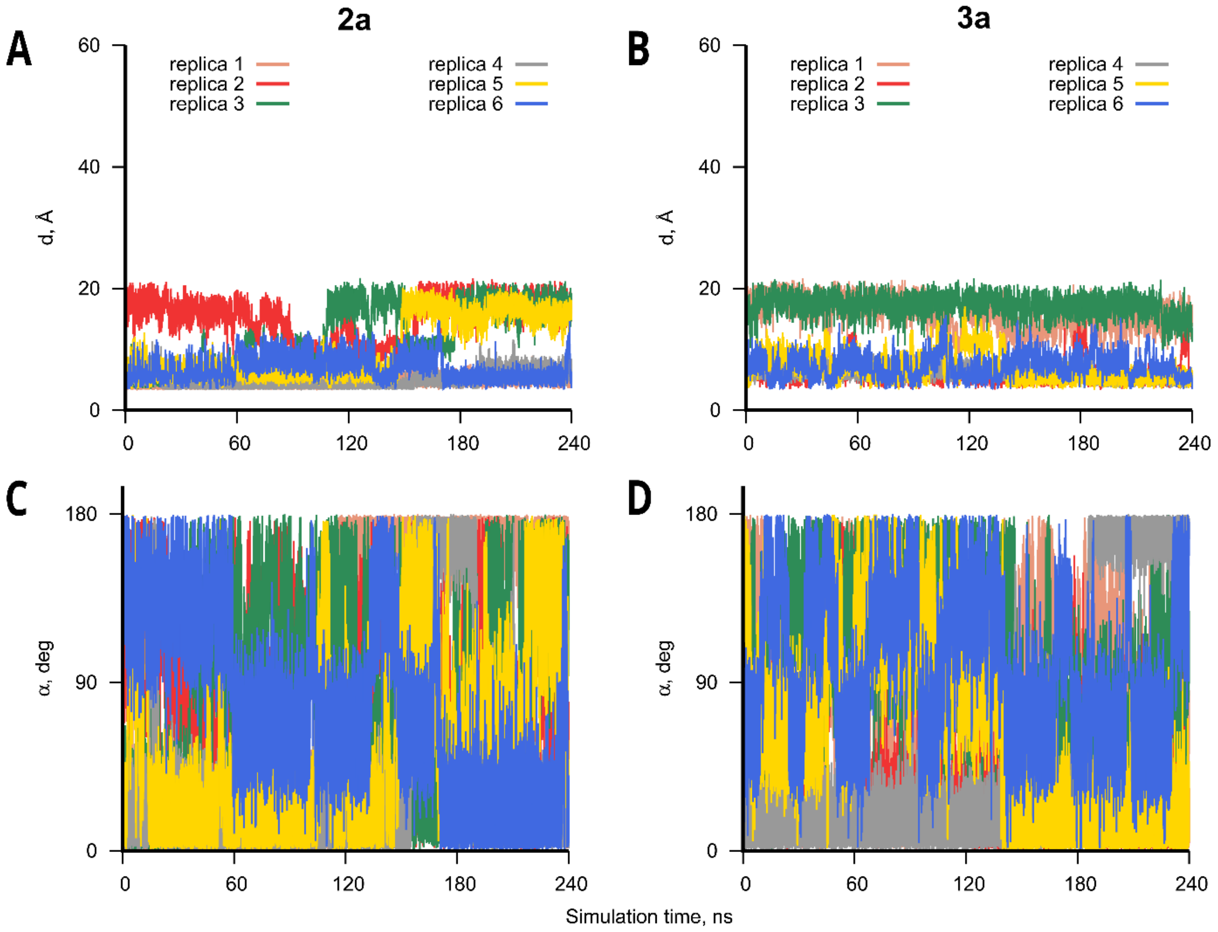

Figure S38: **A**) Temporal evolution of the distance **d** (see Figure 2A) obtained from MD simulations of Pro3-*N*-*H* diad **2a** for different simulation replicas. **B**) Similar to **A**), but computed for the Pro-3-*N*-*n*Bu diad **3a**. **C**) Time evolution of the relative angle α defined between the two normal vectors $\mathbf{n_1}$ and $\mathbf{n_2}$ (see Figure 2B) obtained from MD simulations of Pro-3-*N*-*H* diad **2a** for different simulation replicas. **D**) Similar to **C**), but computed for the Pro-3-*N*-*n*Bu diad **3a**.

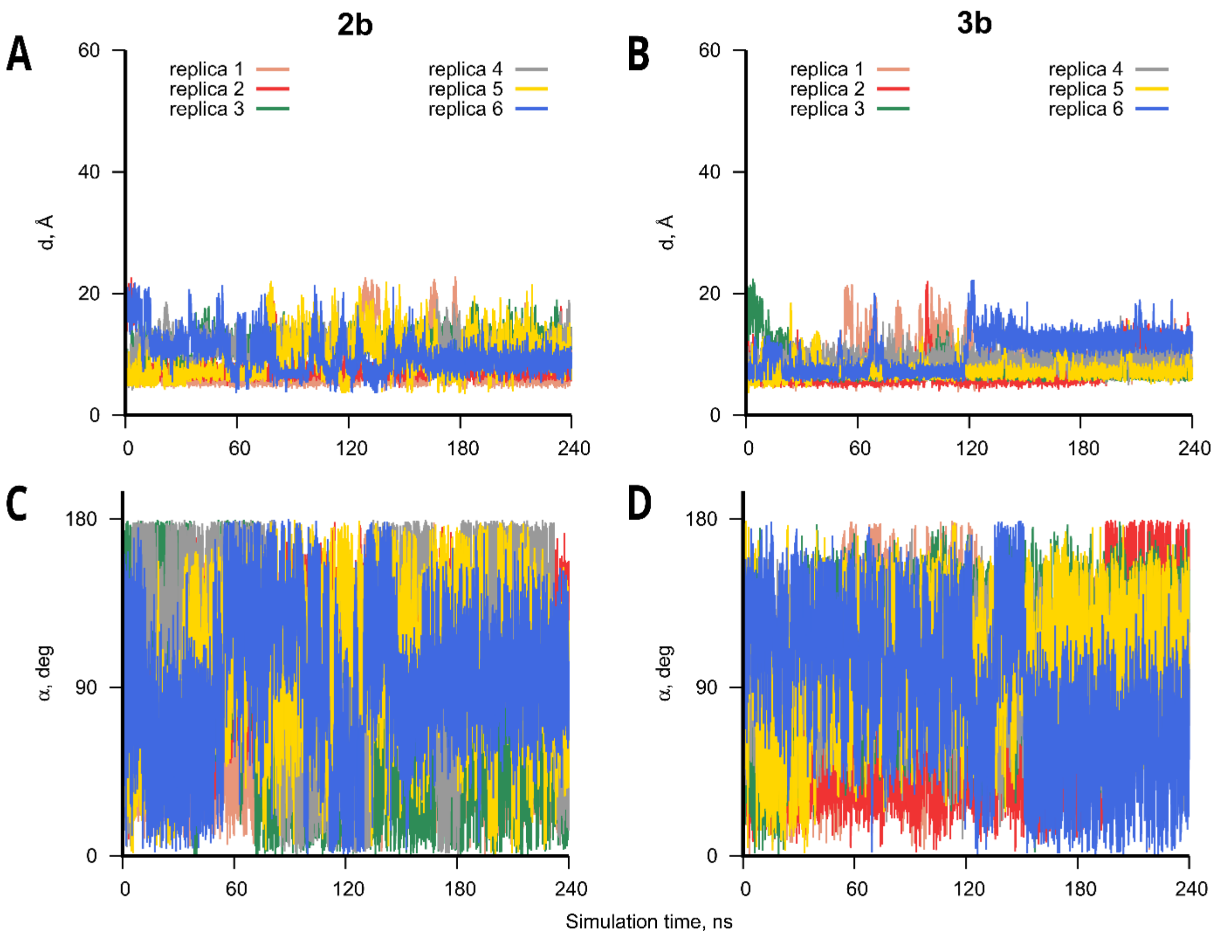

Figure S39: **A**) Temporal evolution of the distance **d** (see Figure 2A) obtained from MD simulations of Pro-4-*N*-*H* diad **2b** for different simulation replicas. **B**) Similar to **A**), but computed for the Pro-4-N-*n*Bu diad **3b**. **C**) Time evolution of the relative angle α defined between the two normal vectors $\mathbf{n_1}$ and $\mathbf{n_2}$ (see Figure 2B) obtained from MD simulations of Pro-4-*N*-*H* diad **2b** for different simulation replicas. **D**) Similar to **C**), but computed for the Pro4-*N*-*n*Bu diad **3b**.

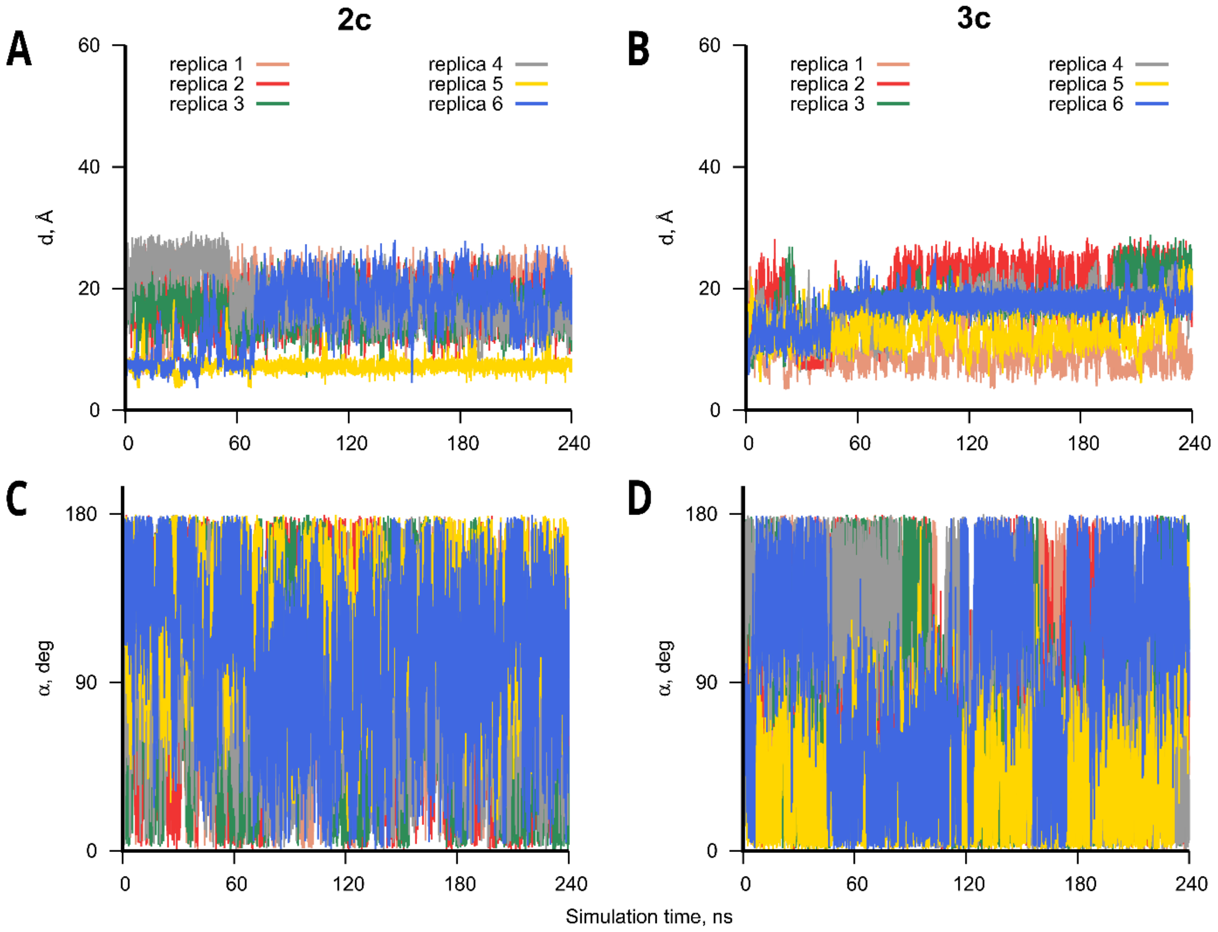

Figure S40: A) Temporal evolution of the distance **d** (see Figure 2A) obtained from MD simulations of Pro6-*N*-*H* diad **2c** for different simulation replicas. **B**) Similar to **A**), but computed for the Pro-6-*N*-*n*Bu diad **3c**. **C**) Time evolution of the relative angle α defined between the two normal vectors $\mathbf{n_1}$ and $\mathbf{n_2}$ (see Figure 2B) obtained from MD simulations of Pro-6-*N*-*H* diad **2c** for different simulation replicas. **D**) Similar to **C**), but computed for the Pro-6-*N*-*n*Bu diad **3c**.

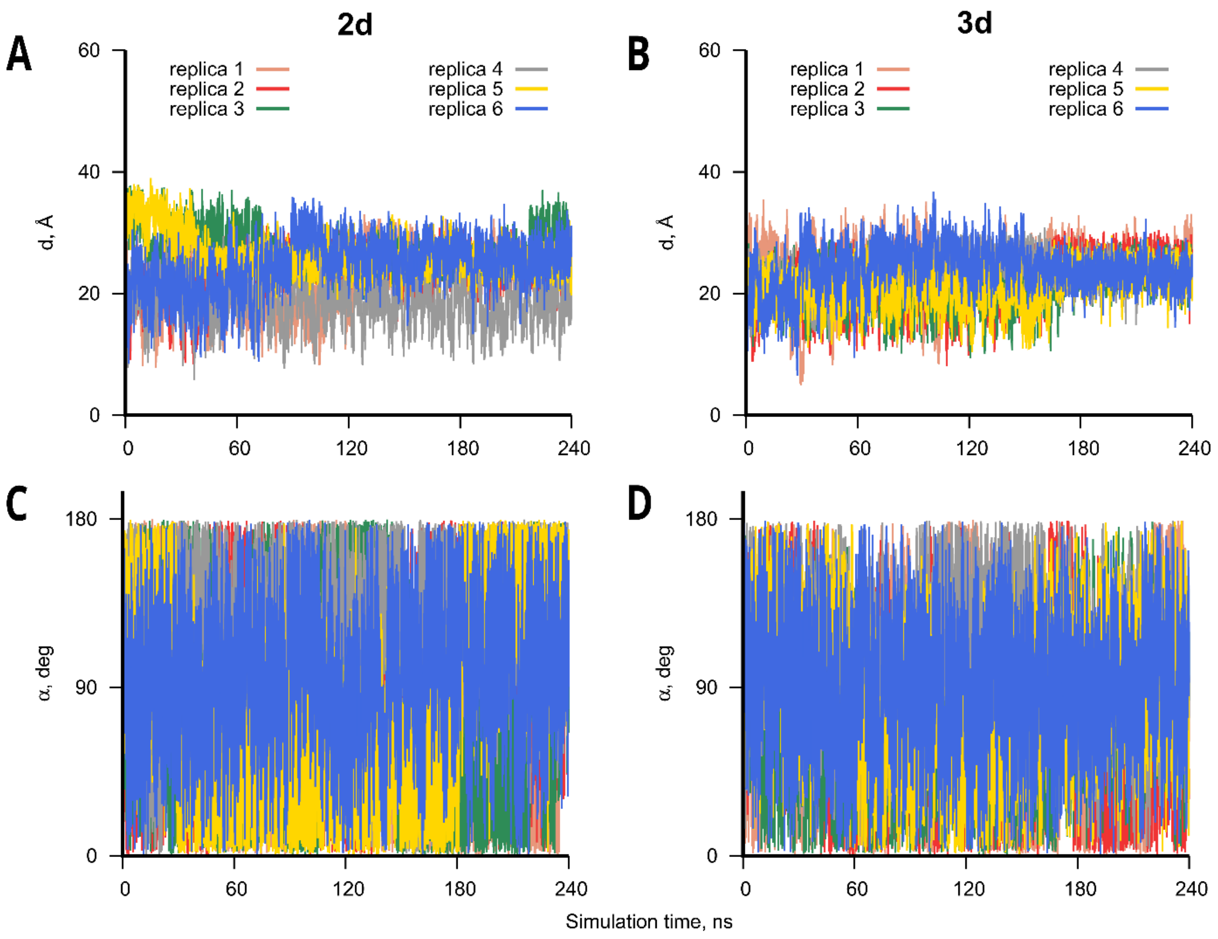

Figure S41: **A**) Temporal evolution of the distance **d** (see Figure 2A) obtained from MD simulations of Pro-9-*N*-*H* diad **2d** for different simulation replicas. **B**) Similar to **A**), but computed for the Pro-9-*N*-*n*Bu diad **3d**. **C**) Time evolution of the relative angle α defined between the two normal vectors $\mathbf{n_1}$ and $\mathbf{n_2}$ (see Figure 2B) obtained from MD simulations of Pro-9-*N*-*H* diad **2d** for different simulation replicas. **D**) Similar to **C**), but computed for the Pro-9-*N*-*n*Bu diad **3d**.

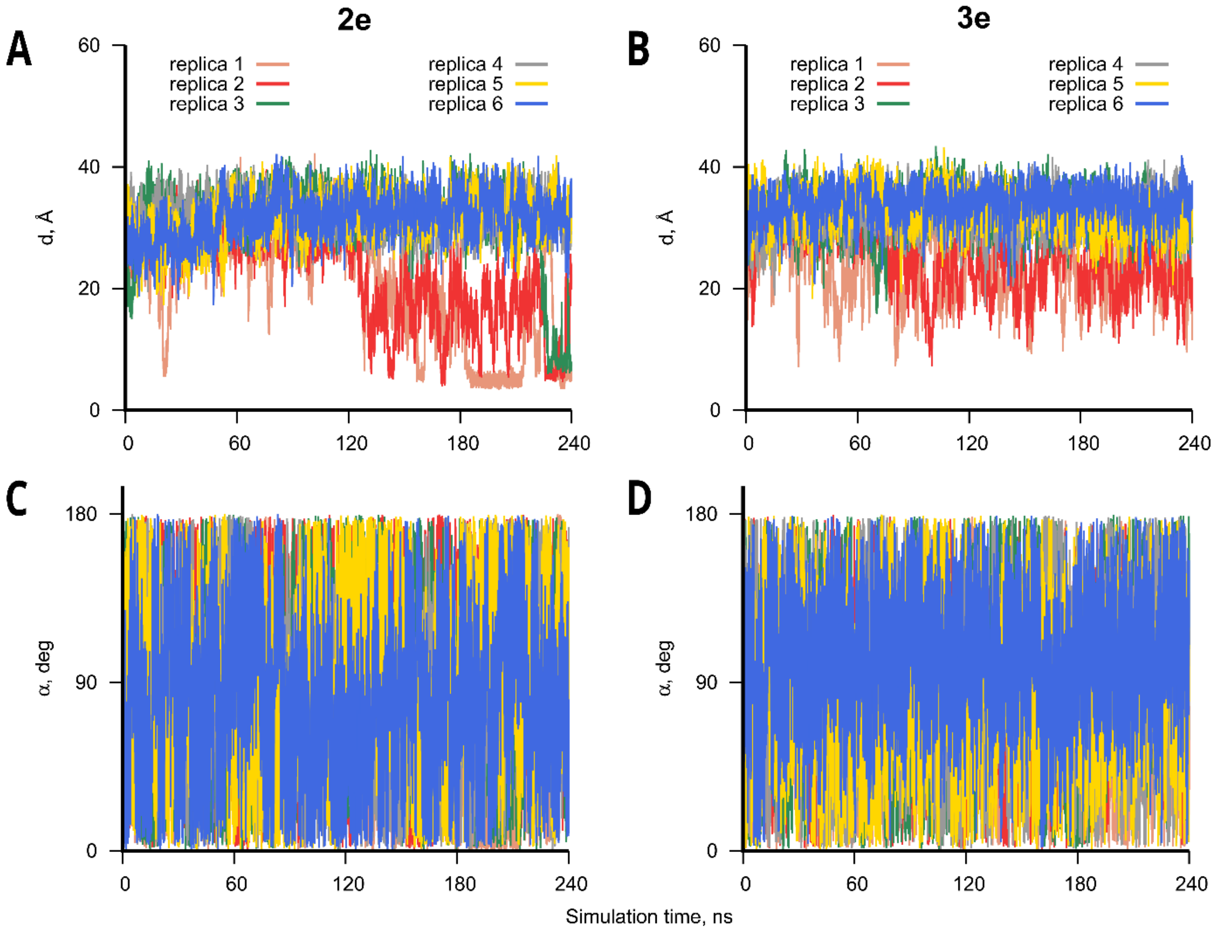

Figure S42: **A**) Temporal evolution of the distance **d** (see Figure 2B) obtained from MD simulations of Pro-12-*N*-*H* diad **2e** for different simulation replicas. **B**) Similar to **A**), but computed for the Pro-12-*N*-*n*Bu diad **3e**. **C**) Time evolution of the relative angle α defined between the two normal vectors $\mathbf{n_1}$ and $\mathbf{n_2}$ (see Figure 2B) obtained from MD simulations of Pro-12-*N*-*H* **2e** for different simulation replicas. **D**) Similar to **C**), but computed for the Pro-12-*N*-*n*Bu diad **3e**.

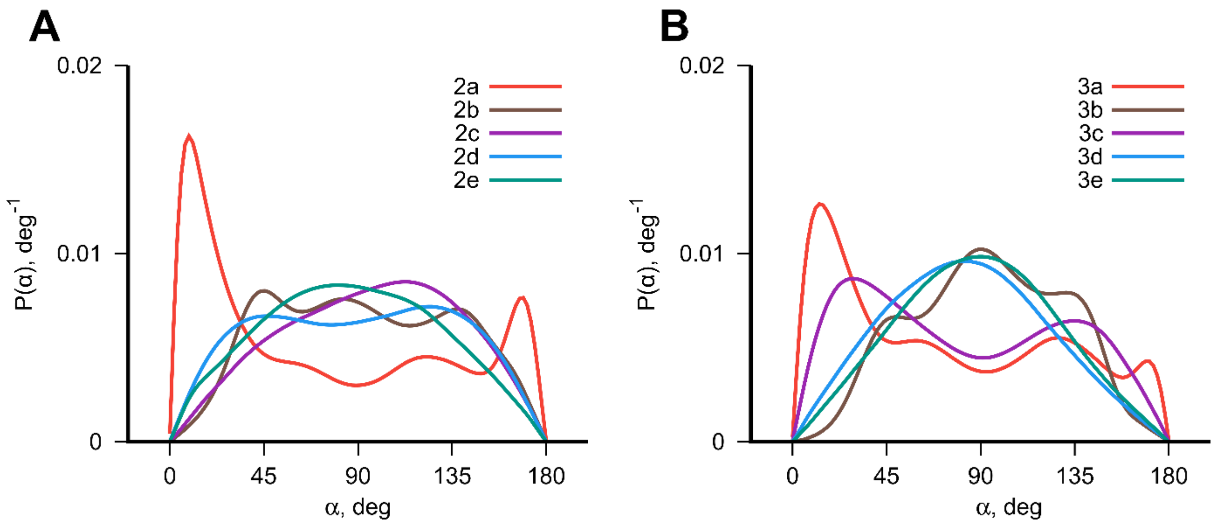

Figure S43: **A**) Probability density describing the distributions of possible relative angles α between the two normal vectors $\mathbf{n_1}$ and $\mathbf{n_2}$ (see Figure 3) obtained from MD simulations, arising in the *N*-*H* diads **2** with the different linker lengths. **B**) Similar to **A**), but computed for the *N*-*n*Bu diads **3** with different linker lengths.

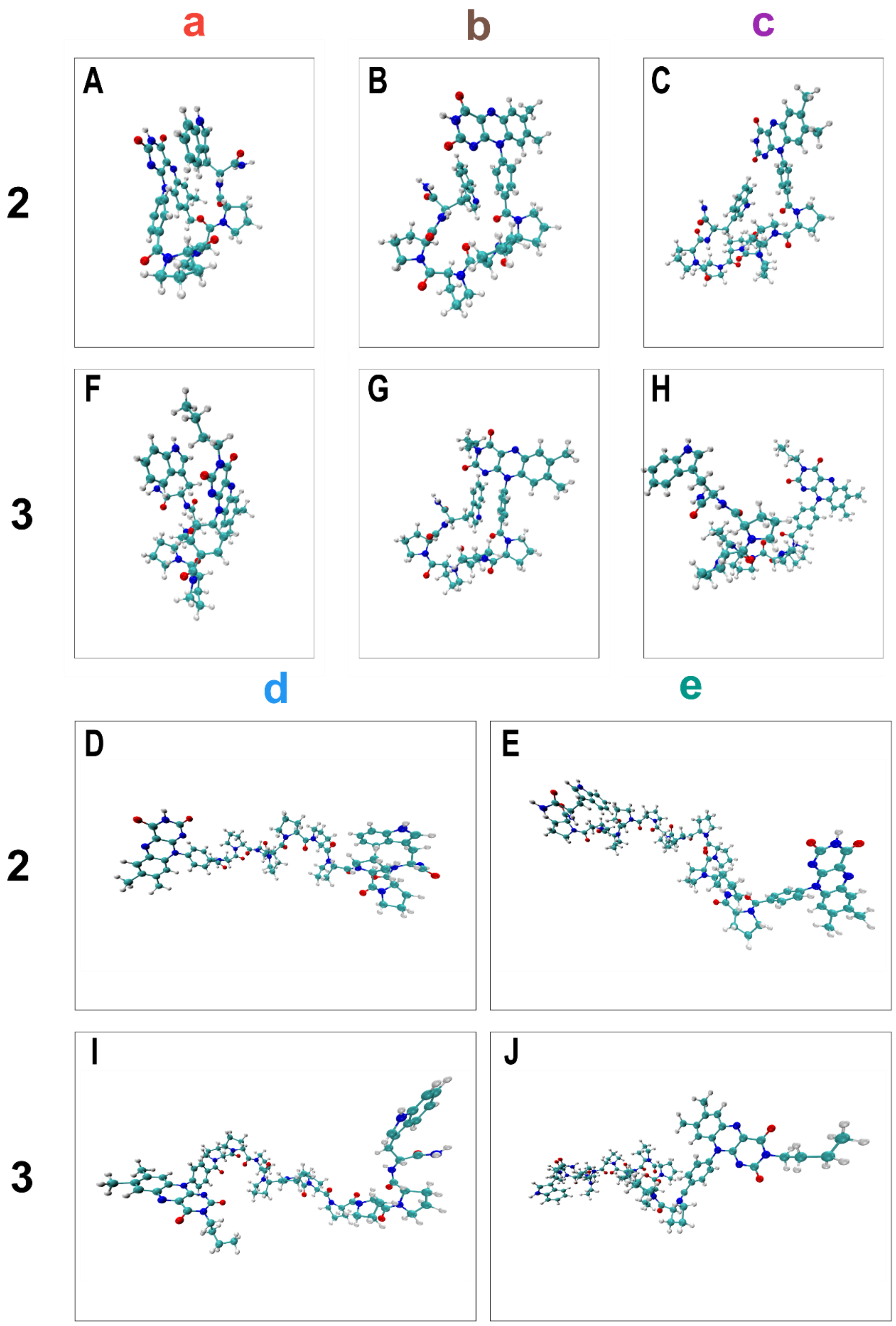

Figure S44: **A**)—**E**) Representative structures corresponding to the most probable values of the distance **d** and angle α (see Figure 2 and Figure S30) for the Pro-3-*N-H* diad **2a**, Pro-4-*N-H* diad **2b**, Pro-6-*N-H* diad **2c**, Pro-9-*N-H* diad **2d**, Pro-12-*N-H* diad **2e**, respectively. **F)**—**J**) Similar to **A—E**), but for the Pro-3-*N*-*n*Bu diad **3a**, Pro-4-*N*-*n*Bu diad **3b**, Pro-6-*N*-*n*Bu diad **3c**, Pro-9-*N*-*n*Bu diad **3d**, Pro-12-*N*-*n*Bu diad **3e**, respectively.

In **Table S5** the peak positions and expectation values of distances for each diadic system are shown. The peak positions and expectation values were obtained by fitting the MD data with a multi-gaussian fitting procedure using the form:

$$f(x) = \sum_{i=1}^{N} a_i \frac{e^{-\frac{(x-\mu_i)^2}{2\sigma_i^2}}}{\sqrt{2\pi}\sigma_i} \qquad (3)$$

Here, a_i are the weight coefficients (sum of a^i normed to 1), μ_i are the peak positions of each maximum, and σ is the variance. As can be seen the center-to-center distances for the **2c** and **3c** diads are similar in their most significant peak values (17.43 / 17.83 Å) and their averaged distance (15.44 / 14.57 Å) to the center-to-center distance of LOV domain proteins.[22-24]

Table S5: Peak position of Trp-flavin distance and expectation value of distance for each diadic system. Peak center-to-center distances are ordered with significance to coefficient when calculating the distance expectation value of each diad.

Diad	Species	Coefficients a_i	Peak μ_i values / Å	⟨Distance⟩ / Å	⟨Distance⟩ / Å from STM
	**a**	0.407 |0.345 | 0.247	4.76 | 7.17 | 17.03	8.63	—
	**b**	0.600 | 0.399	11.30 | 6.63	9.43	—
**2**	**c**	0.802 | 0.197	17.43 | 7.35	15.44	—
	**d**	1	24.62	24.62	3.0 ± 0.5 nm
	**e**	0.812 | 0.187	31.30 |5.62	26.48	—
	**a**	0.578 | 0.421	5.89 | 16.35	10.29	—
	**b**	0.619 | 0.380	9.31 | 6.81	8.36	—
**3**	**c**	0.577 | 0.211 | 0.211	17.83 | 8.44 | 11.76	14.57	2.5 ± 0.5 nm
	**d**	1	23.17	23.17	—
	**e**	0.736 | 0.263	32.81 | 23.50	30.35	—

8. Quantum Chemical Calculations

To study the potential influence on the electronic structure of substituting the hydrogen at the *N3* position with *n*Bu and, furthermore, to compare the electronic properties of our diads with the Trp-Flavin pair of the LOV1 domain, quantum chemical calculations based on density functional theory (DFT) were performed.

The Pro-6 and Pro-9 diads with and without the *N*-*n*Bu substitution were chosen. For the LOV1 domain, the Trp and flavin part of the *Cr*LOV1 protein in its crystal structure (PDB code: 1N9L)[25] were extracted and the dangling bonds were saturated using hydrogen atoms.

As a density functional, the hybrid-functional B3LYP using the basis set Def2-TZVP was chosen, and the implicit solvent model, conductor-like polarizable continuum model (CPCM)[26], was used to mimic the screening effect of the methanol solvent. All diads were geometry optimized based on the best conformer after a CREST[19] analysis. For the LOV1 domain, only the hydrogen atoms were optimized to retain the crystal geometry for comparison. All calculations were performed using ORCA 6.1.0.[26]

To study the influence of the *n*Bu substitution in terms of shifting the redox potential, the HOMO-LUMO energy gap was calculated for both diad lengths with their N-H and N-nBu variants. **Table S6** shows the calculated values for the HOMO-LUMO gap.

Table S6: Energy gap $\Delta E_{HOMO\text{-}LUMO}$ between the HOMO and LUMO of four respective diads in comparison to the Trp and flavin of the *Cr*LOV1 crystal structure.

Proline chain	Diad	HOMO / a.u.	LUMO / a.u.	$\Delta E_{HOMO\text{-}LUMO}$ / eV
Pro-6	(**2c**) H	-0.207244	-0.113069	2.56
	(**3c**) *n*Bu	-0.206057	-0.111472	2.57
Pro-9	(**2d**) H	-0.206312	-0.113345	2.53
	(**3d**) *n*Bu	-0.206131	-0.111388	2.58
-	*Cr*LOV1	-0.207706	-0.115492	2.51

As can be observed, the substitution only marginally changes the HOMO-LUMO energy gap, indicating that the modification of the diads only provides benefit for structural separation of diads within solution. Furthermore, the calculated HOMO-LUMO gaps are in good agreement with the LOV1 domain, however, one should note that the protein environment is not considered here, which may polarize the system significantly.[27]

Another aspect that can reveal the suitability of the synthesised diads to mimic a desired biological system is to calculate the overlap between the molecular orbitals of the Trp and flavin. In the ground state, this overlap is an important property that guides the electron transfer necessary for forming the spin-correlated radical pair. In the excited charge-transfer (resembling the spin-correlated radical pair), the overlap provides an indication of the potential exchange interaction (even though this interpretation must be taken with care, as the exchange interaction does not necessarily rely on the spatial distribution of the molecular orbitals).

In the framework of DFT, the description of an open-shell singlet charge-transfer state is not accurately possible as the single reference wavefunction would not be reliable, and a multiconfiguration approach must be applied.[28] However, a sufficient approximation is to study the charge-transfer triplet state using DFT.[29] Here, it is important to guarantee that the correct triplet state is received while optimizing the system, which can be obtained by investigating the spin density of the respective radical pairs. **Figure S45** illustrates an exemplary spin density of the simulated triplet states for the Pro-6 diads, Pro-9 diads, and the LOV1 domain.

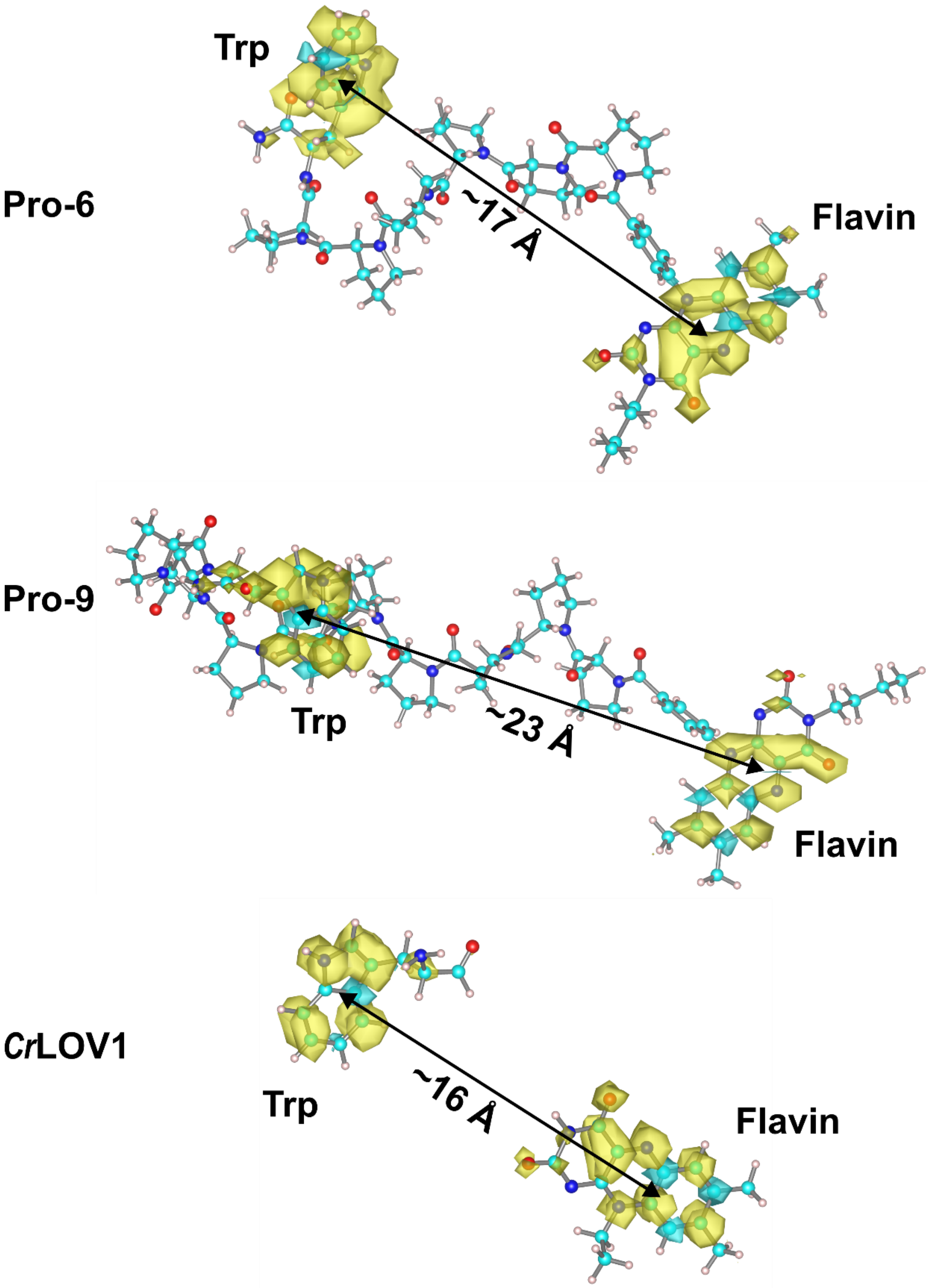

Figure S45: Exemplary illustration of spin density localization for the two diad lengths (here *N*-*n*Bu, **3c** and **3d**) and the Trp and flavin of the crystal structure of *Cr*LOV1 in their respective charge-transfer triplet states. In the geometry optimized structure, the **3c** diad reaches a similar center-to-center distance as the for the *Cr*LOV1 scenario.

To calculate the orbital overlap between the two molecular moieties, the Mulliken fragmented orbital overlap approach was employed.[30] Here, the overlap matrix $S_{\mu\nu}$ with atomic orbitals μ and ν of the full system, is partitioned into chosen fragments (in our case the flavin and Trp part of the diads/LOV1 domain) and two new overlap matrices are constructed:

$$S_{\mu\nu}^{(A)} = \frac{1}{2}\left(\delta_\mu^A + \delta_\nu^A\right)S_{\mu\nu} \text{ with } \delta_\mu^A = \begin{cases} 1 & \mu \in A \\ 0 & \text{else} \end{cases} \qquad (4)$$

$$S_{\mu\nu}^{(AB)} = \frac{1}{2}(\delta_\mu^A \delta_\nu^B + \delta_\mu^B \delta_\nu^A)S_{\mu\nu} \qquad (5)$$

The matrix $S_{\mu\nu}^{(A)}$ contains all overlaps where at least one atomic orbital belongs to fragment A, while matrix $S_{\mu\nu}^{(AB)}$ contains only overlaps where the two atomic orbitals μ and ν sit on different fragments A and B. The population of overlap is then received by using the columns of the occupied (occ_i) molecular orbital coefficients C_i in the AO basis:

$$P_{ii}^{AB} = occ_i C_i^T S^{AB} C_i \qquad (6)$$

The absolute sum of all orbital overlap population can than be used as an indication for overlap between the two fragments. The obtained overlaps for the ground state (GS) and charge-transfer (CT) triplet state are presented in **Table S7**.

Table S7: Orbital overlap between Trp and flavin for different diads and different states compared to the Trp and flavin of the *Cr*LOV1 crystal structure. Here, GS is the singlet ground state and CT triplet is the charge-transfer triplet state. No orbital overlap can be found for the diads while also for the *Cr*LOV1 only a marginal value is calculated.

Proline chain	Diad	State	Orbital overlap
Pro-6	(2c) H	GS	0.0000
		CT triplet	0.0000
	(3c) *n*Bu	GS	0.0000
		CT triplet	0.0000
Pro-9	(2d) H	GS	0.0000
		CT triplet	0.0000
	(3d) *n*Bu	GS	0.0000
		CT triplet	0.0000
-	LOV1	GS	0.0011
		CT triplet	0.0022

It becomes apparent that no significant orbital overlap can be found in all scenarios. The result suggests that the electron transfer may be mediated through the proline chain or the flexibility of the diadic systems. In the *Cr*LOV1 domain, it is likely that the electron transfer may be similar to that

transferred through residues in close vicinity or vibronic effects. The excited state S1 after illumination of the diadic system is, however, not considered here, which may reveal a much stronger orbital overlap. Comparison with other flexible diadic systems indicates that the flexibility of the diads may also be crucial for a sufficient electron transfer.[31]

9. Scanning Tunneling Microscopy (STM) Imaging

A droplet of 1µL of the diad **2c** and **3c**, respectively, in ethanol (1 mM) was deposited on highly ordered pyrolytic graphite (HOPG, 0001) surfaces. All scanning tunneling microscopy (STM) experiments were performed under ambient conditions at room temperature by by using RHK SPM 100 and mechanically made Platinum-Iridium (Pt/Ir) tips. The tunneling parameters were U_t = 1.5 V and I_t = 1.0 nA.

STM image of the *N-H* diads **2** showed a well-ordered lamellar film with the size of 6 nm ± 0.5 nm (for **2c**), where the Trp and the flavin moiety of two diad molecules are connected by hydrogen bonding.

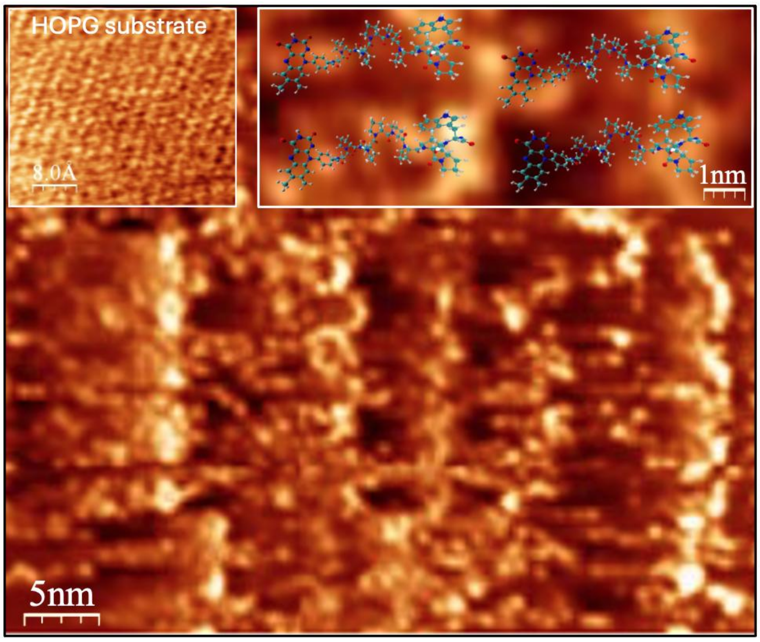

Figure S46: STM image of *N-H* diad **2d** diad. A droplet of 1 µL of each diad in ethanol (1 mM) was deposited on the HOPG (0001) surface (shown in inset in A). All STM experiments were performed under ambient conditions at room temperature by using mechanically made Pt/Ir tips. The tunneling parameters were U_t = 1.5 V and I_t = 1.0 nA. The molecular rendering in the insets illustrates the configuration of some typical conformations of the diads, as obtained from MD simulations (see below).

The *N*-alkylated variant **3** formed ring-like structures with the cavities of each ring varying from 2.5 nm to 7.0 nm ± 0.2 nm for **3c**. This observation hints at the partial disruption of the Trp-flavin hydrogen bonding network.

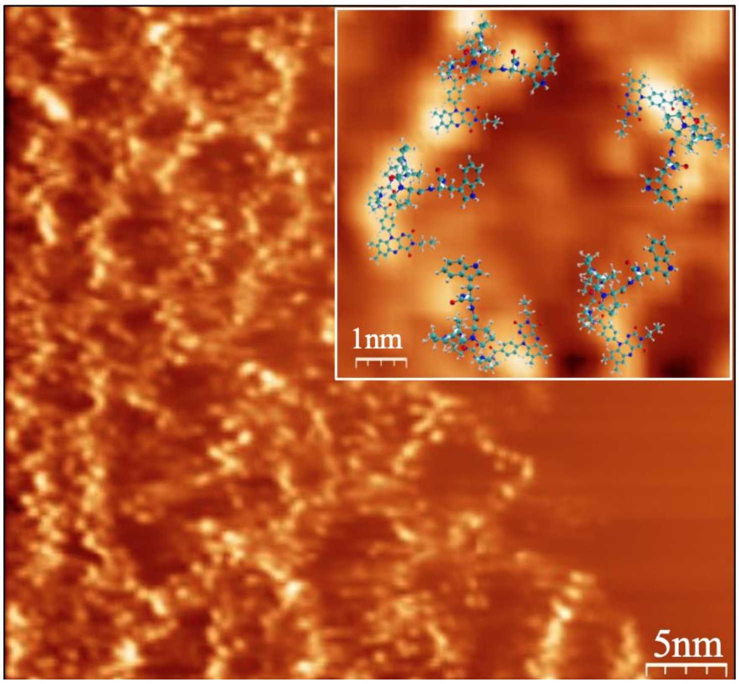

Figure S47: STM image of *N*-alkylated **3c** diad. A droplet of 1 µL of each diad in ethanol (1 mM) was deposited on the HOPG (0001) surface (shown in inset in A). All STM experiments were performed under ambient conditions at room temperature by using mechanically made Pt/Ir tips. The tunneling parameters were U_t = 1.5 V and I_t = 1.0 nA. The molecular rendering in the insets illustrates the configuration of some typical conformations of the diads, as obtained from MD simulations (see below).

10. UV-vis spectroscopy

Absorption experiments were performed on a Shimadzu 1900i spectrometer at ambient temperature. The spectra of the diads **2a** and **3a** were normalized to the maximum absorption intensity.

In the first spectrum (Figure S48), a comparison of compounds **3a** and **2a** reveals a small red shift (~3 nm) in the main absorption band upon replacement of the *N*-*n*Bu substituent with an *N-H* group. A slight decrease in intensity is also observed for **2a** in the ~340 nm band, indicating a potential influence of the *N*-substitution on the electronic structure of the chromophore.

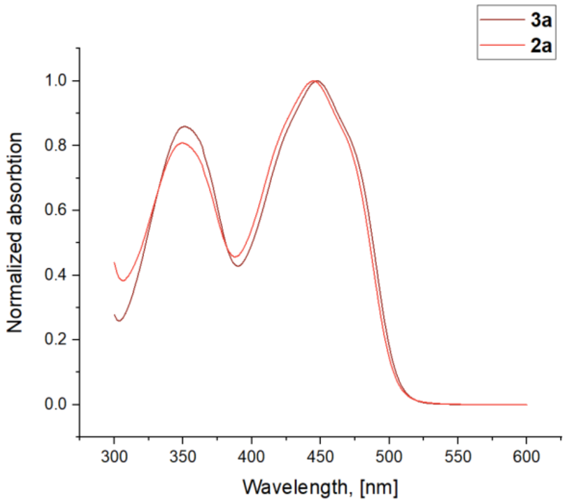

Figure S48: Normalized UV/Vis absorption spectra of **3a** vs. **2a** in methanol. Spectra were recorded at room temperature.

In the second spectrum (Figure S49), a similar trend is observed for the **3d** and **2d** pair. The red shift and intensity variation are consistent with those observed for the *N3*-substituted analogs, suggesting that the effect of the *N*-substitution on the absorption properties is general across the series.

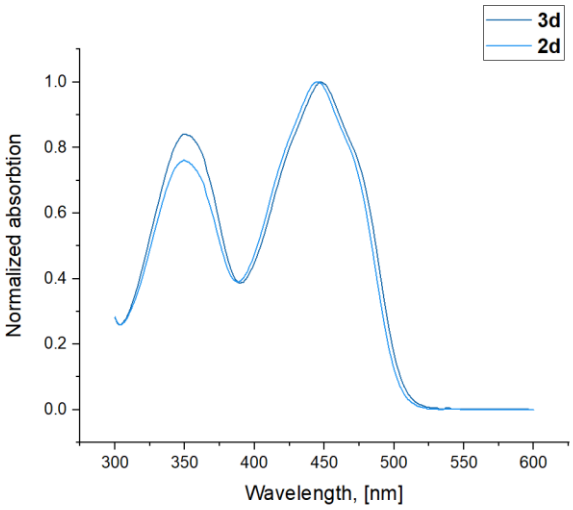

Figure S49: Normalized UV/Vis absorption spectra of **3d** vs. **2d** in methanol. Spectra were recorded at room temperature.

The third spectrum (Figure S50) compares the *N-H*-substituted diads with varying linker lengths (3, 4, 6, 9, and 12 prolines). All samples show similar absorption features with minor variations in the intensity of the 340 nm band. Importantly, no significant shift in the absorption maxima is observed across the series, indicating that changes in linker length do not strongly affect the ground-state electronic properties of the chromophore.

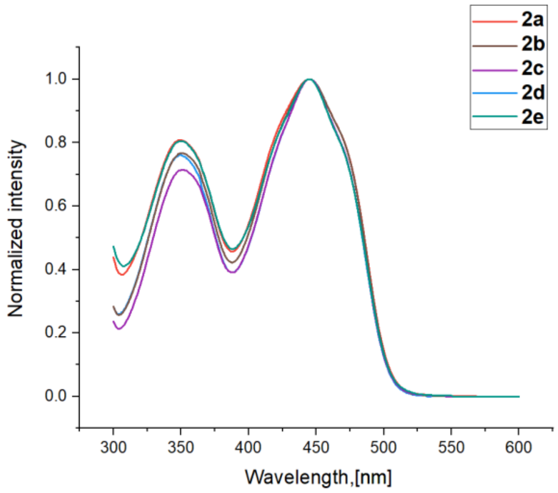

Figure S50: Normalized UV/Vis absorption spectra of *N-H*-substituted diads with varying linker lengths (3–12 prolines) in methanol. Spectra were recorded at room temperature.

11. References

[1] Y. Oka, K. Inoue, "Time-resolved EPR observation of blue-light-induced radical ion pairs in a flavin–Trp dyad" *Phys. Chem. Chem. Phys.* **2024**, *26*, 16444-16448.

[2] L. A. García, E. Arias, I. Moggio, J. Romero, A. Ledezma, A. Ponce, O. Perez, "Fluorescent core-sheath fibers by electrospinning of a phenyleneethynylene/poly(styrene-co-maleimide) blend" *Polymer* **2011**, *52*, 5326-5334.

[3] F. Rabanal, M. D. Ludevid, M. Pons, E. Giralt, "CD of proline-rich polypeptides: Application to the study of the repetitive domain of maize glutelin-2" *Biopolymers* **1993**, *33*, 1019-1028.

[4] B. Koronkiewicz, J. Swierk, K. Regan, J. M. Mayer, "Shallow Distance Dependence for Proton-Coupled Tyrosine Oxidation in Oligoproline Peptides" *J. Am. Chem. Soc.* **2020**, *142*, 12106-12118.

[5] J. C. Phillips, D. J. Hardy, J. D. C. Maia, J. E. Stone, J. V. Ribeiro, R. C. Bernardi, R. Buch, G. Fiorin, J. Hénin, W. Jiang, R. McGreevy, M. C. R. Melo, B. K. Radak, R. D. Skeel, A. Singharoy, Y. Wang, B. Roux, A. Aksimentiev, Z. Luthey-Schulten, L. V. Kalé, K. Schulten, C. Chipot, E. Tajkhorshid, "Scalable molecular dynamics on CPU and GPU architectures with NAMD" *J. Chem. Phys.* **2020**, *153*, 044130.

[6] J. C. Phillips, R. Braun, W. Wang, J. Gumbart, E. Tajkhorshid, E. Villa, C. Chipot, R. D. Skeel, L. Kalé, K. Schulten, "Scalable molecular dynamics with NAMD" *J. Comput. Chem.* **2005**, *26*, 1781-1802.

[7] J. Huang, S. Rauscher, G. Nawrocki, T. Ran, M. Feig, B. L. de Groot, H. Grubmüller, A. D. MacKerell, "CHARMM36m: an improved force field for folded and intrinsically disordered proteins" *Nat. Methods* **2017**, *14*, 71-73.

[8] R. B. Best, X. Zhu, J. Shim, P. E. M. Lopes, J. Mittal, M. Feig, A. D. MacKerell, Jr., "Optimization of the Additive CHARMM All-Atom Protein Force Field Targeting Improved Sampling of the Backbone ϕ, ψ and Side-Chain $\chi1$ and $\chi2$ Dihedral Angles" *J. Chem. Theory Comp.* **2012**, *8*, 3257-3273.

[9] B. R. Brooks, C. L. Brooks Iii, A. D. Mackerell Jr, L. Nilsson, R. J. Petrella, B. Roux, Y. Won, G. Archontis, C. Bartels, S. Boresch, A. Caflisch, L. Caves, Q. Cui, A. R. Dinner, M. Feig, S. Fischer, J. Gao, M. Hodoscek, W. Im, K. Kuczera, T. Lazaridis, J. Ma, V. Ovchinnikov, E. Paci, R. W. Pastor, C. B. Post, J. Z. Pu, M. Schaefer, B. Tidor, R. M. Venable, H. L. Woodcock, X. Wu, W. Yang, D. M. York, M. Karplus, "CHARMM: The biomolecular simulation program" *J. Comput. Chem.* **2009**, *30*, 1545-1614.

[10] A. D. MacKerell, Jr., M. Feig, C. L. Brooks, "Improved Treatment of the Protein Backbone in Empirical Force Fields" *J. Am. Chem. Soc.* **2004**, *126*, 698-699.

[11] A. D. MacKerell, Jr., D. Bashford, M. Bellott, R. L. Dunbrack, Jr., J. D. Evanseck, M. J. Field, S. Fischer, J. Gao, H. Guo, S. Ha, D. Joseph-McCarthy, L. Kuchnir, K. Kuczera, F. T. K. Lau, C. Mattos, S. Michnick, T. Ngo, D. T. Nguyen, B. Prodhom, W. E. Reiher, B. Roux, M. Schlenkrich, J. C. Smith, R. Stote, J. Straub, M. Watanabe, J. Wiórkiewicz-Kuczera, D. Yin, M. Karplus, "All-

Atom Empirical Potential for Molecular Modeling and Dynamics Studies of Proteins" *J. Phys. Chem. B* **1998**, *102*, 3586-3616.

[12] W. Yu, X. He, K. Vanommeslaeghe, A. D. MacKerell Jr, "Extension of the CHARMM general force field to sulfonyl-containing compounds and its utility in biomolecular simulations" *J. Comput. Chem.* **2012**, *33*, 2451-2468.

[13] K. Vanommeslaeghe, E. Hatcher, C. Acharya, S. Kundu, S. Zhong, J. Shim, E. Darian, O. Guvench, P. Lopes, I. Vorobyov, A. D. Mackerell Jr, "CHARMM general force field: A force field for drug-like molecules compatible with the CHARMM all-atom additive biological force fields" *J. Comput. Chem.* **2010**, *31*, 671-690.

[14] G. Lüdemann, I. A. Solov'yov, T. Kubař, M. Elstner, "Solvent Driving Force Ensures Fast Formation of a Persistent and Well-Separated Radical Pair in Plant Cryptochrome" *J. Am. Chem. Soc.* **2015**, *137*, 1147-1156.

[15] I. A. Solov'yov, T. Domratcheva, A. R. Moughal Shahi, K. Schulten, "Decrypting Cryptochrome: Revealing the Molecular Identity of the Photoactivation Reaction" *J. Am. Chem. Soc.* **2012**, *134*, 18046-18052.

[16] M. J. Frisch, Trucks, G.W., Schlegel, H.B., Scuseria, G.E., Robb, M.A., Cheeseman, J.R., Scalmani, G., Barone, V., Mennucci, B., Petersson, G.A., Nakatsuji, H., Caricato, M., Li, X., Hratchian, H.P., Izmaylov, A.F., Bloino, J., Zheng, G., Sonnenberg, J.L., Hada, M., Ehara, M., Toyota, K., Fukuda, R., Hasegawa, J., Ishida, M., Nakajima, T., Honda, Y., Kitao, O., Nakai, H., Vreven, T., Montgomery Jr., J.A., Peralta, J.E., Ogliaro, F., Bearpark, M., Heyd, J.J., Brothers, E., Kudin, K.N., Staroverov, V.N., Kobayashi, R., Normand, J., Raghavachari, K., Rendell, A., Burant, J.C., Iyengar, S.S., Tomasi, J., Cossi, M., Rega, N., Millam, J.M., Klene, M., Knox, J.E., Cross, J.B., Bakken, V., Adamo, C., Jaramillo, J., Gomperts, R., Stratmann, R.E., Yazyev, O., Austin, A.J., Cammi, R., Pomelli, C., Ochterski, J.W., Martin, R.L., Morokuma, K., Zakrzewski, V.G., Voth, G.A., Salvador, P., Dannenberg, J.J., Dapprich, S., Daniels, A.D., Farkas, O., Foresman, J.B., Ortiz, J.V., Cioslowski, J. and Fox, D.J. , *Gaussian 09, Revision B.01.*, **2010**

[17] G. J. Martyna, D. J. Tobias, M. L. Klein, "Constant pressure molecular dynamics algorithms" *J. Chem. Phys.* **1994**, *101*, 4177-4189.

[18] S. E. Feller, Y. Zhang, R. W. Pastor, B. R. Brooks, "Constant pressure molecular dynamics simulation: The Langevin piston method" *J. Chem. Phys.* **1995**, *103*, 4613-4621.

[19] P. Pracht, F. Bohle, S. Grimme, "Automated exploration of the low-energy chemical space with fast quantum chemical methods" *Phys. Chem. Chem. Phys.* **2020**, *22*, 7169-7192.

[20] S. Grimme, "Exploration of Chemical Compound, Conformer, and Reaction Space with Meta-Dynamics Simulations Based on Tight-Binding Quantum Chemical Calculations" *J. Chem. Theory Comp.* **2019**, *15*, 2847-2862.

[21] W. Humphrey, A. Dalke, K. Schulten, "VMD: Visual molecular dynamics" *J. Mol. Graph.* **1996**, *14*, 33-38.

[22] Y. Ding, A. S. Kiryutin, A. V. Yurkovskaya, D. V. Sosnovsky, R. Z. Sagdeev, S. Bannister, T. Kottke, R. K. Kar, I. Schapiro, K. L. Ivanov, J. Matysik, "Nuclear spin-hyperpolarization generated in a flavoprotein under illumination: experimental field-dependence and theoretical level crossing analysis" *Sci. Rep.* **2019**, *9*, 18436.

[23] T. J. El-Baba, D. R. Fuller, D. A. Hales, D. H. Russell, D. E. Clemmer, "Solvent Mediation of Peptide Conformations: Polyproline Structures in Water, Methanol, Ethanol, and 1-Propanol as Determined by Ion Mobility Spectrometry-Mass Spectrometry" *J. Am. Soc. Mass. Spectrom.* **2019**, *30*, 77-84.

[24] S. N. Batchelor, H. Fischer, "Radical Addition Rates to Alkenes by Time-Resolved CIDNP: 2-Hydroxy-2-propyl Radicals" *J. Phys. Chem.* **1996**, *100*, 9794-9799.

[25] P. Kurle-Tucholski, L. Gerhards, Y. Ding, Y. Kim, I. S. Anisimova, A. Alia, I. A. Solov'yov, J. Matysik, "Nuclear hyperpolarization in electron-transfer proteins: Revealing unexpected light-induced 15N signals with field-cycling magic-angle spinning NMR" *Journal of Magnetic Resonance Open* **2024**, *21*, 100168.

[26] F. Neese, "Software Update: The ORCA Program System—Version 6.0" *WIREs Computational Molecular Science* **2025**, *15*, e70019.

[27] A. Frederiksen, L. Gerhards, P. Reinholdt, J. Kongsted, I. A. Solov’yov, "Importance of Polarizable Embedding for Absorption Spectrum Calculations of Arabidopsis thaliana Cryptochrome 1" *J. Phys. Chem. B* **2024**, *128*, 6283-6290.

[28] L. Gerhards, M. Werr, O. Hübner, I. A. Solov’yov, H.-J. Himmel, "Peculiar Differences between Two Copper Complexes Containing Similar Redox-Active Ligands: Density Functional and Multiconfigurational Calculations" *Inorg. Chem.* **2024**, *63*, 961-975.

[29] L. Gerhards, T. Klüner, "Theoretical investigation of CH-bond activation by photocatalytic excited SO2 and the effects of C-, N-, S-, and Se-doped TiO2" *Phys. Chem. Chem. Phys.* **2022**, *24*, 2051-2069.

[30] R. S. Mulliken, "Electronic Population Analysis on LCAO–MO Molecular Wave Functions. II. Overlap Populations, Bond Orders, and Covalent Bond Energies" *J. Chem. Phys.* **1955**, *23*, 1841-1846.

[31] D. Timmer, G. Hergert, L. Gerhards, D. C. Lünemann, N. Schröder, T. Greven, J. I. van der Vlugt, A. De Sio, I. A. Solov’yov, J. Christoffers, C. Lienau, "Structural Flexibility Slows Down Charge Transfers in Diaminoterephthalate-C60 Dyads" *The Journal of Physical Chemistry C* **2024**, *128*, 2380-2391.
